# Exploring protein relative relations in skeletal muscle proteomic analysis for insights into insulin resistance and type 2 diabetes

**DOI:** 10.1038/s41598-024-68568-4

**Published:** 2024-07-31

**Authors:** Anna Czajkowska, Marcin Czajkowski, Lukasz Szczerbinski, Krzysztof Jurczuk, Daniel Reska, Wojciech Kwedlo, Marek Kretowski, Piotr Zabielski, Adam Kretowski

**Affiliations:** 1 Clinical Research Centre, Medical University of Bialystok, Białystok, Poland; 2 Department of Medical Biology, Medical University of Bialystok, A. Mickiewicza 2C, 15-369 Białystok, Poland; 3 Faculty of Computer Science, Bialystok University of Technology, Białystok, Poland; 4 Department of Endocrinology, Diabetology and Internal Medicine, Medical University of Bialystok, Białystok, Poland; 5 Programs in Metabolism and Medical and Population Genetics, Broad Institute of Harvard and MIT, Cambridge, MA USA

**Keywords:** Diabetes, Computational science, Biomarkers, Proteomics, Type 2 diabetes

## Abstract

The escalating prevalence of insulin resistance (IR) and type 2 diabetes mellitus (T2D) underscores the urgent need for improved early detection techniques and effective treatment strategies. In this context, our study presents a proteomic analysis of post-exercise skeletal muscle biopsies from individuals across a spectrum of glucose metabolism states: normal, prediabetes, and T2D. This enabled the identification of significant protein relationships indicative of each specific glycemic condition. Our investigation primarily leveraged the machine learning approach, employing the white-box algorithm relative evolutionary hierarchical analysis (REHA), to explore the impact of regulated, mixed mode exercise on skeletal muscle proteome in subjects with diverse glycemic status. This method aimed to advance the diagnosis of IR and T2D and elucidate the molecular pathways involved in its development and the response to exercise. Additionally, we used proteomics-specific statistical analysis to provide a comparative perspective, highlighting the nuanced differences identified by REHA. Validation of the REHA model with a comparable external dataset further demonstrated its efficacy in distinguishing between diverse proteomic profiles. Key metrics such as accuracy and the area under the ROC curve confirmed REHA’s capability to uncover novel molecular pathways and significant protein interactions, offering fresh insights into the effects of exercise on IR and T2D pathophysiology of skeletal muscle. The visualizations not only underscored significant proteins and their interactions but also showcased decision trees that effectively differentiate between various glycemic states, thereby enhancing our understanding of the biomolecular landscape of T2D.

## Introduction

Type 2 diabetes mellitus (T2D) is a global health challenge characterized by elevated blood glucose levels and defective glucose metabolism in adipose, hepatic and skeletal muscle tissues. T2D is marked by abnormal glucose, protein and lipid metabolism, which arises from insulin resistance and insufficient hormonal response, leading to progressive pancreatic beta cell dysfunction. This disease not only affects millions worldwide but also imposes significant clinical, social, and economic burdens^1–4^.

With the global incidence of diabetes on the rise, there is an urgent need for earlier diagnosis and more effective treatment strategies. Modern lifestyle factors, such as reduced physical activity and unhealthy dietary habits, contribute significantly to the increasing prevalence of the condition. Skeletal muscle, as the largest tissue mass in the body, plays a central role in whole-body energy and glucose metabolism under both physiological and pathological conditions^5^. It adapts to various stimuli, changing in size, fiber type, and metabolic properties, and is pivotal in glucose metabolism and insulin sensitivity^6,7^.

Recent advances in omics technologies, particularly proteomics, have been instrumental in identifying and quantifying proteins and their modifications, offering insights into disease mechanisms. These high-throughput techniques enable the exploration of altered protein expression profiles and molecular pathways contributing to insulin resistance and T2D. However, the complexity and high dimensionality of proteomics data necessitates the use of Machine Learning methods not only at the level of protein identification and quantitation, but also interpretation of biological relationships.

In this study, we focus on using a dedicated machine learning approach, the relative evolutionary hierarchical analysis (REHA), which integrates elements of the decision tree model and evolutionary algorithms. REHA’s main innovation lies in its use of the Relative eXpression Analysis (RXA)^8,9^ and the multi-test concept^10^, based on clusters of co-expressed proteins. This approach allows us to explore associations among proteins identified by untargeted skeletal muscle proteomics, advancing both the understanding of their biological relationships and biomarker discovery^11^.

Our research is focused on improving the accuracy of IR and type 2 diabetes (T2D) predictions, while also deepening our understanding of the impact of exercise on key molecular pathways involved in the disease’s progression. We focus on protein expression alterations in skeletal muscle among individuals with normal, prediabetic, and diabetic metabolic states following a regulated exercise intervention. This approach enables us to investigate changes in protein expression associated with the response to exercise in IR and T2D, elucidating the roles of proteins and signaling pathways in skeletal muscle tissue. This may potentially lead to the identification of new therapeutic targets for insulin resistance and related metabolic disorders.

In this study, we assessed post-exercise skeletal muscle biopsies collected from patients with distinct glycemic state disturbances: 13 with normoglycemia, 11 with prediabetes, and 8 with T2D. Analysis was conducted on 2 independent biopsies from each participant (total of 64 samples), leading to identification of 1139 proteins, after stringent application of FDR, significance and differential expression fold-change cutoffs. Additionally, our study incorporated data from 42 samples from public repository, ranging from prediabetic to healthy states, further enriching our understanding of the protein dynamics in various glycemic conditions.

To validate our machine learning-based findings, we employed Statistics tailored for proteome data analysis. For the comparison between the NG, PD, and T2D groups, only proteins present in at least 50% of the samples (Q-value percentile 0.5), with a minimum of 2 unique peptides per protein group, displaying at least 50% expression difference (− 0.585 ≤ log2 ≤ 0.585), and possessing an FDR-adjusted *p*-value < 0.05 (Q-value < 0.05, − Log10 *p*-val > 1.13), were considered as significant^12^.

This statistical validation was essential for identifying potential biomarkers and for providing a one-dimensional view to complement the multi-dimensional insights offered by the REHA algorithm. We compared these statistical results with those obtained from REHA to draw comprehensive conclusions about the protein expressions in different glycemic states.

Furthermore, our investigation was enhanced through a robust external dataset, from the experiment performed on comparable study group. We integrated data from a proteomic analysis of skeletal muscle that was aligned with our classification groups, focusing on 645 proteins shared between both datasets. This step was crucial for validating the performance of the REHA models against external data, which not only demonstrated the versatility of our approach but also provided insights into the generalizability of our findings.

In addition to REHA, we tested several popular machine learning algorithms, both white-box and black-box approaches^13^. These diverse methodologies allowed us to thoroughly evaluate and compare the effectiveness of different analytical approaches in handling high-dimensional proteomics data. The performance of each algorithm, including REHA, was rigorously validated using metrics such as F1-score, weighted F1-score, accuracy, and the area under the ROC curve.

The simplicity and interpretability of the REHA algorithm hold promise for uncovering significant protein relations indicative of both the effect of exercise and different glycemic conditions. We anticipate that this approach will enable the discovery of new alternative pathways, offering fresh perspectives in understanding complex protein interactions involved in the response to exercise in IR and T2D subjects. This study aims not only to enrich our current knowledge base but also to open avenues for future research and potential therapeutic interventions.

## Results

**Figure 1:**
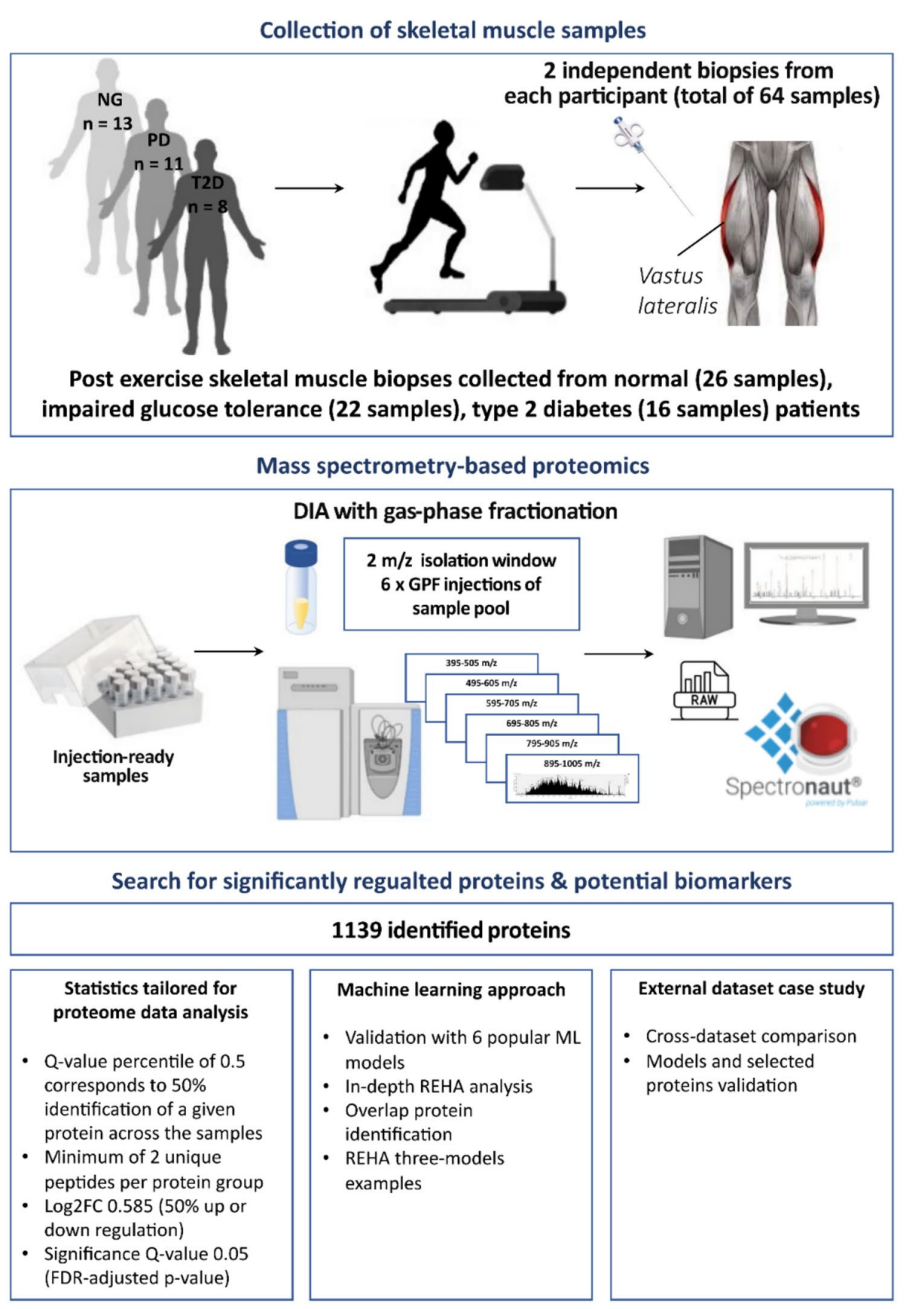
Overview of the experimental and analytical workflow employed for the identification of protein relations in post-exercise skeletal muscle biopsies from normal, impaired glucose tolerance, and type 2 diabetic cohorts, showing the steps from data collection to machine learning analysis with REHA, statistical significance testing, and external dataset validation.

The aim of this study was to identify complex protein associations that could shed light on the underlying mechanisms of insulin resistance, pathogenesis of type 2 diabetes (T2D) in subjects subjected to mixed-mode exercise. Figure 1 presents the comprehensive workflow of our experiments, from the collection and preprocessing of post-exercise skeletal muscle biopsies in three cohorts to the resulting analyses. The process began with rigorous data quality control (QC), transformation, and filtering to pinpoint 1139 proteins. Subsequently, three principal investigative segments: proteomics-tailored analytical pipeline, machine learning methods, and external dataset validation; were pursued in parallel, each contributing to a multifaceted understanding of the proteomic landscape in relation to glucose tolerance and diabetes.

### Machine learning approach

In the context of our machine learning exploration, REHA was a key component, providing a clear and intuitive model that differed from proteomics-tailored statistical pipeline standard methods. By adopting this innovative method, we could explore the dataset deeply, identifying protein patterns across varying levels of glucose tolerance.

Our analysis focused on binary classification tasks to identify unique patterns in protein expression distinguishing NG-PD (normal glucose tolerance to prediabetes), NG-T2D (normal glucose tolerance to type 2 diabetes), and T2D-PD (type 2 diabetes to prediabetes) subjects which underwent physical exercise regime. REHA was instrumental in crafting predictive models and uncovering the complex interactions of proteins within these groups. To ensure a comprehensive analysis, we assessed REHA’s effectiveness with various established machine learning algorithms, supported by thorough statistical evaluation.

The results provided by REHA and a comparative evaluation with other algorithms is presented in Table 1. It details the performance metrics of the predictions from the tenfold cross-validation process. The analysis reveals that REHA can effectively distinguish NG from PD, suggesting that specific protein interactions change with glucose tolerance. However, differentiating between T2D and PD was more challenging, with REHA’s results displaying greater variability in this comparison. When comparing REHA’s results with those of other white-box classifiers, its effectiveness is clearly visible. The use of statistical tests, particularly the Friedman test followed by Dunn’s multiple comparison post-hoc test^14^ at a 0.05 significance level, showed that REHA outperformed popular interpretable classifiers like J48, CART, and JRip in terms of accuracy, making these differences noteworthy. Conversely, Naive Bayes (NB) and black-box classifiers (such as random forest (RF) and Support Vector Machine (SVM)), showed enhanced accuracy, which was anticipated given their more complex designs. Additional information including ROC curves of REHA algorithm are included in Appendix J.

**Table 1:** Performance comparison of various machine learning algorithms.

Dataset\results		NG-PD				NG-T2D				T2D-PD			
		ACC	AUC	F1	WF1	ACC	AUC	F1	WF1	ACC	AUC	F1	WF1
REHA	Value	0.88	0.85	0.86	0.85	0.91	0.87	0.88	0.88	0.76	0.74	0.76	0.76
	Stdev	0.07	0.05	0.04	0.03	0.08	0.08	0.06	0.05	0.08	0.10	0.06	0.06
J48	Value	0.73	0.73	0.74	0.74	0.69	0.66	0.75	0.69	0.49	0.48	0.54	0.45
	Stdev	0.22	0.23	0.22	0.23	0.13	0.16	0.14	0.13	0.20	0.22	0.25	0.22
CART	Value	0.76	0.75	0.79	0.78	0.67	0.64	0.73	0.69	0.53	0.47	0.62	0.44
	Stdev	0.23	0.24	0.22	0.22	0.16	0.18	0.14	0.14	0.18	0.17	0.22	0.24
JRip	Value	0.73	0.73	0.77	0.74	0.71	0.69	0.76	0.70	0.57	0.54	0.63	0.55
	Stdev	0.19	0.20	0.16	0.19	0.20	0.23	0.19	0.21	0.23	0.24	0.25	0.27
RF	Value	0.86	0.95	0.86	0.86	0.97	0.99	0.98	0.96	0.81	0.89	0.86	0.83
	Stdev	0.15	0.11	0.17	0.16	0.08	0.02	0.06	0.09	0.19	0.17	0.15	0.20
SVM	Value	0.98	0.98	0.97	0.98	1.00	1.00	1.00	1.00	0.92	0.91	0.92	0.92
	Stdev	0.07	0.07	0.09	0.07	0.00	0.00	0.00	0.00	0.15	0.16	0.16	0.15
NB	Value	0.80	0.82	0.81	0.8	0.92	0.91	0.94	0.91	0.87	0.89	0.89	0.88
	Stdev	0.17	0.19	0.17	0.16	0.12	0.15	0.09	0.12	0.19	0.20	0.16	0.19

This table presents the averaged tenfold cross-validation results for accuracy (ACC), area under the ROC curve (AUC), F1-score (F1), and weighted F1-score (WF1)

*REHA* relative evolutionary hierarchical analysis, *J48—C4.5* algorithm-based classification and decision tree, *CART* Classification and regression decision tree, *RF* random forest algorithm, *JRip* Repeated incremental pruning rule learner, *SVM* support vector machine; *NB* Naive Bayes.

**Figure 2:**
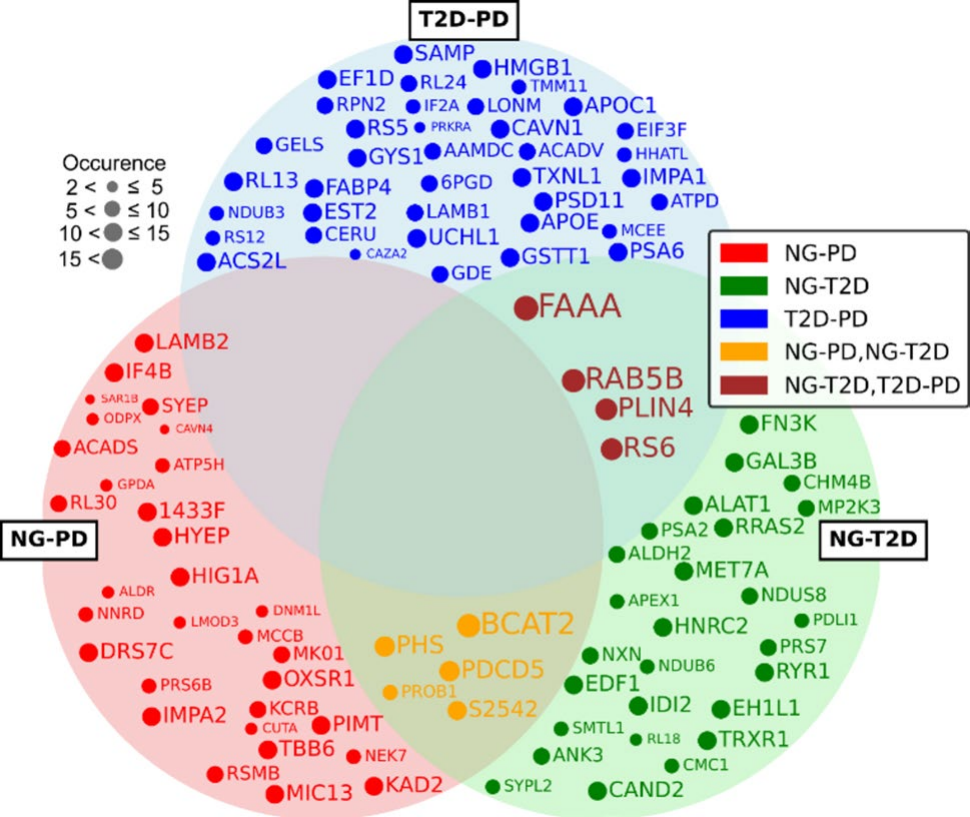
Proteins identified by REHA as significant for classification tasks between NG-PD, NG-T2D, and T2D-PD. Each circle represents a set of proteins relevant to a specific classification problem, with overlaps indicating proteins of importance to multiple conditions. The size of each dot within the circles correlates to the frequency of a protein's selection in the classification models, highlighting its relative importance.

In order to understand the molecular intricacies of glycemic variations, we leveraged the REHA algorithm to highlight key proteins that may serve as biomarkers across different states of glucose tolerance. The identification of these proteins is crucial, as it helps in understanding common pathological pathways and potential targets for therapeutic intervention. To visually show the interaction and significance of these proteins, we present a Venn diagram in Fig. 2 that clearly outlines the unique and shared proteins involved in each classification task. Each protein description together with its importance is described in Appendix C and D.

**Figure 3:**
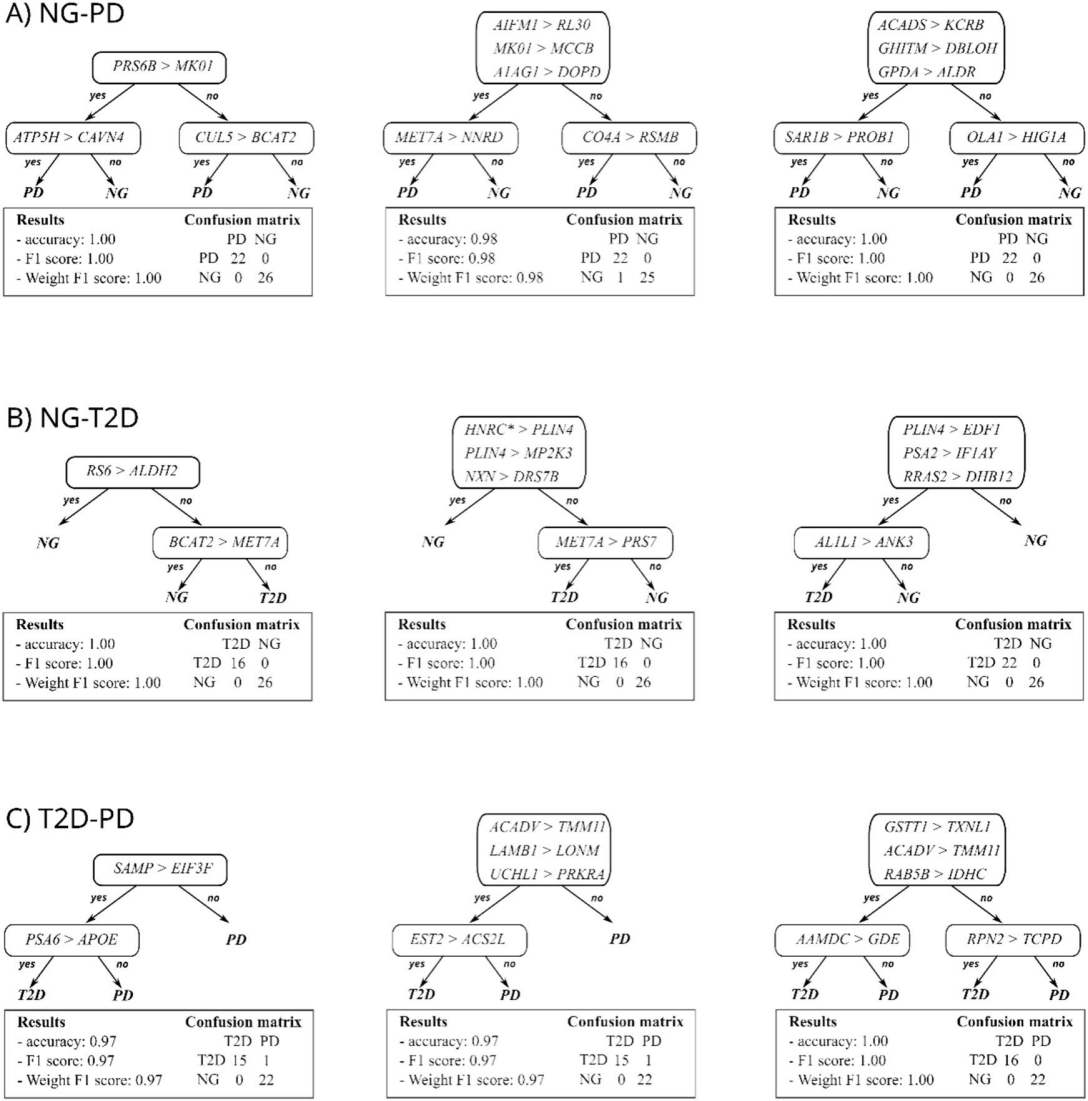
Representative REHA-generated classification trees for the: (**A**) NG-PD; (**B**) NG-T2D and (**C**) T2D-PD problem. These models illustrate one of the decision paths and highlight key protein interactions. Below each tree, the model's predictive performance and confusion matrix are provided for evaluation.

In the varied context of our machine learning analysis, REHA’s robustness is demonstrated by its ability to generate straightforward yet effective rules for protein interaction. Figure 3 provide visual examples of this, where the most commonly generated REHA models are presented for each classification challenge. These decision trees provide insight into the majority relationships between pairs of proteins at each node, offering a detailed view of the decision-making process of the algorithm. Below each tree, a set of statistics is provided, which includes a confusion matrix, underscoring the predictive success and reliability of the models.

From Fig. 3, we see that the classification trees generated by REHA are straightforward and easily interpretable. Each test at the internal node examines a simple ordering relationship within the samples, directly derived from the concept of Relative eXpression Analysis (RXA). This approach ensures that we do not fall into the pitfalls of overfitting and remain more resilient to data inconsistencies since we are not focused on the actual expression values of the proteins.

Furthermore, it is observable that some splits involve more than one comparison (test). Such a scenario indicates that the node was constructed using a multi-test concept^11^. This complex test can encompass several simple RXA comparisons that partition the data in a similar manner. The REHA algorithm typically employs this strategy at the root of the tree to enhance the stability of decisions and to reduce the risk of underfitting. In such tests, all the individual tests cast votes (each with equal weight) and collectively determine the data split outcome at the node.

The trees presented in Fig. 3 are further elaborated upon in the discussion section and each one is described in detail in Appendix B with proteins description in Appendix C and D. However, it is important to consider that REHA uses an evolutionary algorithm^15^, which means each run may yield a different decision model. The examples showcased here represent some of the most recurrent models or their variants produced by multiple runs of the algorithm.

### Statistics tailored for proteome data analysis

In our study, we opted to employ a proteomics-specific statistical approach to analyze our data, recognizing the limitations of conventional statistical methods for our complex proteomic dataset. Traditional methods, such as the Wilcoxon signed-rank test or the t-test, adjusted with Bonferroni correction for multiple comparisons, often fall short in accommodating the intricacies of proteomic data, which include high dimensionality and variable data completeness across a large set of observations. These foundational biostatistical analyses identified only a limited number of proteins as significantly different across conditions: five for NG-PD, one for T2D-PD, and 42 for NG-T2D, as detailed in Appendix E.

Given these constraints, our study employs a more tailored approach better suited to address the specific challenges of proteomic data. This includes criteria such as protein presence in 50% of the samples, requiring at least two unique peptides per protein, and a threshold for differential expression set at 50% of up or down-regulation. Additionally, we applied multiple testing corrections through False Discovery Rate (FDR) control to correct differential expression *p*-value set at < 0.05 cutoff.

This proteomics-specific approach led to the identification of 207 significantly different proteins in T2D-NG, 98 in PD-NG, and 79 in T2D-PD comparison. It also revealed overlaps that suggest shared pathological mechanisms across these conditions. Appendix F provides a comprehensive list of all proteins identified through our proteomics-specific statistical approach. This appendix ensures that readers interested in exploring the full breadth of our data can access this detailed information.

**Figure 4:**
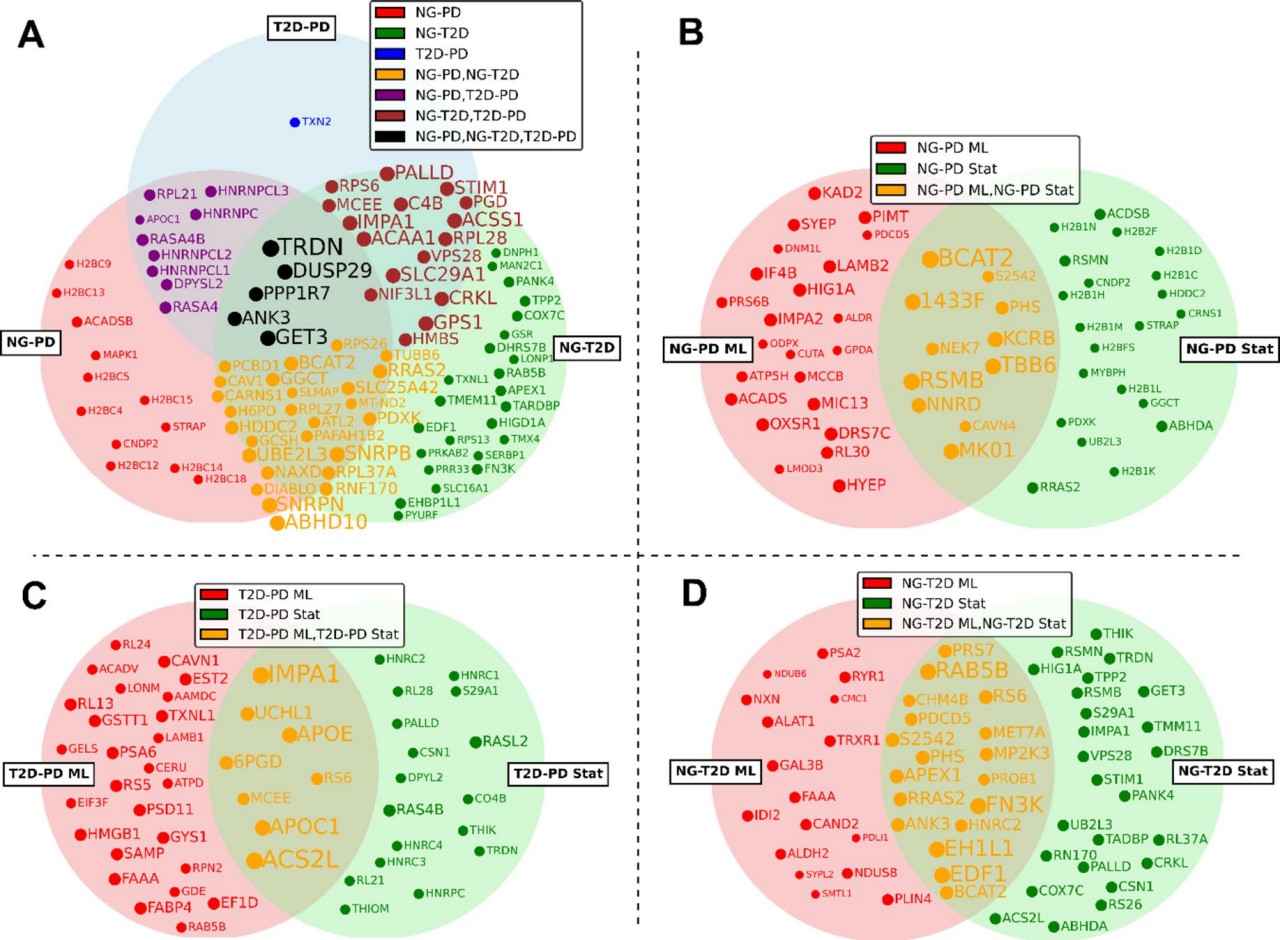
Venn diagrams showing proteins identified as significant by: (**A**) the proteomics-specific statistics for NG-PD, NG-T2D, and T2D-PD challenges; (**B**,**C**,**D**): proteins identified as significant by both machine learning (ML) and proteomics-specific statistics (Stat) for NG-PD, T2D-PD and NG-T2D respectively.

To manage the extensive data and focus on the most significant findings, the top 50% of proteins with the highest *p*-values for each class are shown in Fig. 4A, provided they exist in at least two classes. For proteins unique to one class, only the top 25% are displayed. This selective visualization allows us to draw attention to the most impactful proteins while maintaining clarity in our presentation.

Furthermore, to illustrate the integration of machine learning findings with our statistical results, we include three separate Venn diagrams (one for each challenge) in Fig. 4B–D. These diagrams display the proteins found significant by the machine learning approach (REHA), by the proteomics-specific statistical approach, and those that overlap, thereby emphasizing the synergy between computational modeling and empirical data analysis in understanding complex biological systems. This comparative visualization helps to validate the effectiveness of the REHA model and highlights the complementary nature of machine learning and statistical approaches in identifying key proteins relevant to the progression of glucose intolerance to diabetes.

### External dataset case study

The robustness of the REHA models and their comparative performance alongside other machine learning methodologies were tested in the third part of our study. We utilized an external dataset from a proteomic analysis of skeletal muscle^16^, which mirrored our classification groups: NG, PD, and T2D. To facilitate a coherent integration, we merged this external data with our own, focusing on 645 shared proteins and employing normalization procedures. The last step, while not necessary for the REHA algorithm due to its reliance on order relations, proved essential for ensuring compatibility with other machine learning algorithms.

Upon combining the datasets, a noticeable decline in REHA's performance on the merged dataset was observed, a trend that was mirrored by competing algorithms, suggesting a common challenge posed by the integration of external data. Table 2 aggregates the predictive performance statistics of these algorithms, revealing variations across different metrics such as: accuracy (ACC), area under the ROC curve (AUC), F1 score, and Weighted F1 score (WF1), all averaged over tenfold cross-validation. Additional information including ROC curves of REHA algorithm are included in Appendix J.

**Table 2:** Performance comparison of various machine learning algorithms on merged datasets.

Dataset\results		NG-PD merge				NG-T2D merge				T2D-PD merge			
		ACC	AUC	F1	WF1	ACC	AUC	F1	WF1	ACC	AUC	F1	WF1
REHA	Value	0.68	0.70	0.61	0.68	0.67	0.68	0.55	0.68	0.70	0.66	0.74	0.70
	Stdev	0.08	0.10	0.03	0.03	0.07	0.11	0.03	0.03	0.07	0.09	0.03	0.02
J48	Value	0.57	0.58	0.62	0.57	0.79	0.79	0.82	0.78	0.57	0.58	0.49	0.55
	Stdev	0.17	0.19	0.16	0.17	0.16	0.16	0.15	0.16	0.21	0.22	0.27	0.22
CART	Value	0.59	0.62	0.66	0.6	0.65	0.65	0.71	0.65	0.53	0.49	0.4	0.45
	Stdev	0.16	0.19	0.18	0.19	0.15	0.17	0.15	0.17	0.12	0.12	0.24	0.18
JR	Value	0.55	0.54	0.59	0.53	0.61	0.6	0.67	0.61	0.49	0.48	0.39	0.46
	Stdev	0.16	0.17	0.18	0.16	0.17	0.18	0.17	0.19	0.18	0.19	0.25	0.19
RF	Value	0.66	0.71	0.71	0.66	0.79	0.86	0.84	0.78	0.68	0.77	0.58	0.68
	Stdev	0.17	0.22	0.17	0.18	0.14	0.14	0.11	0.16	0.17	0.20	0.27	0.19
SVM	Value	0.80	0.78	0.85	0.82	0.83	0.78	0.88	0.83	0.67	0.66	0.63	0.67
	Stdev	0.13	0.15	0.1	0.12	0.11	0.14	0.07	0.12	0.14	0.14	0.18	0.15
NB	Value	0.53	0.54	0.54	0.52	0.57	0.61	0.61	0.56	0.55	0.67	0.48	0.54
	Stdev	0.22	0.24	0.23	0.23	0.22	0.23	0.22	0.23	0.18	0.20	0.25	0.19

This table presents the averaged tenfold cross-validation results for: accuracy (ACC), area under the ROC curve (AUC), F1-Score (F1), and Weighted F1-Score (WF1).

Despite the inherent challenges of dataset merging and a reduced feature set, REHA's performance, a white-box model, remained robust, albeit with a decline from its performance on the unmerged dataset. The other white-box models, in contrast, experienced a more significant performance decrease across all comparisons. This underscores the challenges these models face in adapting to datasets with fewer features, which may limit their ability to perform fine-grained data separations based on explicit decision rules. Similarly, the NB algorithm struggled with the merged data, indicating difficulties in accommodating newly integrated data points.

Meanwhile, black-box models, known for their complex, non-linear decision-making capabilities, also saw a performance drop post-merge. However, the SVM algorithm and, to some extent, the RF algorithm maintained relatively high-performance metrics, reflecting their resilience to the challenges posed by data merging, which often arise from disparities in feature representation and integration-related noise.

**Figure 5:**
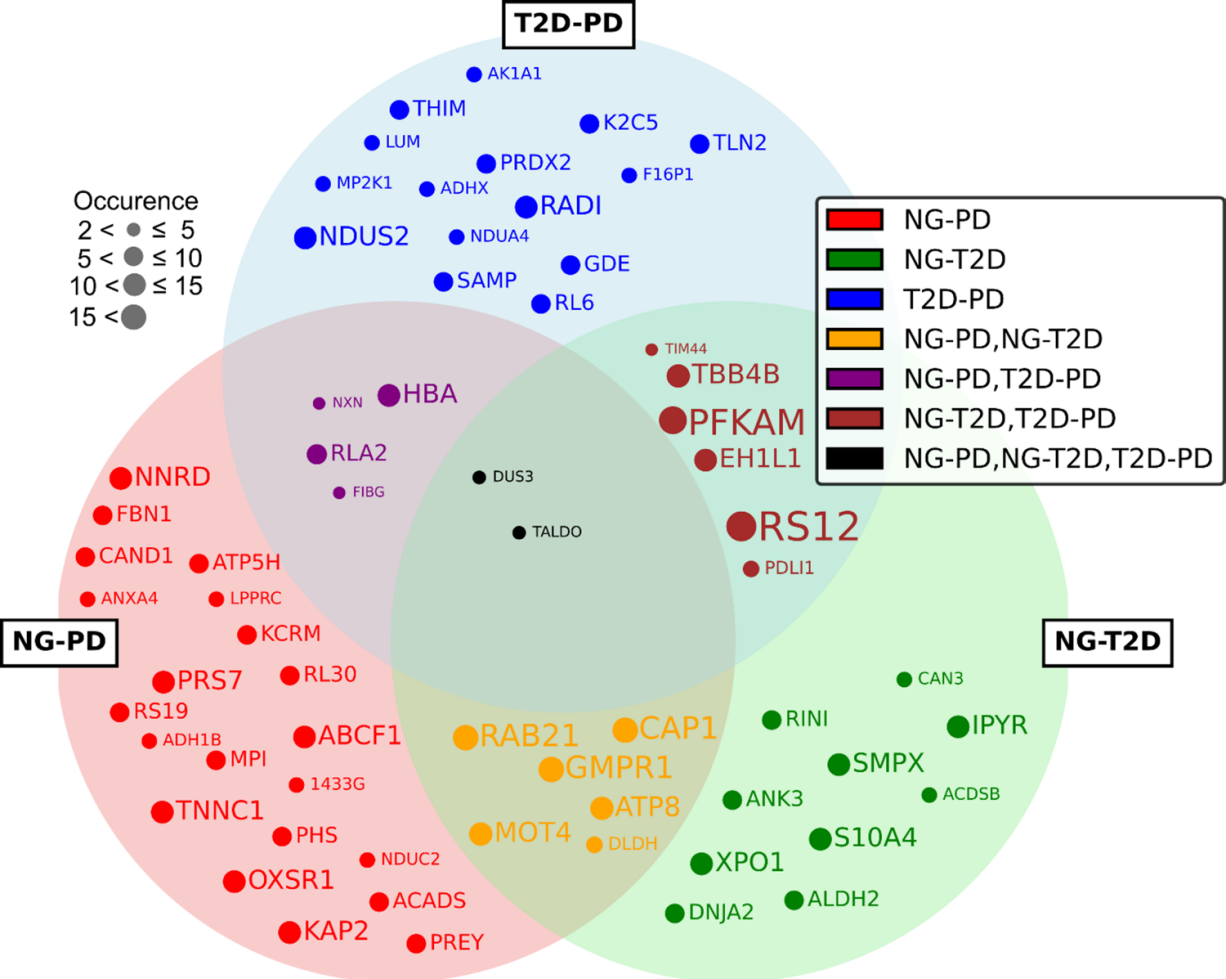
Proteins identified by REHA as significant for classification tasks between merged NG-PD, NG-T2D, and T2D-PD datasets. Each circle represents a set of proteins relevant to a specific classification problem, with overlaps indicating proteins of importance to multiple conditions.

We have conducted a thorough comparison between the protein selections used in the REHA prediction models for the original datasets and those identified after merging. The Venn diagrams developed from this data, illustrated in Fig. 5, offer visual insight into the overlap of protein selection across different glycemic states: NG-PD, NG-T2D, and T2D-PD. Intriguingly, our analysis revealed that despite the consolidation of datasets, which inherently entails a reduction in the number of features available for selection, there is a notable retention of key proteins across the datasets. Specifically, we observed that approximately 15–20% of the proteins, contingent on the specific condition under investigation, consistently re-emerged in the merged dataset. Each protein description together with its importance is described in Appendix G.

Finally, the merged dataset served as a foundation from which we derived two additional, distinct datasets: one exclusively containing our internal data and the other solely comprising data from external studies; each restricted to the same set of 645 proteins. The strategic intent behind this partitioning was to enable an in-depth comparison of the decision-making processes employed by the REHA model. We sought to ascertain the consistency of the rules generated by REHA and to explore whether the model’s reasoning would transcend the particularities of the datasets. This methodical curation aimed to provide a clear understanding of the model's performance and the potential universal applicability of the identified biomarkers across diverse data origins.

**Figure 6:**
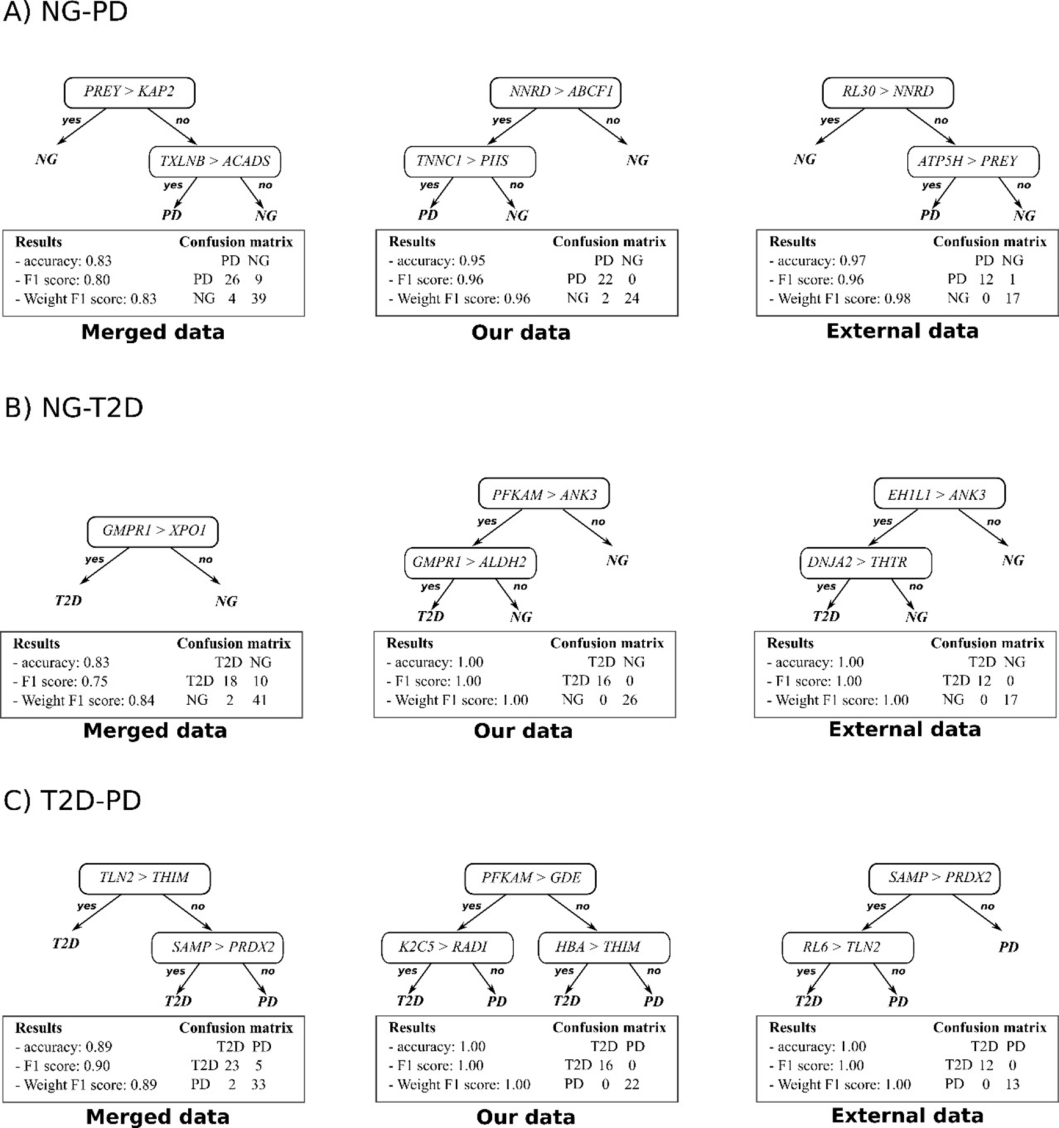
REHA-Generated Classification Trees for: (**A**) NG-PD; (**B**) NG-T2D; (**C**) T2D-PD. Figure displays merged, our, and external datasets limited to 645 proteins. Performance metrics and confusion matrices included.

In the NG-PD context (see Fig. 6A), the REHA model's decision tree constructed from the merged dataset utilizes biomarkers like PREY (mitochondrial protein preY, methyltransferase and chaperone involved in coenzyme Q biosynthesis) and KAP2 (cAMP-dependent protein kinase type II-alpha regulatory subunit, Regulatory subunit of cAMP-dependent protein kinases involved in cellular cAMP signaling), which remain significant even when the analysis is narrowed to our dataset restricted to the same 645 proteins. Interestingly, even the external data's tree, while operating under the same protein limitation, reveals a different but informative narrative, showcasing the REHA model's ability to adapt and identify relevant biomarker patterns regardless of the dataset.

Exploring the NG-T2D problem (see Fig. 6B), the merged dataset's decision tree presents proteins like NNRD and ABCF1 as possible biomarkers, with our dataset's analysis bringing PFKAM and ANK3 to the fore. The external dataset, again consistent in protein numbers, allows REHA to adapt its pattern recognition, suggesting a flexible but robust model capable of navigating through dataset-specific nuances.

For T2D-PD, proteins such as TLN2 and SAMP are identified as biomarkers within the merged dataset's tree, with these markers also proving pivotal in our dataset's analysis (see Fig. 6C). The model's interaction with the external dataset continues this theme, endorsing some biomarkers. Additional network diagram illustrating protein–protein interactions and comparisons, grouped based on the REHA machine learning approach for NG versus PD, NG versus T2D and T2D versus PD problems is included in Appendix I.

Although finding a universal rule applicable to all datasets was challenging due to the vast number of proteins in comparison to the dataset sizes, REHA has demonstrated its capacity for high-precision classification. The discerned patterns underscore the model’s ability to simplify complex data into actionable insights, offering a clear view of how even a restrained subset of proteins can be pivotal in distinguishing between intricate health states.

## Discussion

Proteomics poses specific challenges in data analysis and interpretation primarily due to the high-dimensional nature of the data and the intricate relationships between proteins. Therefore, adopting a more comprehensive approach is crucial for uncovering subtle yet significant meaningful differences in protein expression. It may involve employing the proteomic-tailored pipeline as well as application of some “white box” machine learning methods. This comprehensive dual strategy is invaluable for revealing novel patterns of changes and generating focused hypotheses.

Our exploratory analysis of protein expression in skeletal muscle across varying glycemic states after exercise challenge offers valuable preliminary insights into the metabolic complexities of IR and T2D. By integrating proteomic analysis with the REHA machine learning algorithm, we have identified interesting patterns and potential interactions in protein expression. Next, by using statistical methods dedicated to proteome data analysis, we found a total of 250 unique proteins as statistically significant between experimental groups. Among these, the expression of 98 proteins were significantly affected in the PD-NG- comparison, 207 in T2D-NG, and 79 in T2D-PD.

**Figure 7:**
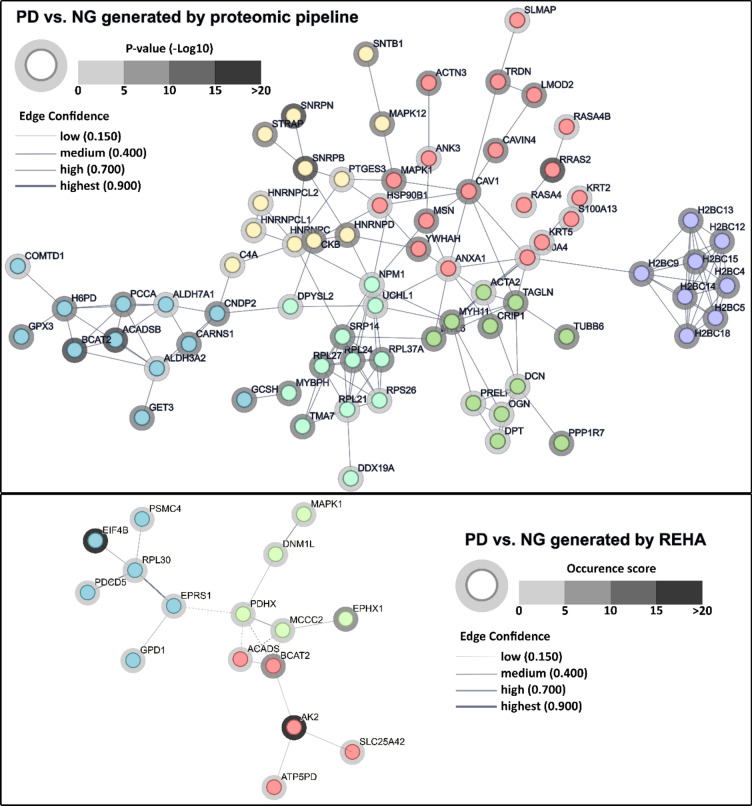
Network diagram illustrating the genes associated with the proteins identified in Fig. 4 by proteomic-tailored pipeline (top) and REHA (bottom), as generated by String software for PD versus NG comparison. Each node corresponds to a differentially regulated protein. Node halo shading depicts significance of protein differential expression from a given comparison. The lines (edges) connecting individual nodes correspond to functional and physical protein associations according to curated STRING network, whereas line thickness represents the confidence of their mutual interaction. Node color clusters proteins connected by strongest associations according to curated STRING database. Dashed lines depict inter-cluster physical and functional connections between particular proteins.

In the following discussion, we will examine the results of these dual analysis. Due to the length restrictions, we will focus here only on PD-NG comparison, while the rest of the discussion as well as the Functional Enrichment Analysis are enclosed in Appendix H and I in the supplementary materials. Figure 7 illustrates network diagrams generated using String software^17^ for proteins identified by REHA approach (top) and statistical approach dedicated to proteomics data that are shown in Fig. 4.

### Prediabetes versus normoglycemic state—proteomic pipeline

In the context of comparing PD to NG using proteomic-tailored pipeline, the network analysis leads to a distinction between two main clusters of interconnected proteins and identifies several smaller ones (see Fig. 7).

In the center of the Red Cluster, there are Caveolin-1 (CAV1) and Caveolae-associated protein 4 (CAVIN4, CAVIN4 gene). Both are involved in the formation and function of caveolae, which are 50–100 nm sized invaginations in the cell membranes crucial for multiple cellular functions such as endocytosis, cholesterol homeostasis, signal transduction, and mechano-protection^18,19^. These structures have been shown to play a role in insulin receptor-mediated signaling and GLUT4 trafficking in skeletal muscle, with impaired function potentially leading to insulin resistance. Yoon Sin Oh et al. found that the expression of caveolins, particularly caveolin-1, along with insulin-related proteins, was increased by resistance exercise training in the skeletal muscle of 50-week-old Sprague Dawley rats. The upregulation of caveolin-1 was specifically associated with improved insulin sensitivity, especially in type 2 (EDL) muscle fibers. This suggests that the increase in the expression of caveolin-1 in skeletal muscle is dependent on the type of muscle fiber and the type of exercise performed, which may be important for improving insulin sensitivity in skeletal muscle^18^. The downregulation of caveolin-1 in the prediabetes group compared to normoglycemic individuals in our study could imply insufficient exercise among our patients and a possible transition from normoglycemia to a prediabetic state, which could impair caveolae function and insulin signaling.

Cavin-4 is predominantly expressed in cardiac and skeletal muscle. It regulates the morphology of caveolae and recruits MAPK1/3 to caveolae in cardiomyocytes. CAVIN4 activates MAPK1/3, regulating alpha-1 adrenergic receptor-induced hypertrophic responses, and contributes to the proper membrane localization and stabilization of caveolin-3 (CAV3) in cardiomyocytes. The distribution of Cavin-4 is disrupted in human muscle diseases related to Caveolin-3 dysfunction^20^. The lower expression of Cavin-4 in our study might indicate a compromised caveolae function, which could be contributing to impaired insulin signaling and glucose uptake in prediabetic patients.

Another important interconnection within this cluster involves Mitogen-activated protein kinase 1 (MK01, MAPK1 gene), which is downregulated in our study in the prediabetes group compared to normoglycemic individuals. MAPK signal transduction pathway proteins control cellular functions such as growth, differentiation, apoptosis, and proliferation^21,22^.

Furthermore, within the Red Cluster, there are members of the Ras GTPase protein family: Ras-related protein R-Ras2 (RRAS2), Ras GTPase-activating protein 4 (RASL2, RASA4 gene), and Ras GTPase-activating protein 4B (RAS4B, RASA4B gene), involved in regulating various cellular processes, such as the MAPK signaling pathway. Inhibition of Ras signaling has been shown to improve insulin sensitivity and glucose uptake both in vitro and in vivo. Beneficial effects of Ras inhibition have been observed, leading to attenuation of hyperglycemia in conventional models of type 2 diabetes^23^. In our study, we found lower expression of these proteins in the prediabetes group compared to normoglycemic individuals. This observation is not consistent with the expected trend, as reduced Ras signaling typically correlates with improved insulin sensitivity and glucose uptake, which might suggest an atypical response or compensatory mechanism in the initial stages of metabolic dysfunction.

Additionally, our study has shown lower expression of a member of the tail-anchored membrane proteins superfamily, Sarcolemmal membrane-associated protein (SLMAP), in the prediabetes group compared to normoglycemic individuals. SLMAP is found in various locations within the cell, including the endoplasmic reticulum (ER), mitochondria, cell surface membranes, and the nuclear envelope. Studies have indicated that SLMAP participates in vital physiological processes, including ion channel regulation and membrane fusion, with increased expression associated with endothelial dysfunction in diabetes^24^. This discrepancy might indicate an early compensatory mechanism in prediabetes or a distinct regulatory pathway.

Other notable interactions include Protein S100-A13 (S10AD, S100A13 gene) and Protein S100-A4 (S10A4, S100A4 gene) of the S100 protein family, which exhibit various functions, including stress-induced release of growth factors and adipogenesis regulation, with potential implications for obesity and insulin resistance^25–27^. Moreover, in vitro findings suggest that S100A4 may have an anti-inflammatory impact^27^. Our results showing lower expression of these proteins in prediabetic patients seem inconsistent with other studies that recognize S100A4 as an adipokine linked to insulin resistance (IR) and white adipose tissue (WAT) dysfunction in prepubertal populations, where variations in plasma S100A4 levels accompany longitudinal trajectories of IR during pubertal development in children^26^. On the other hand, Taxerås et al. showed that increased levels of S100A4 signify IR in adults with obesity but not in prepubertal children^27^.

Moesin (MOES, MSN gene) acts as a crucial link between the actin cytoskeleton and the plasma membrane, regulating specific cellular regions. It is consistently expressed in endothelial cells and is situated downstream of pathways mediating advanced glycation end products (AGEs). AGEs-induced moesin phosphorylation has been shown to be associated with subsequent hyperpermeability and endothelial cell barrier dysfunction in diabetes^28^.

Annexin A1 (ANXA1) plays roles in the inflammatory response and immune system modulation. It is found in vasculature, specifically in endothelial cells and vascular smooth muscle cells. Patients with T2D exhibited increased levels of ANXA1. Additionally, research on ANXA1-deficient mice showed that the absence of this protein exacerbates the diabetic phenotype, including dyslipidemia and insulin resistance, indicating a crucial role for ANXA1 in regulating these processes. Endogenous ANXA1 influenced the passive mechanics of the mesenteric artery in the context of insulin resistance, with its absence worsening the pathological remodeling of blood vessels in insulin-resistant mice^29^. ANXA1 has a dynamic role in regulating the metabolic state. At the prediabetes stage, its lower expression could be an early indicator of metabolic disturbances.

Proteins related to muscle structure and function highlight the network's complexity. Skeletal muscle fiber transformations take place in various biological processes, such as changes in neuromuscular activity, muscular disorders, age-related muscle loss, and during myogenesis^30^. We have shown higher expression of Alpha-actinin-3 (ACTN3), which is associated with muscle function^31^. The sarcomeric α-actinin proteins, actinin-2 and -3, are crucial components of the Z-line structure in skeletal muscles. They were previously believed to provide mechanical support during muscle contraction. However, recent research has revealed that these proteins are key adaptor proteins that interact with a range of structural, signaling, and metabolic proteins, including the key metabolic regulator glycogen phosphorylase (GPh)^31^. This absence of α-actinin-3 is associated with reduced muscle strength and mass, as well as a shift towards a more efficient oxidative metabolism. Therefore, deficiency in α-actinin-3 can negatively impact athletic performance^31,32^.

Additionally, in a small cohort of T2D patients, an increase in the number of individuals with α-actin-3 deficiency was noted, regardless of their BMI^32,33^. The study investigating the association between α-actinin-3 deficiency and obesity in both mice and humans showed mixed results regarding the role of ACTN3 R577X in weight gain and obesity among individuals of European descent. While the Actn3 KO 129X1/SvJ mice gained less weight compared to the wild-type (WT) mice when fed a high-fat diet (HFD) comprising 45% of calories from fat, the study found no significant contribution of the ACTN3 genotype alone to BMI or obesity in humans across six independent cohorts. However, this does not exclude the possibility of ACTN3 R577X playing a role in conditions linked to obesity, such as T2D and chronic heart failure (CHF)^32^.

Triadin (TRDN) regulates lumenal Ca2+ release from the sarcoplasmic reticulum through RYR1 and RYR2 channels, an essential step in triggering skeletal and heart muscle contraction. It ensures proper triad junction organization and is necessary for normal skeletal muscle strength.

Leiomodin is an actin filament nucleator in muscle cells related to tropomodulin, a capping protein localized at the pointed end of the thin filaments^34,35^. The role of leiomodin is crucial for the assembly and maintenance of thin filaments^34,36^. We have found lower expression of Leiomodin-2 (LMOD2) in prediabetic patients compared to healthy individuals, both after exercise intervention. Similarly, the expression of Leiomodin-1 (LMOD1) is typically reduced following either aerobic or resistance training. However, its role in skeletal muscle adaptations after exercise training is currently unclear^34,37^.

Additionally, we have identified a decrease in the expression of Ankyrin-3 (ANK3) in the prediabetes group compared to normoglycemic individuals. Ankyrins are adaptor molecules found in eukaryotic cells. They serve as crucial scaffolding proteins involved in anchoring to the muscle membrane, contributing to muscle development, neurogenesis, and synapse formation^38^. Functional variants of ankyrin-B have been linked to certain human diseases, including T2D^39^. Lorenzo et al. demonstrated that ankyrin-B (AnkB) deficiency in adipose tissue (AT) causes cell-autonomous adiposity, rendering mice more susceptible to becoming obese with age or when fed a high-fat diet, and causing other metabolic complications^40^. Our findings of decreased ANK3 expression in prediabetic patients could be consistent with ankyrins' involvement in metabolic regulation and disorders. Therefore, it will be important to understand the individual metabolic roles of ankyrins in other tissues, along with the resulting inherent effects of their deficiencies and how they affect metabolic organ cross-talk.

Additionally, this cluster includes Heat shock protein 90B1 HSP90B1/ENPL Endoplasmin. Heat shock proteins (HSPs) are known as chaperones and have roles in cell signaling and regulation of metabolism. In patients with diabetes, the expression and levels of HSPs decline as we shown in our study; however, these chaperones can help alleviate some diabetic complications, including oxidative stress and inflammation^41^.

This cluster mainly comprises proteins associated with cellular structure, signaling, and stress responses, reflecting key adaptive changes in prediabetes conditions affecting various cellular pathways.

Within the Yellow Cluster notable members include Complement C4-A (CO4A, C4A gene), a part of the classical complement pathway important for immune response and linked to the development of diabetes mellitus^42^. The upregulation of Complement C4-A in prediabetic individuals may align with the findings of D. E. McMillan, showing that serum levels of both C4 and C3 are increased in patients with diabetes^43^.

Creatine kinase B-type (KCRB, CKB gene) plays a significant role in ATP formation, which is necessary for energy-demanding processes in human and animal cells, especially muscle contractions^44^. The observed lower expression in prediabetes group could indicate a reduced capacity for efficient ATP production in muscle cells, potentially contributing to insulin resistance and other metabolic complications. However, studies in both animals and humans have shown that Creatine kinase is associated with insulin resistance and has been identified as a risk marker for cardiovascular disease, primarily due to its relationship with hypertension and increased body mass index^45^. It was also correlated with glycated haemoglobin in a nondiabetic general population^46^.

Heterogeneous nuclear ribonucleoproteins C-like 1, 2 ,3, 4, C1/C2 (HNRNPCL1, HNRNPCL2, HNRNPCL3, HNRNPCL1, HNRNPC genes) are involved in RNA processing^47^ and may potentially impact on insulin sensitivity and glucose metabolism^48^. We demonstrated the downregulation of these proteins in the prediabetes group participating in our study, which is consistent with the studies performed by Zhao M et al. They observed that reduced expression of hnRNP A1 in skeletal muscle affects metabolic properties and systemic insulin sensitivity by inhibiting glycogen synthesis, further supporting the crucial role of hnRNPs in maintaining metabolic homeostasis^48^.

Additionally, Serine-threonine kinase receptor-associated protein (STRAP) and Small nuclear ribonucleoprotein-associated proteins B and B' (RSMB, SNRPB gene) and N (RSMN, SNRPN gene) are involved in RNA splicing processes and may be related to various diseases^49,50^.

This cluster emphasizes features proteins crucial for immune response, ATP formation, insulin sensitivity, and RNA processing. These proteins may contribute to conditions like IR and T2D, indicating their significance in disease pathways.

Short/branched-chain specific acyl-CoA dehydrogenase, mitochondrial ACADS (ACADS gene) is one of the acyl-CoA dehydrogenases involved in the catabolism of short branched-chain acyl-CoA derivatives. It emerges as a pivotal node within the smaller Blue Cluster, demonstrating a regulatory effect on the metabolism of free fatty acids (FFAs), triglycerides (TGs), and cholesterol (CHOL)^51^. The lower expression of this enzyme observed in prediabetes could impair the breakdown of L-isoleucine and contribute to disruptions in lipid metabolism, thereby affecting overall metabolic homeostasis.

Other interconnected proteins in this cluster include Branched-chain-amino-acid aminotransferase, mitochondrial (BCAT2), which catalyzes the first reaction in the catabolism of the crucial branched-chain amino acids (BCAAs): leucine, isoleucine, and valine. BCAAs and related metabolites are now widely recognized as being strong biomarkers of obesity, insulin resistance, type 2 diabetes (T2D), and cardiovascular diseases in humans^51^. The skeletal muscle is a major site of BCAA catabolism, with BCAT2 being predominantly expressed in this tissue^52^.

The lower expression of BCAT2 in the prediabetes group compared to normoglycemic individuals, as shown in our studies, suggests decreased activity of branched-chain-amino-acid aminotransferase (BCAT) in muscle. Impairments in BCAAs metabolism are linked to insulin resistance and T2D by the accumulation of possibly toxic intermediates and BCAAs levels in plasma^53,54^.

This aligns with findings observed in BCAT2-/- mice, which also exhibit reduced levels of BCAA metabolites in peripheral tissues^55^. Despite continuous activation of mTORC1, BCAT2-/- mice do not develop insulin resistance; instead, they show improved glycemic control and insulin sensitivity with high energy expenditure^53^.

Additionally, we have shown downregulation of Alpha-aminoadipic semialdehyde dehydrogenase (ALDH7A1, ALDH7A1 gene), which is pivotal in lysine catabolism; Propionyl-CoA carboxylase alpha chain, mitochondrial (PCCA), which is essential for the catabolism of odd-chain fatty acids, branched-chain amino acids (isoleucine, threonine, methionine, valine), and other metabolites; and Aldehyde dehydrogenase family 3 member A2 (ALDH3A2, ALDH3A2 gene), which oxidizes medium to long-chain aliphatic aldehydes into fatty acids. This pathway, an alternative to glycolysis, provides essential reducing power and metabolic intermediates for biosynthetic processes. Deficiencies in these enzymes' activity may affect the metabolic pathways they engage in, leading to an accumulation of their respective substrates and potentially toxic intermediates.

The cluster also includes: Endoplasmic bifunctional protein GDH/6PGL (G6PE, H6PD gene), which operates within the endoplasmic reticulum, facilitating the initial steps of the pentose phosphate pathway and ATPase GET3 (GET3 gene), which is crucial for post-translational transportation of tail-anchored (TA) proteins to the endoplasmic reticulum (ER). There is also Cytosolic non-specific dipeptidase (CNDP2, CNDP2 gene), an enzyme that primarily breaks down Cys-Gly dipeptides but can hydrolyze various other dipeptides, including L-carnosine. It may function as a tumor suppressor, as elevated CNDP2 levels have been shown to induce cell apoptosis, while lower levels promote cell proliferation. Our study also revealed lower expression of catechol O-methyltransferase domain-containing protein 1 (CMTD1, COMTD1 gene). The genetic variation in catechol-O-methyltransferase (COMT), an enzyme that degrades catecholamines, is linked to cardiometabolic risk factors and incident cardiovascular disease (CVD). The COMT rs4680 high-activity G-allele was associated with lower HbA1c levels and modest protection from type 2 diabetes^56^.

Additionally, Carnosine synthase 1 (CRNS1, CARNS1 gene), vital for synthesizing carnosine and homocarnosine, is present. Carnosine is the most common naturally occurring histidyl dipeptide present in human skeletal muscle with a number of functions, including buffering pH levels and scavenging by-products of lipid peroxidation, as well as quenching reactive oxygen species and chelating first transition metals^57–59^. Studies have shown that exercise can increase carnosine levels, while levels of this dipeptide are significantly reduced in the muscles of both normal humans and T2D patients^57,60,61^. Moreover, carnosine scavenging of glucolipotoxic free radicals has been shown to have beneficial effects on glucose homeostasis by increasing insulin secretion and skeletal muscle glucose uptake^59^. Thus, our findings showing lower expression of Carnosine synthase 1 align with the broader trend, indicating that reduced expression of CARNS1 may lead to decreased carnosine levels, which can negatively impact glucose metabolism in prediabetic individuals.

Finally, we have shown downregulation of Glutathione peroxidase 3 (GPX3 gene) in the prediabetes group, which is vital in managing oxidative stress and enhancing antioxidant defense mechanisms. Our results are consistent with Chung et al.'s findings that GPx3 facilitates the antioxidant impact of peroxisome proliferator-activated receptor γ (PPARγ) in human skeletal muscle cells, suggesting that reduced GPx3 expression may impair antioxidant defenses and contribute to insulin resistance in prediabetic individuals^62^.

This cluster mainly comprises proteins involved in lipid metabolism, BCAA catabolism, and oxidative stress responses, reflecting significant metabolic alterations in prediabetes that impact insulin sensitivity and overall metabolic homeostasis.

The Green Cluster (on the left side) prominently features proteins crucial for protein synthesis. Among these are 60S ribosomal protein L21 (RL21, RPL21 gene), 60S ribosomal protein L24 (RL24, RPL24 gene), 60S ribosomal protein L27 (RL27, RPL27 gene), 60S ribosomal protein L37a (RL37A, RPL37A gene), and 40S ribosomal protein S26 (RS26, RPS26 gene). Additionally, nucleophosmin (NPM, NPM1 gene) participates in various cellular processes such as ribosome biogenesis, cell proliferation, and regulation of tumor suppressors, while Translation Machinery Associated 7 homolog (TMA7) is involved in translation regulation, and ATP-dependent RNA helicase DDX19A (DD19A, DDX19A gene) plays a role in mRNA export from the nucleus. Other notable proteins within this cluster include Signal Recognition Particle 14 kDa protein (SRP14), which participates in targeting secretory proteins to the rough endoplasmic reticulum membrane, as the ER is one of the key cellular structures that determines the maintenance of normal properties and functions of synthesized proteins.

The reduced expression of these proteins in muscle samples from prediabetic patients participating in our study is consistent with findings that indicate decreased protein synthesis. Adaptation to chronic insulin resistance may involve a reduction in ribosomal protein expression, leading to disruptions in muscle function^52^.

Furthermore, Ubiquitin Carboxyl-Terminal Hydrolase Isozyme L1 (UCHL1) is involved in processing ubiquitin precursors and ubiquitinated proteins, contributing to protein processing and axonal integrity^63^.

Lastly, Myosin-Binding Protein H (MYBPH) exhibits a strong affinity for myosin and regulates sarcomere assembly and stability. It interacts with thick myofilaments in the A-band of the sarcomere, playing a critical role in muscle contractile function and structure maintenance^64^.

This cluster primarily includes proteins essential for protein synthesis, cellular processes, and muscle function, highlighting significant adaptations in muscle metabolism and structure in prediabetic conditions.

On the right side we can distinguish the Lime Green Cluster with downregulated Myosin-11 (MYH11) and Myosin light polypeptide 6 (MYL6), both crucial in muscle contraction regulation. It also includes Tubulin beta-6 chain (TBB6, TUBB6 gene), a major constituent of microtubules. The modulation of a β-tubulin isotype is involved in muscle differentiation and regeneration^65^. Additionally, Decorin (PGS2, DCN gene) may influence the rate of fibril formation. Actin, aortic smooth muscle (ACTA, ACTA2 gene), in its intermediate form, contributes to various types of cell motility^66^. Cysteine-rich protein 1 (CRIP1) appears to be involved in zinc absorption and may function as an intracellular zinc transport protein. Dermatopontin (DERM, DPT gene) mediates adhesion via cell surface integrin binding and enhances TGFB1 activity. Mimecan (MIME, OGN gene) is a significant constituent of the skeletal muscle secretome, expressed differentially throughout muscle development^67^. Protein phosphatase 1 regulatory subunit 7 (PP1R7, PPP1R7 gene) acts as a regulatory subunit of protein phosphatase 1. Prolargin (PRELP) potentially anchors basement membranes to underlying connective tissue. Transgelin (TAGL, TAGLN gene) is an actin-binding cytoskeletal protein vital for maintaining muscle structure. Proteomic profiling of chronically low-frequency stimulated fast muscle has identified transgelin, along with cofilin-2, an endothelial marker and actin-binding protein, as novel biomarkers for evaluating muscle transformation^30^. Additionally, according to several studies, transgelin has been linked to increased cell motility and migration. Aldeiri et al. proposed that transgelin-expressing myofibroblasts play a primary role in mediating TGFβ signaling during ventricular septum (VBW) morphogenesis^68^.

This cluster consists of proteins involved in muscle differentiation, regeneration, and motility, reflecting significant disruptions in muscle structure and function in prediabetic conditions.

### Prediabetes versus normoglycemic state—machine learning approach

When comparing network diagrams derived from the PD versus NG analysis and grouped using the REHA machine learning approach, alongside statistical methods tailored for proteomic data, several common themes and distinct differences emerge. This highlights the unique contributions of both machine learning and traditional statistical methods in comprehending complex biological datasets.

The network analysis contrasting prediabetic (PD) and normoglycemic (NG) states grouped based on the REHA machine learning approach reveals three main/distinct clusters of proteins reflecting the complexity of metabolic processes (see Fig. 7).

The Red Cluster includes proteins such as Adenylate kinase 2, mitochondrial (KAD2, AK2 gene), crucial for cellular energy homeostasis, ATP synthase subunit d, mitochondrial (ATP5H, ATP5PD gene), which plays a role in skeletal muscle endocrine signaling and is associated with insulin resistance^69^, and Mitochondrial coenzyme A transporter SLC25A42 (S2542, SLC25A42 gene) mediating the transport of coenzyme A (CoA) in mitochondria in exchange for intramitochondrial (deoxy)adenine nucleotides and adenosine 3',5'-diphosphate. Furthermore, it incorporates previously described Short-chain specific acyl-CoA dehydrogenase, mitochondrial (ACADS), which regulates lipid metabolism and participates in L-isoleucine metabolism^51^, Branched-chain-amino-acid aminotransferase (BCAT2), vital for branched-chain amino acid catabolism, are present^70^.

Overall, it highlights proteins involved in mitochondrial functions and energy metabolism, potentially influencing cellular responses to prediabetic conditions.

The Green Cluster encompasses a diverse range of proteins with significant implications for various cellular processes and metabolic pathways. Among them is Dynamin-1-like protein (DNM1L, DNM1L gene), crucial for mitochondrial and peroxisomal division, and Methylcrotonoyl-CoA carboxylase beta chain (MCCB, MCCC2 gene), which is essential for leucine catabolism^30^, linked with obesity and insulin resistance^72^. Other proteins within this cluster include: Pyruvate dehydrogenase protein X component, mitochondrial (ODPX, PDHX gene), which plays a crucial role in maintaining the functionality of the pyruvate dehydrogenase complex, a key enzyme in cellular energy metabolism and Epoxide hydrolase 1 (HYEP, EPHX1 gene) involved in the metabolism of endogenous lipids such as epoxide-containing fatty acids. Additionally, it comprises also detected before using statistical approach Mitogen-activated protein kinase 1 (MK01, MAPK1 gene), a serine/threonine kinase pivotal in the MAP kinase signaling pathway^21^. Overall, this cluster is intricately connected to cellular metabolism, signaling, and metabolic stress responses.

Lastly, the Blue Cluster contains 26S proteasome regulatory subunit 6B (PRS6B, PSMC4 gene), crucial for the ubiquitin–proteasome system, which is vital for protein homeostasis. Dysregulation of this system is linked to various diseases, notably diabetes^73^. Other important proteins present within this cluster include Eukaryotic translation initiation factor 4B (IF4B, EIF4B gene), essential for mRNA translation initiation, and 60S ribosomal protein L30 (RL30, RPL30 gene), involved in protein synthesis. Among others, Glycerol-3-phosphate dehydrogenase 1 (GPDA, GPD1 gene) connects carbohydrate and lipid metabolism, contributing electrons to the mitochondrial electron transport chain^74^, Bifunctional glutamate/proline–tRNA ligase (SYEP, EPRS1 gene) is a multifunctional protein involved in the aminoacylation of tRNA molecules within the aminoacyl-tRNA synthetase multienzyme complex. Furthermore, Programmed cell death protein 5 (PDCD5) may play a role in apoptosis. The proteins in this cluster appear to be engaged in crucial cellular processes such as maintaining cellular structure, regulating signaling pathways, and facilitating protein synthesis.

Both methods underscore the importance of proteins involved in mitochondrial function and energy metabolism, a crucial aspect given the metabolic disruptions associated with prediabetes.

Despite these commonalities, the machine learning-based method tends to reveal more nuanced insights into cellular mechanisms, perhaps due to its ability to parse complex patterns in high-dimensional data. For example, it delineates proteins essential for cellular protein homeostasis like 26S proteasome regulatory subunit 6B (PRS6B, PSMC4 gene). Proteasomal dysfunction can exacerbate metabolic disorders in type 2 diabetes by creating an insulin-resistant signature in skeletal muscle tissue^75^. Studies in insulin-resistant models, have shown that enhanced proteasome activity can be a result of diminished signaling through IRS-1 and Akt, a phenomenon observed in insulin-resistant human muscle^76,77^. Additionally, abnormal ubiquitination in peripheral tissues like skeletal muscle can impair insulin-stimulated glucose metabolism, mitochondrial function, and muscle mass^78^. The machine learning approach highlights additional crucial proteins related to cellular structure and signaling, such as Eukaryotic translation initiation factor 4B (IF4B, EIF4B gene), which is essential for mRNA translation initiation. This indicates a possibly deeper exploration of the proteomic landscape compared to the traditional statistical approach used in statistical approach.

On the other hand, the pipeline tailored for proteomic data, while perhaps less detailed in distinguishing subtle biological themes, offers robust validation of key proteins identified by machine learning, thereby reinforcing the relevance of these proteins in prediabetes. It highlights a diverse array of proteins implicated in critical cellular functions as well as broader categories, spanning from structural integrity to signaling regulation, and stress and immune response mechanisms. These proteins collectively reflect the complex adaptive changes occurring in prediabetic conditions, suggesting a more generalized view in mechanisms of IR and T2D development.

## Material and methods

### Patients and groups

The subjects included in this experiment were selected from participants recruited as part of the “Bialystok Exercise Study in Diabetes”, conducted by the Department of Endocrinology, Diabetology and Internal Medicine and Clinical Research Centre of the Medical University of Bialystok, as described earlier^79,80^. The study cohort involved physically inactive men with various degrees of dysglycemia, living in the city of Bialystok, Poland. All participants were engaged in a 3-month exercise program that included supervised training sessions at a local fitness center, as described earlier^79,80^. The investigation followed the ethical principles outlined in the 1964 Declaration of Helsinki, along with its subsequent amendments and was approved by the Ethics Committee of the Medical University of Bialystok (No. R-I-002/469/2014). Each participant signed informed consent before sample collection.

The study cohort consisted of 32 men aged 35–65 years, with a Body Mass Index (BMI) of 25–35 kg/m^2^ and leading a sedentary lifestyle (assessed using the Polish version of International Physical Activity Questionnaire—Long Form (IPAQ-LF)^81^ with the ability to undertake physical activity.

The participants were divided according to American Diabetes Association and Polish Diabetes Association dysglycemia recommendations into three groups:

- NORMOGLYCEMIA (NG group; n = 13)—subjects with normal fasting glucose and normal glucose tolerance, defined as fasting plasma glucose (FPG) < 100 mg/dL and 2-h glucose during oral glucose tolerance test (2 h-OGTT-GLU) < 140 mg/dL.
- PREDIABETES (PD group; n = 11)—subjects with impaired fasting glucose and impaired glucose tolerance, defined as FPG 100–125 mg/dL and 2 h-OGTT-GLU 140–199 mg/dL.
- T2D (n = 8)—subjects with type 2 diabetes, diagnosed within last 3–5 years, treated with metformin only as an anti-diabetic drug.

For this study, we included only the post-exercise biopsies from all individuals. Analysis was conducted on 2 independent biopsies from each participant (total of 64 samples). Individuals within these groups were matched based on their age for, BMI, the number of training sessions completed during the intervention, and changes in daily kcal consumption (diet by confirming non-significant differences according to t-tests). For the group with type 2 diabetes, an additional inclusion criterion was Glycated hemoglobin (Hb1Ac) < 6.5% with only metformin approved in pharmacotherapy.

Exclusion criteria included smoking, drug or alcohol addiction, declared physical activity (highly active lifestyle), chronic diseases (with the exception of: hypertension, obesity with BMI ≤ 35 kg/m^2^, type 2 diabetes), pharmacotherapy (with the exception of the use of metformin by type 2 diabetics and angiotensin-converting-enzyme inhibitors due to hypertension), and abnormalities and medical contraindications to participate in planned exercise sessions found in the physical examination, including chronic neurovascular complications of diabetes. Additionally, there was no difference in the proportion of patients receiving angiotensin-converting-enzyme inhibitors as a treatment for hypertension between the groups.

The individuals of the Bialystok Exercise Study in Diabetes underwent clinical assessment, including an oral glucose tolerance test (OGTT), skeletal muscle and adipose tissue biopsy, and maximal cardiopulmonary exercise test (CPET), which were conducted both before and after the intervention. The tissue samples were then processed according to suitable protocols for proteomic assays as detailed below and in Appendix A.

### Sample preparation, LC/MS/MS analysis and data treatment

**Figure 8:**
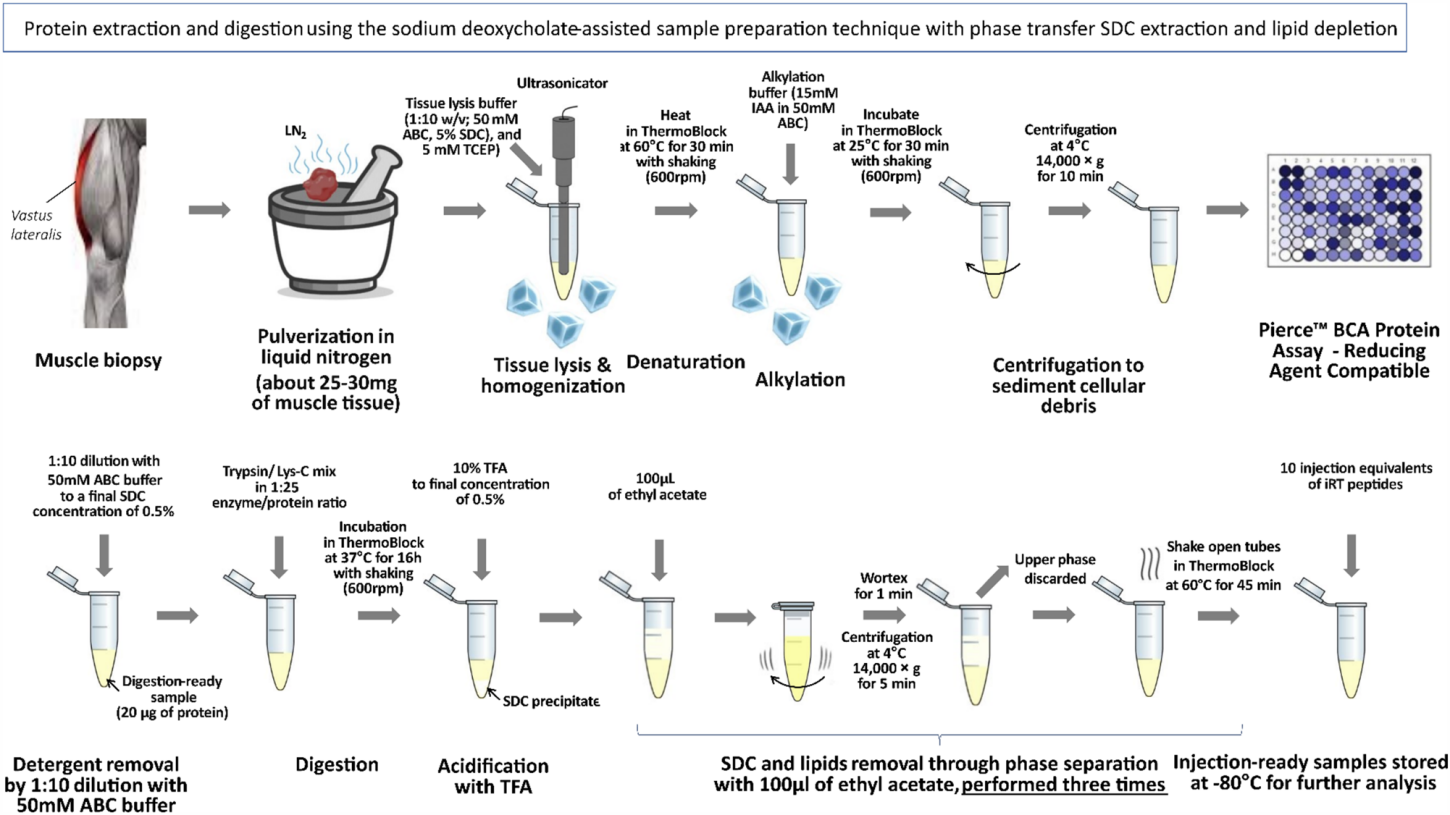
Samples preparation schema for the performed experiments.

The preparation of protein samples involved a sodium deoxycholate (SDC) assisted method with phase transfer extraction and lipid depletion, following the approach described by Leon et al.^82^ (see Fig. 8). About 25–30 mg of muscle tissue was pulverized in liquid nitrogen (LN_2_), suspended in a lysis buffer (1:10 w/v; containing 50 mM ammonium bicarbonate (ABC), 5% sodium deoxycholate (SDC), and 5 mM Tris(2-carboxyethyl)phosphine hydrochloride (TCEP)), sonicated, and then denatured by heating for 30 min at 60 °C. Reduced cysteine residues were alkylated with iodoacetamide (IAA) (1 M in ABC buffer, 15 mM final concentration) for 30 min at room temperature in the dark. After centrifugation, protein concentration was measured and normalized. Samples containing 20 µg of already reduced and alkylated protein mixture are tenfold diluted over initial conditions with 50 mM ABC buffer to 0.5% final concentration of SDC, then the proteases Trypsin/Lys-C mix (from 0.4 µg/µL stock) are added at 1:25 enzyme/protein ratio (1:50 individual enzyme/protein ratio). The digestion was performed in ThermoBlock at 37 °C for 16 h with shaking (600 rpm). Enzymes activity was inhibited by acidification with 10% trifluoroacetic acid (TFA) in LC/MS water up to a 0.5% concentration. SDC and lipids were removed by extraction, with 100 μL (1:1) of ethyl acetate (EA), repeated three times. Furthermore, 10 injection equivalents of iRT peptides were added to each sample. Injection-ready samples were stored at − 80 °C for further analysis.

Liquid-chromatography was performed on the Thermo Scientific 3500RSLC nanoLC system configured for trap-elute. Protein assignment was carried out through MS/MS analysis using a Q-Exactive MS instrument. Data-dependent (DDA) and independent analyses (DIA) were conducted. The staggered windows approach was applied for DIA method, with ion-chromatogram library constructed with gas-phase fractionation (GPF) approach.

Ion-chromatogram libraries from GPF runs were generated using Spectronaut version 15.4.210913.50606 (Rubin). The raw files were searched with the built-in Pulsar engine against the Homo sapiens Uniprot reference proteome FASTA file (UP000005640_9606, one sequence per protein) using Pulsar standard settings with a few modifications. Detailed description of proteomic analysis pipeline is presented in Appendix A.

The mass spectrometry proteomics data have been deposited to the ProteomeXchange Consortium via the PRIDE^83^ partner repository with the dataset identifier PXD051677.

### Functional enrichment analysis using string software

The network diagrams for Fig. 7 and additional figures in Appendix H, as well as the Functional Enrichment Analysis (supplementary materials), were generated using String software. The halo color is based on the occurrence of the protein in the submitted set in analyzing decision trees generated by the REHA algorithm or P-value (-Log10) in case of statistical analysis. We submitted data to normal gene set analysis using gene names with occurrence or P-value (-Log10) and following parameters:

- *FDR stringency*: medium (5%)
- *Initial sort order*: enrichment score
- *Organism*: homo sapiens
- *Network type*: full STRING network
- *Meaning of network edges*: confidence active interaction sources: Text mining, Experiments, Databases, Co‑expression, Neighborhood, Gene Fusion, Co‑occurrence
- *Minimum required interaction score*: medium confidence (0.500)
- *Max number of interactors to show*: 1 shell:—none/query proteins only -; 2shell:—none
- *Network display options*: hide disconnected nodes in the network,
- *Clustering Options*: k-means
- *Number of clusters*: 3, 6 or 6

### Machine learning methods

For machine learning methods, protein identification and quantitation using DIA approach was performed in the same manner as for proteomic-tailored analysis. As a input, we used individual protein expression values from each sample (corresponding to mean combined peptide signal), calculated after peptide identification, protein folding and FDR correction (1% FDR for both precursors and peptides at the experiment level and 5% for proteins at the run-wise level), without applying cut-offs for experiment-level differential expression protein Log2 fold change and -Log10 p-value. Seven classification models were used. The relative evolutionary hierarchical analysis (REHA)^10^ stands out as a progressive algorithm that merges different variants of Relative eXpression Analysis (RXA)^8^ systems and reshapes the way inter-feature relations are understood, with a specific focus on gene expression data. By introducing hierarchical gene clusters; subsets of gene families associated with related proteins; REHA enhances data classification and attribute selection in the genomic field.

At its core, REHA integrates the Top-Scoring Pair (TSP)^8^ methodology from RXA with a decision tree's multi-test approach, enriched by several advancements^9^. It embeds a predetermined discriminative power of genes, employs a nuanced two-level mutation within genetic operators, and employs a multi-objective fitness function that evaluates classification accuracy, cluster consistency, and protein rankings.

Adhering to a typical evolutionary algorithm structure^15^, REHA utilizes an unstructured population and generational selection, with superior genes being propagated through a linear ranking selection and an elitist approach^13^. The algorithm's knowledge is encoded in a hierarchical tree form, where internal nodes link to gene clusters and branches marks the diverging paths based on gene co-expression and epistatic interactions, leading to leaves denoted by class labels. The tree's splits, each defined as a gene cluster, identify a primary gene pair and several surrogate pairs that underpin the primary pair's division, ranked by how closely they resemble the primary pair's sub-group divisions. This idea is similar to the multi-test concept^11^. The data split within REHA follows a majority voting system where all gene pairs have equal influence, and surrogate pairs can potentially override the primary pair’s decision when there’s an equal split. These clusters incorporate a gene’s discrimination rank, which is influenced by tools like Relief-F and impacts the algorithm’s operation across various stages.

REHA's fitness function is designed to favor models that are accurate, with substantial gene clusters that have closely resembling gene pairs and maintain a low complexity in terms of the number of attributes in the cluster. This addresses the unique challenge in gene expression data of balancing the risk of underfitting with simple models and overfitting with complex ones. The evolutionary process of REHA includes selection, crossover, and mutation to develop decision trees, which are then pruned to achieve simplicity without sacrificing accuracy. The algorithm has been preliminarily validated on cancer-related gene expression datasets, where it outperformed existing RXA solutions. Its interpretability and direct applicability highlight REHA’s potential for revealing intricate genomic patterns and suggests its versatility for different omics data types.

In the course of our experiments, we primarily utilized the REHA algorithm; however, for comparative purposes, we also employed a range of other popular state-of-the-art solutions. This diverse selection included both black-box and white-box models, thereby covering a spectrum of machine learning approaches that range from highly interpretable to those that are more complex and less transparent in their decision-making processes.

The IntelliOmics platform^84^, was used to prepare and transform datasets used in the performed experiments. Next, the algorithms (except the REHA) were optimized and tested using WEKA software^85^. Each algorithm has its own set of specific parameters that can be tuned to improve the performance of the model. An automatic search for the best combination of parameter values was used by iterating over a range of possible values and testing each combination against a performance metric (such as accuracy or AUC) to see which produces the best results. The setup and fine-tuning of the parameters were carried out on a subset of the training dataset and performed using AutoWeka^86^. Here are short descriptions of each algorithm:

- *J48 Decision Tree*: The J48 decision tree is an implementation of the C4.5 algorithm^87^ in Java. It is known for its interpretability, as it creates a tree that can be visualized and understood, where each node represents a feature in the dataset, and each branch represents a decision rule. This ultimately leads to a decision output at the tree's leaves.
- *CART Decision Tree*: CART algorithm^88^ is similar to J48 but typically uses a binary tree structure. It is used for both classification and regression tasks and offers high interpretability. The model is considered a white-box because the path from the root to any leaf can be easily translated into an if–then rule.
- *JRip Rule Learner*: JRip is an implementation of the RIPPER algorithm^89^, a rule-based learning algorithm that is used for classification. It is considered a white-box model because the rules generated by JRip can be easily inspected and understood by humans.
- *Naive Bayes*: NB classifiers^90^ are a family of simple “probabilistic classifiers” based on applying Bayes' theorem with strong (naive) independence assumptions between the features. It is sometimes considered a white box model because the probability model and the effect of each feature on the final decision are straightforward to understand.
- *Random Forest*: RF^91^ is an ensemble learning method that constructs a set of decision trees at training time and outputs the mode of the classes for classification or mean prediction for regression of the individual trees. While individual trees are interpretable, the ensemble as a whole is often treated as a black-box due to the complexity of aggregating multiple decision paths.
- *Support Vector Machine*: SVM^92^ is an algorithm for solving the quadratic programming problem that arises during the training of Support Vector Machines. SVMs are typically considered black-box models, especially with non-linear kernels, as the decision function and support vectors do not provide a clear rule-based decision path.

Since the datasets were not pre-divided into training and testing parts, a typical tenfold Cross-Validation was performed, which is a standard technique used for validation. The process of classification was carried out without performing any feature selection beforehand, meaning that all available features or variables in the dataset were used in the model. Presented results show an average score of 50 runs due to the existence of nondeterministic algorithm—REHA. Along with the confusion matrix, the area under the curve (AUC) and ROC curve was generated for each solution.

### Supplementary Information

Supplementary Information 1.

Supplementary Information 2.

Supplementary Information 3.

## Data Availability

The mass spectrometry proteomics data have been deposited to the ProteomeXchange Consortium via the PRIDE 47 partner repository with the dataset identifier PXD051677. Data generated or analyzed during this study are included in the article and its supplementary Information files.

## Abbreviations

ABC: Ammonium bicarbonate
ACC: Accuracy
AUC: Area under the ROC curve
BMI: Body mass index
CART: Classification and regression tree
CPET: Maximal cardiopulmonary exercise test
DDA: Data-dependent analysis
DIA: Data-independent analysis
F1: F1-score
FDR: False discovery rate
LC/MS/MS: Liquid chromatography with tandem mass spectrometry
NB: Naive Bayes
NG: Normoglycemic
OGTT: Oral glucose tolerance test
PD: Prediabetes
REHA: Relative evolutionary hierarchical analysis
RF: Random forest
ROC: Receiver operating characteristic
RXA: Relative eXpression Analysis
SDC: Sodium deoxycholate
SVM: Support vector machine
T2D: Type 2 diabetes mellitus
WF1: Weighted F1-score
