# Supplementary material for: Exploring protein relative relations in skeletal muscle proteomic analysis for insights into insulin resistance and type 2 diabetes
Source: Sci Rep. 2024 Jul 31;14:17631. doi: 10.1038/s41598-024-68568-4 (PMC11292014; doi:10.1038/s41598-024-68568-4)
Supplement: Supplementary file 1 — Supplementary Information 1. [file 41598_2024_68568_MOESM1_ESM.docx]

Table of Contents

[Appendix A: Patients’ additional description 2](#_Toc170760806)

[Appendix B: Normoglycemic State vs. Prediabetes 7](#_Toc170760807)

[Appendix C: Proteins identified by the selected trees from REHA algorithm 16](#_Toc170760808)

[Appendix D: Proteins identified by the REHA algorithm in Figure 2 29](#_Toc170760809)

[Appendix E: Conventional statistical approach 33](#_Toc170760810)

[Appendix F: Protein details from statistics tailored for proteome data analysis in Figure 4 37](#_Toc170760811)

[Appendix G: Proteins identified by the REHA algorithm in Figure 6 54](#_Toc170760812)

[Appendix H: Network diagram from String software 58](#_Toc170760813)

[Appendix I: Network diagram of REHA approach 60](#_Toc170760814)

[Appendix J: ROC curves of REHA analysis 63](#_Toc170760815)

[Appendix K: References 63](#_Toc170760816)

# Appendix A: Patients’ additional description

*Exercise Intervention*

Studied patients underwent a 3-month exercise intervention comprising mixed training, aerobic, and strength exercises proven effective in diabetes prevention ^1–3^. Eligibility required completing at least 80% of planned sessions. Three weekly sessions, totaling 36 over 12 weeks, lasted 85 minutes. Participants attended three exercise sessions per week, finishing the program with a total of 36 completed workouts over a period of 12 weeks. Sessions included a 15-minute warm-up, 40 minutes of strength exercises covering major muscle groups, and 30 minutes of moderate-intensity endurance exercise tailored to subjects to achieve the target HR range corresponding to 60–70% of individual’s maximal oxygen consumption (VO2max). Resistance loads started at 60-75% of 1 Repetition Max, adjusted every three weeks based on progress. Three session types targeted different muscle groups weekly. Supervised by an exercise technician, the myWellness system (Technogym, Cesena, Italy) recorded each session for consistency. Dietary supervision aimed to eliminate diet-related variables impacting results.

*Clinical and biochemical analyses*

Participants underwent a clinical evaluation before and after a 3-month intervention in three visits. The first visit included an OGTT, the second involved a skeletal muscle biopsy and body composition measurements, and the third featured a CPET. All visits occurred in a fasting state within 48 hours of starting or ending the intervention.

*Exercise Testing*

Participants underwent a CPET on a treadmill (Quasar Med, h/p/cosmos, Germany) connected to the metabolic assay cart (Quark, Cosmed, Italy),using the Balke standard protocol to measure VO2max ^4^. The test determined physical fitness and set the target workload for intervention exercises.

*Biochemical Measurements*

- Anthropometrics and body composition measurements were taken in a fasted state using a using a calibrated SECA 264 stadiometer (SECA, Hamburg, Germany) and a SECA 769 electronic scale (SECA, Hamburg, Germany). BMI was determined by dividing body weight (in kilograms kg) by the square of height (in meters m). Body composition was assessed with whole-body dual-energy X-ray absorptiometry (DXA) scans using a Lunar iDXA device (GE Healthcare, Chicago, IL, USA). Measurements of lean body mass (LBM), fat mass (FM), and visceral adipose tissue mass (VAT mass) were obtained and presented in kilograms (kg).
- OGTT and Laboratory Measurements: The OGTT procedure was carried out following American Diabetes Association diagnostic criteria ^5^. Each patient began the test between 7:30-8:00 AM. The process began with the collection of an initial blood sample (0 min) and proceeded with the oral intake of a 75g glucose solution mixed in 300mL of room temperature water. Subsequent blood samples were taken at 30, 60, and 120 minutes after consuming the glucose solution for glucose and insulin level assessments. Plasma blood glucose levels were measured using the colorimetric method and a Cobas c111 device (Roche Diagnostics, Basel, Switzerland) as per the manufacturer's instructions. Insulin concentrations were determined using a commercial immunoradiometric assay (IRMA) kit, following the manufacturer's protocols (DIAsource ImmunoAssays SA, Louvain-la-Neuve, Belgium).

On the day of the biopsy, venous blood samples were taken prior to initiating the biopsy procedure. Laboratory measurements included serum triglycerides (TG), total cholesterol (TChol), high-density lipoprotein cholesterol (HDL), and low-density lipoprotein cholesterol (LDL) levels. These concentrations were measured using colorimetric methods with Cobas c111 kits, following the manufacturer's guidelines (Roche Diagnostics, Basel, Switzerland). Additionally, HbA1c levels were determined using a high-performance liquid chromatography (HPLC) method, in accordance with the manufacturer's instructions (Bio-Rad VARIANT, Bio-Rad Laboratories, Hercules, CA, USA).

- Glucose Homeostasis Derivates: the indices of insulin resistance (homeostatic model assessment for insulin resistance (HOMA-IR)) and beta cell function (homeostatic model assessment of beta cell function (HOMA-β)) were calculated from fasting insulin and glucose concentrations. HOMA-IR = fasting insulin concentration (µIU/mL) × fasting glucose concentration (mmol/L) / 22.5. HOMA-β = (20 × fasting insulin concentration (µIU/mL)) / (fasting glucose concentration (mmol/L) - 3.5) ^6^.

*Muscle Biopsy*

Biopsies were taken before and after the intervention, following standardized conditions, an overnight fast, and a 48-hour exercise abstinence. Samples were obtained from the vastus lateralis muscle using the modified Bergström technique ^7^. The procedure began by cleansing the puncture site's skin using 2% chlorhexidine in 70% isopropyl alcohol, followed by local anesthesia using 5 mL of 2% lignocaine injected into the skin and subcutaneous tissue. Vastus lateralis biopsies were obtained from the same leg but at different incision sites using a percutaneous needle and suction. Samples were visually inspected, and any excess blood, connective tissue, and fat were removed. Each sample's weight was then measured using a laboratory scale. Finally, all samples were snap-frozen and stored in liquid nitrogen until further analysis.

**Samples preparation**

Protein extraction and digestion were conducted using the sodium deoxycholate-assisted sample preparation technique with phase transfer SDC extraction and lipid depletion, following the approach described by Leon et al ^8^. Muscle samples were pulverized in liquid nitrogen (LN_2_), suspended in tissue lysis buffer (1:10 w/v; containing 50 mM ammonium bicarbonate (ABC), 5% sodium deoxycholate (SDC), and 5 mM Tris(2-carboxyethyl)phosphine hydrochloride (TCEP)) and sonicated on ice. Next, the samples were denatured and alkylated with iodoacetamide (IAA) (15mM final concentration). After centrifugation (1400g for 5 minutes), protein concentration was determined using the Bicinchoninic acid assay (reducing-agent compatible, Thermo Scientific) and samples were then stored at -80°C until further analysis.

Digestion-ready samples (20 μg of protein) were diluted 10x to a final SDC concentration of 0.5%. Subsequently, Trypsin/LysC mix was added at 1:25 ratio. Samples were digested at 37°C for 16h with shaking. After acidification with trifluoroacetic acid (TFA) to final concentration of 0.5%, SDC and lipids were removed through phase separation with 100μl of ethyl acetate, performed three times. Furthermore, 10 injection equivalents of iRT peptides were added to each sample for retention time calibration. Subsequently, 5 μg of peptide equivalent was taken to create a mixed sample used for gas-phase fractionation (GPF) for ion-chromatogram library generation. Injection-ready samples were stored at -80°C for further analysis.

**LC/MS/MS untargeted proteomics**

Liquid Chromatography with tandem mass spectrometry (LC/MS/MS) analysis using data-independent approach (DIA) was conducted using Thermo Scientific 3500RSLC nanoLC, Q-Exactive MS and IonFlex II source equipped with silica glass emitter (10μm I.D. PicoTip, New Objective, spray voltage 1.75 kV) operating in +ESI mode. Peptides (approx. 750ng) were loaded onto a µPAC™ trap (at 10 μl/min, 2% acetonitrile (ACN), 0.2% TFA) and eluted on a 50 cm µPAC™ column, at 300 nl/min (A – 0.2% formic acid) in H2O, LC/MS; B – 0.2% FA in 90% ACN, LC/MS; gradient 5%B for 4min, 30%B at 71min, 45%B at 94min, 78%B at 95min. DDA was performed using Top N method using following settings: MS1 - 70k, ACG - 3e6, IT 50ms, scan range 385-1015m/z; MS2 - 17k, ACG 2e5, IT 55ms, 2m/z isolation window ). Top 20 eluting peptides were fragmented with normalized collision energy (NCE) of 27 with ACG of 6e3 , intensity threshold 1.1e5 and dynamic exclusion set at 15sec. DIA method was based on the staggered windows approach by Searle, Pino, and Amodei ^9–12^. The method consisted of an MS1 scan covering the range of 385-1015 m/z, followed by 52 staggered, 24 m/z wide windows (with a 12 m/z overlap), covering the range of 400-1000 m/z with MS1 resolution at 35k, ACG at 1e6, and 60ms IT. MS2 resolution was set at 17k, AGC - 1e6, 60ms IT, MSX count of 1, isochronous IT set to on, and 27 NCE at default charge state of 3. Gas-phase fractionation for library generation comprised 6 overlapping fractions, each 102 m/z wide with a 2 m/z overlap. Each one contained 52 staggered, 4 m/z wide windows with a 2 m/z overlap.

Spectronaut version 15.4.210913.50606 (Rubin) was used for library generation and protein identification. Raw files were analyzed with Pulsar engine using Uniprot Homo sapiens reference proteome (UP000005640_9606, one sequence per protein) and the following settings: digestion with Trypsin/LysC, cut at P, carbamidometylation (fixed modification), deamidation of Q or N, acetylation of N-terminal, methylation of K or R, and oxidation of M (all as variable modifications), peptide length 7 to 35 AA, with maximum 5 variable modifications and 2 missed cleavages. Collected spectra were demultiplexed using bulid-in algorithm developed by Amodei et al. ^9^ and mass-calibrated using Pulsar engine dynamic calibration algorithm. The ion chromatogram library was constrained to include between 3 and 6 of the most intense fragments of b or y type per peptide, with an m/z range between 300 and 1800 and at least 3 amino acids in length. Universal false discovery rate (FDR) cutoff of 1% was set for precursors, peptide-spectrum matches, and proteins. Peptide retention time windows were calculated and calibrated using spiked-in iRT peptides and deep learning-assisted iRT regression.

Ion chromatograms from experimental samples were extracted from MS1 and MS2 data, based on maximum ion peak intensity, dynamic mass tolerances, and a dynamic, iRT-corrected retention time window. Subsequently, results were searched using previously constructed GPF ion-chromatogram libraries. A Q-value of 0.01, representing a 1% FDR, was applied for both precursors and peptides at the experiment level with a target-decoy approach involving mutated sequences. For proteins, the Q-value cutoffs was set at < 0.05 (5% FDR) at the run-wise level and < 0.01 (1% FDR) at the experiment-wise level. Both experimental samples and GPF library runs were analyzed using identical search engine settings. The quantification was conducted at the MS2 level using the built-in Spectronaut's MaxLFQ algorithm. Per-sample quantity of peptides (minor group) was calculated as a mean of the peak areas of the top 3 MS2 fragment ions, while the quantity of protein (major group) was determined as the mean of the top 3 peptides.

# Appendix B: Normoglycemic State vs. Prediabetes

In the analysis of proteomic data for distinguishing between NG and PD states, the first decision tree uses the relative expression levels of specific proteins as pivotal decision-making nodes. The tree structure is based on binary classifications, utilizing the interactions and functions of these proteins to infer metabolic states.

The initial comparison at the root of the tree is between 26S proteasome regulatory subunit 6B (PRS6B) and Mitogen-activated protein kinase 1 MAPK1/ERK2 (MK01). PRS6B is integral to the ubiquitin-proteasome system, essential for maintaining protein homeostasis and has been implicated in various diseases, including diabetes ^13^. Proteasomal dysfunction can exacerbate metabolic disorders in type 2 diabetes by creating an insulin-resistant signature in skeletal muscle ^14^. Wang et al. ^15^, in insulin-resistant mice model, demonstrated that heightened proteasome activity could be attributed to decreased signaling through IRS-1 and Akt, a phenomenon observed in insulin-resistant human muscle ^15,16^.

Conversely, MK01 is a serine/threonine kinase that serves as an essential component of the Mitogen-activated protein kinase cascade, influencing diverse cellular functions such as growth, differentiation, apoptosis and proliferation. Notably, overexpressed ERK can impair insulin signaling, leading to insulin resistance ^17^. The predominance of PRS6B over MK01 leads to a subsequent decision node, whereas a higher expression of MK01 shifts the analysis to an alternative pathway.

The next decision point involves comparing mitochondrial ATP synthase subunit d (ATP5H) with Caveolae-associated protein 4 (CAVN4). ATP5H is key in regulating skeletal muscle endocrine signaling and is associated with insulin resistance ^18^.

CAVN4 is important in caveolae formation, playing a role in insulin receptor-mediated signaling and glucose uptake in skeletal muscle cells ^19,20^. An elevated expression of ATP5H relative to CAVN4 is indicative of PD, whereas a lower expression points towards NG.

In the alternative branch where MK01 is more expressed than PRS6B, the focus is on the comparison of Cullin-5 ubiquitin ligase (CUL5) with Branched-chain-amino-acid aminotransferase (BCAT2). CUL5 is a scaffolding protein in active cullin-RING ubiquitin ligase (CRL) complexes, targeting multiple proteins involved in the ubiquitination process and subsequent proteasomal degradation ^21^. In recent times, Cullin-RING E3 ligases (CRLs) have been recognized as regulators of Insulin receptor substrate (IRS) protein turnover ^22^.

BCAT2, crucial in the catabolism of branched-chain amino acids: leucine, isoleucine, and valine. BCAAs and related metabolites has been identified as strong biomarkers of obesity, insulin resistance, type 2 diabetes (T2D) in humans ^23^. In this branch, a higher expression of CUL5 suggests a PD state, whereas a higher expression of BCAT2 leans towards NG.

In the second decision tree focusing on the differentiation between prediabetes (PD) and normal glucose tolerance (NG), a multi-test approach is utilized at the root. This approach involves a series of comparisons between pairs of proteins, with the outcome determined by unweighted voting. The key protein comparisons at the root are Apoptosis-inducing factor 1 (AIFM1) vs. 60S ribosomal protein L30 (RL30), Mitogen-activated protein kinase 1 (MK01) vs. Methylcrotonoyl-CoA carboxylase beta chain (MCCB), and Alpha-1-acid glycoprotein 1 (A1AG1) vs. D-dopachrome decarboxylase (DOPD).

AIFM1 is a flavin protein located in mitochondria, demonstrating demonstrates redox and pro-apoptotic effects, both of which are crucial for its significant involvement in metabolic disorders ^24^. AIFM1 is compared with a component of the large ribosomal subunit (RL30), which is involved in the synthesis of proteins in the cell.

MK01, involved in the MAPK signal transduction pathway, is contrasted with Methylcrotonoyl-CoA carboxylase beta chain (MCCB). 3-methylcrotonyl-CoA carboxylase (MCC) is a biotin-dependent mitochondrial enzyme essential for leucine catabolism ^25^ linked with obesity, insulin resistance, and dyslipidemia ^26^.

The third comparison between A1AG1, an acute phase protein associated with metabolic indicators ^27^ and DOPD, known for its roles in enhancing glucose uptake in the heart, underscores the connection between inflammatory processes and metabolic regulation ^28^. If the majority of these comparisons favor the first protein in each pair, the tree then evaluates the relationship between Methyltransferase-like protein 7A (MET7A) and ATP-dependent (S)-NAD(P)H-hydrate dehydratase (NNRD). A higher expression of MET7A, involved in methyl group transfer in various cellular processes ^29^, compared to NNRD, linked to mitochondrial function, suggests PD. Otherwise, NG is indicated ^30,31^.

On the other hand, if the initial majority vote does not favor the first proteins in the pairings, the decision tree compares Complement C4-A (CO4A) with Small nuclear ribonucleoprotein-associated proteins B and B' (RSMB). CO4A, a part of the classical complement pathway linked to the development of diabetes mellitus ^32^ is contrasted with RSMB, involved in RNA splicing processes and potentially related to various diseases ^33^. A higher expression of CO4A suggests PD, while a lower expression points towards NG.

In the third decision tree for differentiating prediabetes (PD) from normal glucose tolerance (NG) states, a multi-test approach is employed at the root. This approach involves comparing the relative expressions of various protein pairs: Short-chain specific acyl-CoA dehydrogenase (ACADS) vs. Creatine kinase B-type (KCRB), Growth hormone-inducible transmembrane protein (GHITM) vs. Direct IAP-Binding protein with Low PI protein (DIABLO), and Glycerol-3-phosphate dehydrogenase [NAD(+)], cytoplasmic (GPDA) vs. Aldo-keto reductase family 1 member B1 (ALDR).

ACADS, crucial in L-isoleucine metabolism and lipid (free fatty acids, triglycerides, and cholesterol) metabolism regulation ^34^, is compared with KCRB, which plays a significant role in ATP formation ^35^ and has been associated with insulin resistance ^36^. It is also correlated with glycated haemoglobin in a nondiabetic general population ^37^.

GHITM, a mitochondrial protein involved in oxidative capacity regulation ^38^ is contrasted with DIABLO, known for promoting apoptosis ^39^.

The comparison between GPDA, integral in connecting carbohydrate and lipid metabolism, serving as a contributor of electrons for the mitochondrial electron transport chain ^40^, and ALDR, involved in the polyol pathway and detoxification, underscores the interplay between carbohydrate and lipid metabolism in metabolic health ^41^.

If the majority of these tests favor the first protein in each pair, indicating a dominance of lipid metabolism, mitochondrial function, and carbohydrate metabolism, the tree assesses the relationship between Sar1b GTPase (SAR1B) and Proline-rich basic protein 1 (PROB1).

A higher expression of SAR1B GTPase, involved in protein transport to the Golgi apparatus ^42^, compared to PROB1, linked to condition characterized by the degradation of corneal collagen known as keratoconus ^43^, suggests PD. Otherwise, NG is indicated.

Alternatively, if the initial majority vote does not favor the first proteins in the comparisons, the decision tree contrasts Obg-like ATPase 1 (OLA1) with HIG1 domain family member 1A, mitochondrial (HIG1A). OLA1, involved in various cellular processes including response to oxidative stress ^44^, is compared with HIG1A, a regulator of mitochondrial function in hypoxic conditions ^45^. A higher expression of OLA1 indicates PD, suggesting an active response to cellular stress and growth regulation, while a lower expression points towards NG.

**Normoglycemic State vs. Type 2 Diabetes**

In the first decision tree addressing the NG vs. T2D states, the classification relies on a single test at the root involving the comparison between 40S ribosomal protein S6 (RS6) and Aldehyde dehydrogenase, mitochondrial (ALDH2).

RS6 is a component of the 40S ribosomal subunit, playing a crucial role in protein translation, and its interaction with the mTOR signaling pathway has implications for insulin signaling ^46^. ALDH2, on the other hand, is key in alcohol metabolism and mitochondrial oxidative ATP production, with research suggesting its role in overcoming insulin resistance ^47^. If RS6 is more expressed than ALDH2, it suggests a scenario favoring enhanced protein synthesis, potentially reflecting a state of normal glucose metabolism, hence classified as NG. This could be due to a more robust and efficient protein translation process, which is crucial for maintaining cellular function and metabolism in a non-diabetic state.

However, if ALDH2 expression surpasses that of RS6, the tree further evaluates the expression levels of Branched-chain-amino-acid aminotransferase (BCAT2) and Methyltransferase-like protein 7A (MET7A). BCAT2, involved in the catabolism of branched-chain amino acids, is linked to obesity, insulin resistance, and T2D. MET7A, responsible for transferring methyl groups to various molecules, has roles in cellular processes including cell survival and osteogenic differentiation ^29,48^*.* If BCAT2 is more expressed than MET7A, the classification is NG. Conversely, if MET7A exceeds BCAT2 in expression, the classification is T2D.

In the second decision tree addressing T2D vs. NG states, a multi-test approach is employed at the root. This approach involves comparing the relative expressions of pairs of proteins: Heterogeneous nuclear ribonucleoproteins C (HnRNP C) vs. Perilipin-4 (PLIN4), PLIN4 vs. Dual specificity mitogen-activated protein kinase kinase 3 (MP2K3), and Nucleoredoxin (NXN) vs. Dehydrogenase/reductase SDR family member 7 (DRS7B). HnRNP C, involved in RNA processing ^49^ and potentially influencing insulin sensitivity ^50^, is compared with PLIN4, that forms protective layer on the surface of lipid droplets and play a crucial role in regulating lipid turnover in muscle cells ^51^.

The repeated comparison of PLIN4 with MP2K3, a kinase activated by cytokines and environmental stress, essential for glucose transport, emphasizes the importance of lipid metabolism in insulin signaling and glucose homeostasis, that participates in the MAP kinase-mediated signaling pathway ^52,53^.

The third comparison is conducted between NXN, an oxidoreductase involved in redox balance ^54^ and adipogenesis diabetes ^55^, and DRS7B, part of the SDR family involved in diverse (patho)biochemical processes, encompassing intermediary metabolism and the transformation of foreign substances (xenobiotics) ^56^. If the majority of these tests favor the first protein in each pair, the classification is NG. This outcome implies a scenario where these metabolic processes are more pronounced in maintaining normal glucose levels. If not, the tree further compares Methyltransferase-like protein 7A (MET7A) with 26S proteasome regulatory subunit 7 (PRS7). If MET7A, involved in methyl group transfer and cellular processes ^29,48^, is more expressed than PRS7, a component of the ribosomal subunit involved in protein translation, the classification is T2D. This indicates a shift towards methylation activities and potentially altered protein synthesis, which are hallmarks of Type 2 Diabetes.

In the third decision tree for differentiating type 2 diabetes (T2D) from normal glucose tolerance (NG) states, a multi-test approach is employed at the root. This approach involves comparing the relative expressions of pairs of proteins: Perilipin-4 (PLIN4) vs. Endothelial differentiation-related factor 1 (EDF1), Proteasome subunit alpha type-2 (PSA2) vs. Eukaryotic translation initiation factor 1A, Y-chromosomal (IF1AY), and Ras-related protein R-Ras2 (RRAS2) vs. Very-long-chain 3-oxoacyl-CoA reductase (DHB12). PLIN4, a key regulator of lipid turnover, is compared with EDF1, which plays a role in endothelial cell differentiation and interacts with calmodulin ^57^. This comparison reflects the balance between lipid metabolism and endothelial cell function, crucial in the context of diabetes.

PSA2, part of the 20S core proteasome complex involved in protein degradation ^58^, is contrasted with IF1AY, essential for translation initiation ^59^, emphasizing the importance of protein homeostasis. The third comparison between RRAS2, involved in MAPK signaling and influencing insulin sensitivity ^60^, and DHB12, essential in long-chain fatty acid elongation ^61^, underscores the interplay between MAPK signaling pathway and lipid metabolism. If the majority of these tests favor the first protein in each pair, suggesting dominance of lipid metabolism, endothelial function, and signaling pathway control, the classification is NG. This indicates a scenario where these metabolic processes are more pronounced in maintaining normal glucose levels.

If not, the tree further compares Cytosolic 10-formyltetrahydrofolate dehydrogenase (AL1L1) with Ankyrin-3 (ANK3). AL1L1, involved in folate metabolism and crucial for metabolic balance ^62^, is compared with ANK3, an adaptor molecule linked to various cellular processes ^63^. A higher expression of AL1L1, indicating a focus on metabolic balance and folate metabolism, suggests T2D. Conversely, a higher expression of ANK3 leans towards NG, reflecting the role of ankyrins in cellular functions that maintain normal glucose metabolism ^64^.

**Prediabetes vs. Type 2 Diabetes**

In the first decision tree for PD vs. T2D comparison, the classification process starts with a comparison between Serum Amyloid P-component (SAMP) and Eukaryotic translation initiation factor 3 subunit F (EIF3F).

SAMP is a liver-secreted protein involved in immune response and inflammation, often detectable in amyloidosis cases ^65^. EIF3F is crucial for translation initiation, termination, and ribosomal recycling and its abnormal expression is linked to various diseases ^66^. If SAMP is more expressed than EIF3F, indicating a stronger presence of inflammation or immune response, the tree then compares Proteasome subunit alpha type-6 (PSA6) with Apolipoprotein E (ApoE).

PSA6 is involved in breaking down most intracellular proteins as a part of the proteasome complex, essential for cellular protein homeostasis ^58^. ApoE plays a central role in lipid balance and metabolism, impacting cholesterol, triglycerides, and phospholipids ^67^. If PSA6 is more expressed than ApoE, suggesting a higher focus on protein homeostasis over lipid metabolism, the classification is T2D. This could imply that alterations in protein degradation pathways are more prominent in T2D, compared to lipid metabolism imbalances typical of PD. Conversely, if EIF3F is more expressed than SAMP in the initial test, or if ApoE exceeds PSA6 in expression, indicating a stronger influence of lipid metabolism disturbances, the classification is PD. This reflects a scenario where lipid metabolism, particularly related to cholesterol and triglyceride management, is more crucial in the early stages of diabetes development, as seen in prediabetes.

In the second decision tree focusing on the T2D vs. PD problem, the initial classification step involves a multi-test comparing the expression levels of Very long-chain specific acyl-CoA dehydrogenase, mitochondrial (ACADV) with Transmembrane protein 11, mitochondrial (TMM11), Laminin subunit beta-1 (LAMB1) with Lon protease homolog, mitochondrial (LONM), and Ubiquitin carboxyl-terminal hydrolase isozyme L1 (UCHL1) with Interferon-inducible double-stranded RNA-dependent protein kinase activator A (PRKRA).

ACADV is a key enzyme in mitochondrial fatty acid beta-oxidation, whose dysfunction is linked to insulin resistance and T2DM ^68^. TMM11 is involved in mitochondrial quality control, particularly in mitophagy regulation, essential for cellular metabolism ^69^. LAMB1, an extracellular matrix (ECM) component, influences insulin activity ^70^, while LONM maintains mitochondrial protein quality and is implicated in metabolic disorders ^71^. UCHL1, primarily expressed in neurons and some cancers, is involved in protein processing and axonal integrity ^72^. PRKRA, linked to inflammation, integrates nutrient signals with the inflammatory response, influencing insulin resistance ^73^.

If the multi-test suggests more pronounced issues in mitochondrial metabolism, protein quality control, or inflammation (associated with ACADV, LAMB1, and UCHL1 being more expressed than their counterparts), the tree then compares Cocaine esterase (EST2) with Acetyl-coenzyme A synthetase 2-like, mitochondrial (ACS2L). EST2 is involved in xenobiotic detoxification and lipid metabolism detoxification ^74^, while ACS2L plays a crucial role in converting acetate into acetyl-CoA, pivotal in energy metabolism and lipid synthesis ^75^. If EST2 is more expressed than ACS2L, indicating a stronger focus on detoxification and lipid metabolism, the classification is T2D. Conversely, if the initial multi-test or the subsequent comparison suggests a stronger influence of mitochondrial dysfunction, protein quality control issues, or a focus on acetyl-CoA metabolism (associated with TMM11, LONM, PRKRA, and ACS2L being more expressed), the classification is PD. This implies a scenario where early-stage diabetes (prediabetes) is more characterized by challenges in mitochondrial function, protein homeostasis, and core energy substrate metabolism.

The final tree in the type 2 diabetes (T2D) vs. prediabetes (PD) problem involves a multi-test with several pairs of proteins: Glutathione S-transferase theta-1 (GSTT1) vs. Thioredoxin-like protein 1 (TXNL1), Very long-chain specific acyl-CoA dehydrogenase, mitochondrial (ACADV) vs. Transmembrane protein 11, mitochondrial (TMM11), and Ras-related protein Rab-5B (RAB5B) vs. Isocitrate dehydrogenase [NADP] cytoplasmic (IDHC). Depending on the expression levels of these proteins, the decision tree branches to either test Mth938 domain-containing protein (AAMDC) vs. Glycogen debranching enzyme (GDE) or Dolichyl-diphosphooligosaccharide--protein glycosyltransferase subunit 2 (RPN2) vs. T-complex protein 1 subunit delta (TCPD), to finally classify the condition as T2D or PD.

GSTT1 is involved in detoxification processes and cellular defense against oxidative stress ^76^, whereas TXNL1 plays a role in redox-regulating and anti-apoptotic mechanisms ^77,78^. Imbalances in oxidative stress and redox regulation are significant factors in insulin resistance and T2D. ACADV is central to fatty acid beta-oxidation in mitochondria, and its dysfunction is associated with insulin resistance and T2DM ^68^. TMM11 is involved in mitochondrial quality control, particularly in mitophagy, essential for metabolic balance ^69^*.*

RAB5B, part of the Ras GTPase family, is involved in MAPK signaling pathway regulation, which plays a role in insulin sensitivity and glucose uptake ^60^. IDHC is crucial in lipid metabolism and oxidative stress management ^79^.

AAMDC influences metabolic enzymes and PI3K-AKT-mTOR signaling, crucial for metabolic regulation and insulin sensitivity ^80^. GDE is essential in glycogen degradation, and its malfunction can contribute to the glucotoxicity seen in T2D ^81^.

RPN2 is involved in protein glycosylation and processing AGEs, which are significant in diabetes pathology. TCPD, a component of the TRiC complex, aids in protein folding, crucial for cellular function and metabolism ^82,83^.

The multi-test setup of the tree reflects the complexity of metabolic processes in distinguishing between T2D and PD. It underscores the importance of a balanced regulation of detoxification, oxidative stress, mitochondrial function, glycogen metabolism, and protein processing in the maintenance of metabolic health.

In the comprehensive analysis of the nine decision trees generated by the REHA algorithm for three distinct clinical problems – NG-PD (normal glucose tolerance to prediabetes), NG-T2D (normal glucose to type 2 diabetes), and T2D-PD (type 2 diabetes to prediabetes) – several intriguing patterns and distinctions emerge. These patterns highlight the complex interplay of metabolic pathways and cellular processes in the progression and regression of diabetes and prediabetic states.

For the NG-PD trees, a focus on proteins involved in cellular metabolism, signaling, and energy homeostasis like PRS6B, MK01, and ATP5H was evident. This suggests a shift in cellular dynamics as the body transitions from a normal glucose state to prediabetes, emphasizing proteasomal activity, MAPK signaling, and mitochondrial function. This shift in cellular energy dynamics and protein handling is critical as the body begins to lose its capacity to regulate glucose effectively.

In the NG-T2D trees, proteins such as RS6, ALDH2, and MET7A were central, highlighting the importance of ribosomal function, mitochondrial metabolism, and methylation processes. These proteins indicate the body's adaptation to prolonged hyperglycemia in T2D and the cellular adjustments necessary for reverting to normal glucose levels. The emphasis on mitochondrial and ribosomal proteins suggests a focus on rebalancing energy production and protein synthesis/turnover to reverse diabetic pathology.

The T2D-PD trees brought light proteins involved in mitochondrial function (like ACADV, TMM11), stress response (GSTT1, TXNL1), and insulin signaling (RAB5B, GDE). The presence of these proteins underscores the critical role of energy balance, oxidative stress management, and insulin pathway efficiency in shifting from type 2 diabetes to a prediabetic state. These proteins indicate a change in metabolic control and stress response, essential for moderating the severity of diabetes.

# Appendix C: Proteins identified by the selected trees from REHA algorithm

The table accompanying Figure 3 from the main text presents additional details on proteins identified by the REHA algorithm as significant for classification tasks among groups with NG-PD, NG-T2D, and T2D-PD. It includes information such as the protein descriptions; UniProtKB identifiers; gene names; and the functions of these proteins, summarizing their known biological roles and relevance to the conditions being studied. This comprehensive dataset is designed to shed light on the molecular biomarkers that differentiate these health states, enhancing the clarity and accessibility of the data for researchers interested in exploring the intricate biological mechanisms at play.

| **Tree (Figure 3)** | **Protein description** | **UniProtKB identifier** | **Gene name** | **Function** |
| --- | --- | --- | --- | --- |
| A1 | 26S proteasome regulatory subunit 6B  (PRS6B) | PRS6B_HUMAN | PSMC4 | The ubiquitin-proteasome system (UPS) serves as the primary non-lysosomal degradative machinery for most intracellular proteins ^84^.  As a crucial component of the UPS, the 26S proteasome plays a vital role in maintaining protein homeostasis by recognizing and eliminating damaged or misfolded proteins that could disrupt cellular activities and by removing unnecessary proteins ^13^. As a result, the proteasome is involved in various cellular processes, acting as a regulator of the cell cycle and cell division, immune responses and antigen presentation, apoptosis, and cell signaling.  Recent studies suggest that proteasome abnormalities continue to be implicated in several diseases. The 26S proteasome is either over-activated in certain cancers (such as multiple myeloma) or dysfunctional in neurodegenerative disorders (e.g., Alzheimer’s disease, Huntington’s disease), as well as amyotrophic lateral sclerosis ^85,86^**.** Research in various conventional diabetic animal models documented altered 26S proteasome activity in tissues based on in vitro protease-like activity assays. According to H. Liu et al. early hyperglycemia enhances 26S proteasome functionality *in vivo*, which elevates NF-κB-mediated endothelial inflammatory response in early diabetes with the resultant activation of NF-κB pathway leading to elevated inflammatory response as an early event in diabetes) ^84^.  Using 2-DIGE (two-dimensional difference gel electrophoresis) proteome analysis, Al-Khalili et al. ^14^ demonstrated that insulin-induced protein turnover is impaired in the skeletal muscle of type 2 diabetic patients due to abnormalities in the regulation of the proteins associated with the proteasome-degradation system. These findings emphasize the presence of abnormalities in cellular protein dynamics (protein homeostasis) that underlie metabolic disorders (associated with type 2 diabetes), which are exposed/uncovered through concurrent proteasome inhibition. Proteasomal dysfunction can exacerbate metabolic disorders in type 2 diabetes by creating an insulin-resistant signature in skeletal muscle ^14^. |
| A1, A2 | Mitogen-activated protein kinase 1  (MK01) | MK01_HUMAN | MAPK1 | MAPK signal transduction pathway proteins control various cellular processes such as growth, differentiation, apoptosis and proliferation. The biological response elicited by the MAPK pathway depends on factors such as cell type, the amplitude, and duration of the external stimulus.  In numerous cases of cancer and drug resistance, the MAPK/ERK pathway has been observed to be mutated or overexpressed. The dysregulation of ERK signaling is closely associated with tumorigenesis. Notably, overexpressed ERK can impair insulin signaling, leading to insulin resistance.  At high doses of insulin-like growth factor, active AKT phosphorylates RAF at the serine residue Ser259, thereby suppressing the activity of the RAF-MEK-ERK signaling pathway. Consequently, these pathways have become common possible for therapeutic intervention ^17^ |
| A1 | ATP synthase subunit d, mitochondrial (ATP5H) | ATP5H_HUMAN | ATP5PD | H+-ATP synthase activity is a pivotal regulator of skeletal muscle endocrine signaling. The expression and activity of H+-ATP synthase physiological inhibitor, ATPase inhibitory factor 1 (IF1), govern tissue homeostasis, metabolic reprogramming, and signaling. Within the muscle cell, IF1 acts as an intrinsic regulator of lipid metabolism during obesity and type 2 diabetes. Consequently, it can influence retrograde signaling to the nucleus, affecting programs related to myokine secretion and insulin resistance ^18^. |
| A1 | Caveolae-associated protein 4 (CAVN4) | CAVN4_HUMAN | CAVIN4 | Caveolae-associated protein 4 (CAVN4) is protein involved in the formation and function of caveolae, which are 50-100 nm sized invaginations in the cell membranes. Caveolae are crucial for multiple cellular functions such as endocytosis, cholesterol homeostasis, signal transduction, and mechano-protection ^19,20^. These structures have been shown to play a role in insulin receptor-mediated signaling and glucose uptake in skeletal muscle cells. Impaired function of caveolae can lead to insulin resistance. The expression of caveolins, particularly caveolin-1, along with insulin-related proteins were increased by resistance exercise training in the skeletal muscle of fifty-week-old Sprague Dawley rats. The upregulation of caveolin-1 was specifically associated with improved insulin sensitivity, especially in type 2 (EDL) muscle fibers. This suggests that the increase in the expression of caveolin-1 in skeletal muscle is dependent on the type of muscle fiber and the type of exercise performed, which may be important for improving insulin sensitivity in skeletal muscle ^19^. Caveolae-associated protein 4 is expressed predominantly in cardiac and skeletal muscle, but it is also present in low levels in other cell types, such as embryonic fibroblasts. It plays a role in regulating the morphology of caveolae in cardiomyocytes and participates in recruiting MAPK1/3 to caveolae within cardiomyocytes. CAVN4 activates MAPK1/3, which then regulates alpha-1 adrenergic receptor-induced hypertrophic responses in cardiomyocytes. It also contributes to the proper membrane localization and stabilization of caveolin-3 (CAV3) in cardiomyocytes. The distribution of Caveolae-associated protein 4 is perturbed/disrupted in human muscle disease associated with/ related to Caveolin-3 dysfunction. Muscle from a patient with mosaic expression of Cav3 showed a striking loss of sarcolemmal Cavin-4/MURC in the Cav3-negative fibers, which suggests that mutations in Caveolae-associated protein 4 may directly cause muscle disease, identifying Cavin-4 as a new candidate protein for caveolin-related muscle disease. Investigating/Studying the cavin complex in muscle diseases of unknown etiology may provide insights into caveolinopathies, in which/where mutations in Cav3 are not the primary defect ^87^. |
| A1 | Cullin-5 (CUL5) | CUL5_HUMAN | CUL5 | Cullin-5 ubiquitin ligase (Cul5) has a range of substrates that are essential for the ubiquitination process and subsequent proteasomal degradation. It plays a role in controlling cell growth, proliferation, and various physiological functions within the human body ^21^. In recent times, Cullin-RING E3 ligases (CRLs) have been recognized as regulators of IRS protein turnover. CRLs become active upon cullin neddylation, involving the covalent binding of a ubiquitin-like protein known as Nedd8 to a cullin scaffold. C. Chen et al. reported that pharmacological inhibition of cullin neddylation by NAE1 inhibitor (MLN4924, Pevonedistat) enhances hepatic insulin signaling and attenuates hyperglycemia in mice (rapidly decreases hepatic glucose production and lowers blood glucose in mice). Inhibiting neddylation slows down cullin-RING E3 ligase–mediated insulin receptor substrate protein degradation. This directly addresses a critical underlying issue contributing to hepatic insulin resistance. Pharmacological inhibition of cullin neddylation offers a treatment strategy for enhancing hepatic insulin signaling and managing high blood sugar levels ^22^. |
| A1, B1 | Branched-chain-amino-acid aminotransferase, mitochondrial (BCAT2) | BCAT2_HUMAN | BCAT2 | Branched-chain-amino-acid aminotransferase (BCAT2) initiates the first step in catabolism of the crucial branched-chain amino acids: leucine, isoleucine, and valine. Additionally, it can act as a transporter/carrier of branched-chain alpha-keto acids, as suggested by similarities with other related enzymes. BCAAs and related metabolites are now widely recognized as being strong biomarkers of obesity, insulin resistance, type 2 diabetes (T2D), and cardiovascular diseases in humans. Studies on rodents have demonstrated that lowering the levels of BCAA and branched-chain ketoacid (BCKA) through dietary restrictions or activating the rate-limiting enzyme in BCAA catabolism, BCKDH, can have positive effects on the regulation of glucose and lipid metabolism. However, the effects of BCAA restriction in human T2D subjects have been found to be less pronounced in short-term studies ^23^. |
| A2 | Apoptosis-inducing factor 1, mitochondrial (AIFM1) | AIFM1_HUMAN | AIFM1 | Apoptosis-inducing factor 1 (AIFM1), a flavin protein located in mitochondria. AIFM is originally known to induce apoptosis in response to pro-apoptotic factors.  AIF, functioning as a mitochondrial flavin adenine dinucleotide-dependent oxidoreductase, plays a role in controlling various aspects of mammalian cell metabolism. This includes the regulation of respiratory enzyme activity, management of oxidative stress, facilitation of mitochondrial autophagy, and enhancement of glucose uptake, among other functions. The AIF demonstrates redox and pro-apoptotic effects, both of which are crucial for its significant involvement in metabolic disorders.  AIF, as an apoptotic-inducing factor, can trigger/initiate programmed cell death in response to apoptosis signals, thereby serving as the underlying pathological cause of certain metabolic disorders like diabetes. AIF also affects the assembly and stability of complex 1 and mitochondrial function. AIF mutations directly lead to mitochondrial dysfunction and metabolic diseases, while abnormal expression can alter tumor cell metabolism by affecting the respiratory chain. However, AIF-mediated apoptosis or metabolic functions affect each other. Disrupting metabolic function can unintentionally induce apoptosis, and interfering with apoptotic function has the potential to pathologically alter metabolism. Therefore, the mechanisms underlying AIF-influenced metabolic diseases are multifaceted and intricate ^24^. |
| A2 | 60S ribosomal protein L30  (Large ribosomal subunit protein eL30) (RL30) | RL30_HUMAN | RPL30 | 60S ribosomal protein L30 (RL30) is a component of the large ribosomal subunit engaged in the synthesis of proteins in the cell. |
| A2 | Methylcrotonoyl-CoA carboxylase beta chain, mitochondrial (MCCB) | MCCB_HUMAN | MCCC2 | 3-methylcrotonyl-CoA carboxylase (MCC) is a biotin-dependent mitochondrial enzyme essential for leucine catabolism in the majority of living organisms ^25^. MCC deficiency can result in disruptions to leucine metabolism, leading to a range of potential manifestations, including neurological disorders, infant mortality, and asymptomatic adults ^88–91^. A study conducted on mice revealed an association between methylcrotonoyl-CoA carboxylase 2 (MCCC2), a subunit of 3-methylcrotonyl-CoA carboxylase (MCC), and the presence of obesity, insulin resistance, and dyslipidemia ^26^. Furthermore, the deletion of MCC suggests alterations in glycolysis, the tricarboxylic acid (TCA) cycle, oxidative phosphorylation (OXPHOS), gluconeogenesis, β-oxidation, and the metabolism of branched-chain fatty acids ^92,93^. |
| A2 | Alpha-1-acid glycoprotein 1 (A1AG1) | A1AG1_HUMAN | ORM1 | Alpha(1)-Acid glycoprotein (AGP) is considered an acute phase protein in all mammals studied so far. During an acute phase response, which is the systemic answer to a local inflammatory stimulus, the serum levels of AGP increase several-fold.  Moreover, its glycosylation pattern can vary depending on the type of inflammation. The precise biological role of this protein remains unclear, although it has been observed to influence various functions in different types of blood cells. *In vivo* studies have demonstrated the protective effects of AGP in multiple inflammation models ^94^.  Freitas de Medeiros et al. found that in individuals with PCOS, there is an association between Alpha-1-acid glycoprotein 1 (A1AG1) and indicators of adiposity, carbohydrate metabolism, and total testosterone levels. This inflammatory marker also demonstrates a correlation with erythrocyte sedimentation rate, neutrophil counts, and lymphocyte counts, which are frequent markers of an inflammatory condition ^27^. |
| A2 | D-dopachrome decarboxylase (DOPD) | DOPD_HUMAN | DDT | D-Dopachrome tautomerase (D-DT, MIF-2), also known as D-Dopachrome decarboxylase (DOPD), was serendipitously identified during an examination of tautomerization reactions of L-dopachrome in cultured melanoma cells, using D-dopachrome as a supposed control substrate ^95^. D-dopachrome tautomerase (DDT) is an enzyme found in mammalian cells, and although it doesn't have natural substrates in physiological processes, it does exhibit some degree of sequence and structural similarity with macrophage migration inhibitory factor (MIF) ^28^. Macrophage migration inhibitory factor (MIF) and its recently identified structural homolog, D-dopachrome tautomerase (D-DT), also referred to as MIF-2, are atypical cytokines and chemokines that play crucial roles in immune responses within the host. Moreover, they play significant roles in the development of acute and chronic inflammatory conditions, cardiovascular complaints, pulmonary diseases, inflammation in adipose tissue, and cancer ^96^.  Qi et al. ^28^ have presented preliminary evidence suggesting that the use of recombinant D-dopachrome tautomerase (rDDT) treatment exhibits a cardioprotective effect during ischemia-reperfusion in normal mouse hearts. Notably, even though both DDT and insulin enhance glucose uptake in the heart, they employ different mechanisms for doing so. DDT's action is mediated through AMPK, while insulin's action is mediated via PI3K. These signaling pathways differ significantly in various aspects: AMPK preserves ATP by promoting substrate breakdown, inhibiting protein synthesis, and promoting autophagy. In contrast, PI3K promotes the storage of glucose and fatty acids, stimulates protein synthesis, and inhibits autophagy through mTOR activation. Further investigations are necessary to determine whether rDDT is effective in conditions like diabetes ^28^. |
| A2, B1, B2 | Methyltransferase-like protein 7A (MET7A) | MET7A_HUMAN | METTL7A | The methyltransferase-like (METTL) proteins in humans are responsible for the transfer of methyl groups to nucleic acids, proteins, lipids, and other small molecules. This activity is integral to a range of cellular processes ^29^. E. Lee et al. discovered a new factor, METTL7A, through an investigation into glucose metabolism regulation. Their research unveiled previously undisclosed functions of METTL7A, which include promoting cell survival and stimulating the osteogenic differentiation of human bone marrow-derived mesenchymal stem cells (hBMSCs). It is anticipated that overexpressing METTL7A may serve as a protective mechanism for implanted cells when they encounter metabolic stress resulting from glucose deficiency ^48^. |
| A2 | ATP-dependent (S)-NAD(P)H-hydrate dehydratase (NNRD) | NNRD_HUMAN | NAXD | ATP-dependent (S)-NAD(P)H-hydrate dehydratase (NNRD) catalyzes the dehydration of the S-form of NAD(P)HX using ATP, which is subsequently converted into ADP. When working in conjunction with NAD(P)HX epimerase, which facilitates the conversion of both S- and R-forms, the enzyme enables the repair of both epimers of NAD(P)HX. The ATP-dependent dehydratase is known to be the product of the Carkd (carbohydrate kinase domain) gene in vertebrates ^30^. Mutations in this enzyme can result in impaired repair of NADHX and lead to mitochondrial dysfunction in humans, which is linked to a fatal neurometabolic disorder that manifests in early childhood ^31^. |
| A2 | Complement C4-A (CO4A) | CO4A_HUMAN | C4A | Complement C4-A (CO4A) is a non-enzymatic component of C3 and C5 convertases, playing a crucial role in sustaining the classical complement pathway. It forms covalent bonds with immunoglobulins and immune complexes, which, in turn, promotes the dissolution of immune aggregates and aids in the removal of immune complexes through CR1 receptors on erythrocytes. The C4A isotype is particularly effective in binding and forming amide bonds with immune aggregates or protein antigens.  A growing body of experimental and clinical data supports a link between complement system activation and the development of diabetes mellitus. Various elements within the complement system, including C3, C4, factor D, and CD59, have been demonstrated to play a role in the development of diabetes mellitus. D. E. McMillan has found that levels of C4 and C3 are both increased in patients with diabetes ^97^. Complement C4 has been relatively less studied in the context of metabolic disorders. However, existing data indicate its involvement in Type 1 diabetes (T1DM). Regulating the activation of the complement system may potentially decelerate the progression of diabetes mellitus ^32^. |
| A2 | Small nuclear ribonucleoprotein-associated proteins B and B' (RSMB)  Small nuclear ribonucleoprotein-associated protein N (RSMN) | RSMB_HUMAN  RSMN_HUMAN | SNRPB  SNRPN | Small nuclear ribonucleoproteins are protein–ribonucleic acid (RNA) complexes composed of a core noncoding RNA containing approximately 100 to 600 nucleotides and tightly bound proteins that together accumulate within the cell nucleus. SnRNPs are primarily recognized for their involvement in RNA splicing processes, which includes small ribonucleoprotein particles (snRNPs) such as U1, U2, U4/U6, and U5 that are present in the spliceosome. Because of their significant biological functions, numerous diseases are linked to snRNPs, making them potential indicators for both diagnosis and prognosis ^33^. |
| A3 | Short-chain specific acyl-CoA dehydrogenase, mitochondrial (ACADS) | ACADS_HUMAN | ACADS | Short/branched chain specific acyl-CoA dehydrogenase (ACADSB) is essential in the metabolism of L-isoleucine as it facilitates the dehydrogenation of 2-methylbutyryl-CoA, which is one of the reactions involved in breaking down L-isoleucine via the catabolic pathway. ACADSB has been shown to have a regulatory effect on the metabolism of free fatty acids (FFAs), triglycerides (TGs), and cholesterol (CHOL). ACADSB is a pivotal gene, which controls the expression of other genes such as ACADL and ACOX2, playing a key role in lipid metabolism ^34^. |
| A3 | Creatine kinase B-type (KCRB) | KCRB_HUMAN | CKB | Creatine kinase (CK) plays an important role in the formation of adenosine triphosphate (ATP), which is necessary for energy-demanding processes in human and animal cells, especially muscle contractions ^35^.  Intense and prolonged muscular activity, as well as eccentric muscular training, can significantly increase the levels of CK ^98,99^, whereas CK levels may only increase modestly by around 5% with leisure physical exercise ^36^.  Studies in both animals and humans have shown that Creatine kinase (CK) is associated with insulin resistance and has been identified as a risk marker for cardiovascular disease, primarily due to its relationship with hypertension and increased body mass index ^36^. It was also correlated with glycated haemoglobin in a nondiabetic general population ^37^. |
| A3 | Growth hormone-inducible transmembrane protein (GHITM) | GHITM_HUMAN | GHITM | Growth hormone-inducible transmembrane protein (GHITM) is a mitochondrial protein belonging to the TMBIM family and the BAX inhibitor-1 (BI1) superfamily. Its expression is widespread, with notably high levels in the brain, heart, liver, kidney, and skeletal muscle, while being less prevalent in the intestines and thymus ^34^.  Orliaguet et al. offered valuable insights into the mechanism by which the inflammatory transcription factor IRF5 influences the physiological adaptive response to diet-induced obesity through the regulation of mitochondrial structure in macrophages. They demonstrated that Interferon Regulatory Factor 5 (IRF5) plays a pivotal role in regulating the oxidative capacity of macrophages when faced with excess calories (in response to caloric excess). Macrophages lacking Irf5 gene in mice exhibit heightened oxidative respiration and increased mitochondrial membrane potential. They have shown that the elevated oxidative activity in Irf5-deficient macrophages results from transcriptional repression of the mitochondrial matrix component Growth Hormone Inducible Transmembrane Protein (GHITM) gene. The increased oxygen consumption associated with Irf5 deficiency could be mitigated by experimentally reducing Ghitm expression. Furthermore, in individuals with obesity or type-2 diabetes, both adipose tissue macrophages (ATMs) and monocytes maintain the reciprocal relationship between Irf5 and Ghitm ^38^. |
| A3 | Diablo IAP-binding mitochondrial protein (DBLOH) | DBLOH_HUMAN | DIABLO | DIABLO (Direct IAP-Binding protein with Low PI) proteins, also referred as Smac (Second Mitochondria-derived Activator of Caspases) and their homologues) is a mitochondrial protein that promotes apoptosis by activating caspases in the cytochrome c/Apaf-1/caspase-9 pathway, and by opposing the inhibitory activity of inhibitor of apoptosis proteins (XIAP-BIR3). Inhibits the activity of BIRC6/bruce by inhibiting its binding to caspases ^39^. |
| A3 | Glycerol-3-phosphate dehydrogenase [NAD(+)], cytoplasmic (GPDA) | GPDA_HUMAN | GPD1 | Glycerol-3-phosphate dehydrogenase (GPDH) is an enzyme responsible for facilitating the reversible oxidative transformation of dihydroxyacetone phosphate into sn-glycerol 3-phosphate ^100^. Glycerol-3-phosphate dehydrogenase plays a crucial role in connecting carbohydrate and lipid metabolism, serving as a significant contributor of electrons for the mitochondrial electron transport chain. Glycerol-3-phosphate dehydrogenase is essential for lipid biosynthesis. Through the reduction of dihydroxyacetone phosphate into glycerol 3-phosphate, GPDH is indispensable for the prompt dephosphorylation of glycerol 3-phosphate into glycerol. Moreover, GPDH is among the enzymes responsible for sustaining the redox potential within the inner mitochondrial membrane ^40^. mGPDH is abundantly expressed in various tissues and organs, with previous research showcasing its regulatory influence on pancreatic β-cells ^101^ and thyroid cancer cells ^102^, in addition to its participation in inflammatory reactions ^103^. It possesses a crucial role for mGPDH in hepatic lipid homeostasis ^104^ and muscle repair ^105^. It possesses a cardinal role for mGPDH in hepatic lipid homeostasis and muscle repair. In a study investigating skeletal muscle regeneration in the context of diabetic conditions, significant alterations were noted in the urea albumin-to-creatinine ratio (UACR) in response to changes in the mGPDH gene, indicating / suggesting potential connections between mGPDH and diabetic kidney disease (DKD) ^106^. |
| A3 | Aldo-keto reductase family 1 member B1 (ALDR) | ALDR_HUMAN | AKR1B1 | The aldose reductase (AR; human AKR1B1/mouse Akr1b3), has garnered significant attention in research due to its involvement in diabetic complications. These changes result from the substantial influx of glucose into the polyol pathway, where it is transformed into sorbitol by this enzyme. However, the role of AR in non-diabetic states remains a matter of uncertainty.  Members of the AKR1B/Akr1b family collectively possess a common capacity to enzymatically reduce a wide range of substrates, including aldehydes provided from lipid peroxidation, steroids, their derivatives, and xenobiotics in in vitro. Due to these properties, AKR1B/Akr1b are typically categorized as detoxification enzymes. Based on these, AKR1B/Akr1b enzymes are generally considered as detoxifying agents. Gain/loss-of-function models for the Akr1b7 isoform have demonstrated its involvement in metabolic processes and the regulation of adipose tissue homeostasis. In addition, recent findings suggest the functional capability of certain isoforms to either directly (prostaglandins) or indirectly (fatty acids) generate signal molecules ^41^. |
| A3 | Sar1b GTPase (GTP-binding protein SAR1b) (SAR1B) | SAR1B_HUMAN | SAR1B | Sar1b GTPase (GTP-binding protein SAR1b (SAR1B)) is an essential element within COPII vesicles that bud from the endoplasmic reticulum, enabling the transportation of proteins to the Golgi apparatus. Its genetic mutations are responsible for causing a serious condition called chylomicron retention disease ^42^. Marcil et al. demonstrated that in a mouse model, Sar1b was not influenced by insulin resistance or a high-fat diet. They also emphasized the significance of Sar1b in organs involved in lipid transport and/or calcium trafficking, including the liver, intestine, skeletal muscle, and heart ^42^.  Furthermore, Auclair et al. ^107^ have shown that homozygous alteration of Sar1b in mice impacts their postnatal survival and reveals abnormalities in numerous biochemical, metabolic, and developmental processes in relation to diet, sex, and Sar1b genotype ^107^. |
| A3 | Proline-rich basic protein 1 (PROB1) | PROB1_HUMAN | PROB1 | Proline-rich basic protein 1 is present in 89 different types of tissues throughout the human body, exhibiting the highest expression in the skeletal muscle of the leg and the cardiac muscle of the heart. It is associated with keratoconus, a condition characterized by collagen-related degeneration of the cornea. In a study using segregation analysis methodology, variants of PROB1 within the 5q31.1-q35.3 linkage region were found to completely segregate with the keratoconus phenotype. Furthermore, PROB1 expression is notably elevated in various disease conditions, including head and neck cancer and prostate inflammation ^43^. |
| A3 | Obg-like ATPase 1 (OLA1) | OLA1_HUMAN | OLA1 | Obg-like ATPase 1 (OLA1) belongs to the Obg family and falls within the YchF subfamily of P-loop GTPases, which are associated with regulating a wide range of cellular processes, such as protein translation, intracellular transportation, signal transduction, and cell growth. Notably, in contrast to other members of the Obg family, OLA1 exhibits a greater affinity for binding and hydrolyzing ATP than GTP.  OLA1 has a multifaceted role in various cellular responses. In HeLa cells, OLA1 acts as a negative regulator in response to oxidative stress. OLA1 binds to and protects heat shock protein 70 (HSP70) from being ubiquitin-dependently degraded by C-terminus of Hsp70-binding protein (CHIP) during heat shock stress. In mice, OLA1 stabilizes mitochondria SOD2 in pulmonary artery smooth muscle cells (SMCs) by binding to HSP70. Loss of OLA1 leads to SOD2 deficiency and an increase in the protein level of X-linked inhibitor of apoptosis (XIAP), promoting proliferation of pulmonary artery SMCs. OLA1 is required for the proliferation of mouse embryonic fibroblasts by attenuating the translation of p21, a cyclin-dependent kinase (CDK) inhibitor. In hepatocellular carcinoma cells, OLA1 promotes proliferation by binding with p21 and upregulating CDK2 expression. OLA1 plays a role in promoting cell migration by regulating the expression of E-cadherin, which is essential for intercellular adhesion, and focal adhesion kinase (FAK) in nonvascular cells. Overexpression of OLA1 is associated with tumor progression and poor survival in patients with hepatocellular carcinoma, colorectal cancer, endometrial cancer, and lung cancer. It also enhances drug resistance by inducing epithelial-to-mesenchymal transition through activation of the TGF-β/Smad signaling pathway in breast cancer. OLA1 expression is upregulated in cultured human ventricular cardiomyocytes and mouse heart after angiotensin II-induced hypertrophic response. Loss of OLA1 leads to the activation of the GSK3β/β-catenin signaling pathway, attenuating angiotensin II-induced hypertrophic response in human ventricular cardiomyocytes ^44^. |
| A3 | HIG1 domain family member 1A, mitochondrial (HIG1A) | HIG1A_HUMAN | HIGD1A | HIG1 domain family member 1A, mitochondrial (HIG1A) is a crucial regulator of conditions associated with hypoxia. Mitochondria hold a central position in the conversion of cellular energy, metabolic processes, and cell proliferation. The regulation of mitochondrial function by HIGD1A, a protein located on the inner mitochondrial membrane, is crucial for ensuring cell survival in low oxygen conditions (hypoxia). Serving as a novel regulator, HIGD1A preserves mitochondrial integrity, augments cell viability during hypoxia, and enhances resistance to oxygen deprivation. HIGD1A primarily targets cytochrome c oxidase, controlling downstream signaling pathways that impact the ATP production system, subsequently modifying mitochondrial respiratory function. Furthermore, HIGD1A assumes a dual role in cell survival within different degrees of hypoxia in tumor environments. In mild and moderate hypoxic regions, HIGD1A functions as a positive regulator, supporting cell growth. Conversely, in severe hypoxic areas, HIGD1A inhibits cell growth but maintains cellular activity. It is postulated that HIGD1A is involved in mechanisms related to tumor recurrence and drug resistance ^45^. |
| B1 | 40S ribosomal protein S6 (Small ribosomal subunit protein eS6) (RS6) | RS6_HUMAN | RPS6 | 40S ribosomal protein S6 (RPS6) is a component of the 40S ribosomal subunit and is therefore involved in translation.  Although the mechanism is not yet fully understood, mTOR may interact with eIF3, to regulate the association of eIF3 and eIF4G. Insulin increases eIF4E binding to eIF4G by enhancing 4EBP1 phosphorylation via mTOR signaling, resulting in more eIF4F bound to the mRNA's 5′ cap region. By promoting eIF4G binding to eIF3, it is expected that the joining of eIF4F and the 40S ribosome will be enhanced, thereby increasing the rate at which the small ribosomal subunit scans and positions itself on the mRNA to initiate translation ^46^. |
| B1 | Aldehyde dehydrogenase, mitochondrial (ALDH2) | ALDH2_HUMAN | ALDH2 | Aldehyde dehydrogenase, mitochondrial (ALDH2) is a key enzyme that is involved in alcohol metabolism and mitochondrial oxidative ATP production ^108^. In the context of alcohol-induced cardiomyopathy, an increase in ALDH2 expression has the potential to overcome insulin resistance within the hearts of mice subjected to chronic alcohol ingestion. This effect was achieved by enhancing insulin signaling ^47^. |
| B2 | Heterogeneous nuclear ribonucleoproteins C-like 1, 2 ,3, 4, C1/C2 (HNRC1), (HNRC2), (HNRC3), (HNRC4), (HNRPC) | HNRC1_HUMAN  HNRC2_HUMAN  HNRC3_HUMAN  HNRC4_HUMAN  HNRPC_HUMAN | HNRNPCL1  HNRNPCL2  HNRNPCL3  HNRNPCL1  HNRNPC | Heterogeneous nuclear ribonucleoproteins C (HnRNP C) belongs to the hnRNP family of RNA-binding proteins. Based on available public information, HnRNP C has the potential to facilitate the processing of nascent heterogeneous nuclear RNAs (hnRNAs, also known as pre-mRNAs) into mature mRNAs and may also play a role in enhancing the stability of mRNAs, thereby regulating their translation ^49^.  Zhao M. offered the initial evidence of a significant role played by hnRNP A1 in counteracting insulin resistance and hepatic steatosis triggered by a high-fat diet (HFD). HnRNP A1 is indispensable for preserving insulin sensitivity in muscle cells, as it has the ability to interact with gys1 mRNA, thereby enhancing the stability of this mRNA. Consequently, the selective targeting of hnRNP A1 or the hnRNP A1-gys1 interaction in skeletal muscle holds promise as a therapeutic approach for addressing insulin resistance and associated metabolic irregularities ^50^.  Furthermore, Gui et al. identified hnRNPA1 as an interacting partner of LncRNA H19, and they showed that LncRNA H19 functions in lipid metabolism through interaction with hnRNPA1 to increase the translation of fatty acid oxidation-related genes, including CPT1b and PGC1a ^109^. |
| B2, B3 | Perilipin-4  (PLIN4) | PLIN4_HUMAN | PLIN4 | Perilipins (PLINs) form a protective layer on the surface of lipid droplets and play a crucial role in regulating lipid turnover. Despite the significance of lipid metabolism for muscle contraction, the understanding of the specific roles of individual PLINs in skeletal muscle is limited. Pourteymour et al. ^51^ investigated the impact of extended exercise on the expression of PLINs. A group of 26 middle-aged sedentary men participated in a 12-week combined endurance and strength training program. Among the five PLINs studied, PLIN4 mRNA exhibited the highest expression levels vastus lateralis muscle and subcutaneous adipose tissue, and this expression was significantly reduced after the extended exercise regimen in skeletal muscle. Additionally, the expression of PLIN4 mRNA in muscle was found to be correlated with the expression of genes associated with the synthesis of phospholipids from scratch, as well as with the levels of phosphatidylethanolamine and phosphatidylcholine in the muscle, and with the quantity of lipid droplets located just beneath the muscle cell membrane. The PLIN4 protein predominantly localized to the outer regions of skeletal muscle fibers, with higher concentrations in slow-twitch fibers in comparison to fast-twitch muscle fibers. In summary, there is a decrease in PLIN4 expression following prolonged physical activity, with a preference for slow-twitch skeletal muscle fibers. Additionally, PLIN4 predominantly localizes near the plasma membrane of muscle cells ^51^. |
| B2 | Dual specificity mitogen-activated protein kinase kinase 3 (MP2K3) | MP2K3_HUMAN | MAP2K3 | Dual specificity mitogen-activated protein kinase kinase 3 (MP2K3) is a dual specificity protein kinase that belongs to the MAP kinase kinase family. This kinase is activated by cytokines and environmental stress and participates in the MAP kinase-mediated signaling cascade. It phosphorylates and thus activates MAPK14/p38-MAPK ^52,53^. It can be activated by insulin and is necessary for the expression of glucose transporter. |
| B2 | Nucleoredoxin  (NXN) | NXN_HUMAN | NXN | Nucleoredoxin (NXN), an enzyme with oxidoreductase activity, plays a crucial role in maintaining cellular redox balance by modulating various signaling pathways in response to changes in redox conditions. It participates in the control of numerous fundamental cellular processes, serving as a central hub for various redox-sensitive signaling pathways and related disorders ^54^.  NRX was demonstrated to function as a promoter of adipogenesis by influencing Wnt signaling, and it is linked to the physical signs of obesity. As a result, intervening with NRX to modulate Wnt/-catenin signaling might potentially serve as an efficient therapeutic approach for addressing obesity and metabolic conditions like diabetes ^55^. |
| B2 | Dehydrogenase/reductase SDR family member  (DRS7B) | DRS7B_HUMAN | DHRS7B | Dehydrogenase/reductase (SDR family) member 7 (DHRS7, also known as retSDR4 or SDR34C1) is a newly identified member of the short-chain dehydrogenase/reductase (SDR) superfamily. The SDR superfamily in humans is recognized for its significant involvement in diverse (patho)biochemical processes, encompassing intermediary metabolism and the transformation of foreign substances (xenobiotics) ^56^. |
| B2 | 26S proteasome regulatory subunit  (RPS7) (PRS7) | PRS7_HUMAN | PSMC2 | 40S ribosomal protein S7 (RPS7) is a component of the 40S ribosomal subunit and is therefore involved in translation.  Although the mechanism is not yet fully understood, mTOR may interact with eIF3, to regulate the association of eIF3 and eIF4G. Insulin increases eIF4E binding to eIF4G by enhancing 4EBP1 phosphorylation via mTOR signaling, resulting in more eIF4F bound to the mRNA's 5′ cap region. By promoting eIF4G binding to eIF3, it is expected that the joining of eIF4F and the 40S ribosome will be enhanced, thereby increasing the rate at which the small ribosomal subunit scans and positions itself on the mRNA to initiate translation ^46^. |
| B3 | Endothelial differentiation-related factor 1 (EDF1) | EDF1_HUMAN | EDF1 | Endothelial differentiation-related factor 1 (EDF1) is engaged in suppressing the differentiation of endothelial cells and is the first-known calmodulin (CaM)-binding protein in endothelial cells. Ballabio et al. ^57^ have observed that EDF-1 can be phosphorylated by protein kinase A (PKA) both *in vitro* and *in vivo*; the interaction between EDF-1 and CaM is influenced by the phosphorylation of EDF-1 by PKA; forskolin promotes the accumulation of EDF-1 in the cell nucleus; and PKA-mediated phosphorylation enhances the interaction of EDF-1 with the TATA-binding protein. CaM regulates the activity of various enzymes, including nitric oxide synthase (NOS). EDF-1, when not phosphorylated, inhibits the activity of NOS. Consequently, they have observed an increase in NOS activity in cells that express low levels of EDF-1. EDF-1 has two primary roles in endothelial cells: it controls the presence of CaM in the cytoplasm and it functions as a transcriptional coactivator in the cell nucleus ^57^. |
| B3 | Proteasome subunit alpha type-2  (PSA2) | PSA2_HUMAN | PSMA2 | Proteasome subunit alpha type-2 (PSA2) is a constituent of the 20S core proteasome complex, which is responsible for breaking down most intracellular proteins through enzymatic processes. This complex performs a wide range of crucial functions within the cell, primarily by interacting with various regulatory components. When it combines with two 19S regulatory particles, it forms the 26S proteasome, thereby participating in the energy-dependent degradation of proteins marked with ubiquitin. The 26S proteasome plays a pivotal role in upholding the equilibrium of cellular proteins by eliminating misfolded or damaged proteins that could hinder cellular operations, as well as disposing of proteins that are no longer necessary. When associated with PA200 or PA28, the 20S proteasome facilitates the degradation of proteins independently of ubiquitin. This type of protein degradation is essential in various pathways, including spermatogenesis when interacting with the 20S-PA200 complex or in the generation of a specific set of antigenic peptides presented by MHC class I molecules when in combination with the 20S-PA28 complex ^58^. |
| B3 | Eukaryotic translation initiation factor 1A, Y-chromosomal (IF1AY)  Eukaryotic translation initiation factor 1A, X-chromosomal (IF1AX) | IF1AY_HUMAN  IF1AX_HUMAN | EIF1AY  EIF1AX | The 17-kDa eukaryotic initiation factor, eIF1A (formerly referred to as eIF-4C) in mammals, is vital for the transfer of the initiator Met-tRNAf, along with eIF2 and GTP, to the 40 S ribosomal subunits, creating the 40 S preinitiation complex, even in the absence of mRNA. Furthermore, eIF1A plays a catalytic role in this process, ensuring the highly efficient transfer of the Met-tRNAf·eIF2·GTP complex to the 40 S ribosomal subunits. Notably, the resulting 40 S complex doesn't retain eIF1A, indicating that its function in forming the 40 S preinitiation complex isn't related to stabilizing the binding of Met-tRNAf to the 40 S ribosomes. Moreover, in the presence of an AUG codon, the eIF1A-mediated 40 S initiation complex effectively combines with 60 S ribosomal subunits in a process dependent on eIF5, resulting in the formation of a functional 80 S initiation complex. In contrast to some previous reports, eIF1A doesn't play a role in either the subunit joining reaction or the generation of ribosomal subunits from 80 S ribosomes. Instead, the primary function of eIF1A is to facilitate the transfer of Met-tRNAf to 40 S ribosomal subunits, ultimately leading to the formation of the 40 S preinitiation complex ^59^. |
| B3 | Ras-related protein R-Ras2 (RRAS2) | RRAS2_HUMAN | RRAS2 | Ras-related protein R-Ras2 (RRAS2) is a member of the Ras GTPase protein family, which are involved in the regulation of MAPK signaling pathway, thereby controlling multiple cellular processes (which are involved in the regulation of various cellular processes, including the MAPK signaling pathway).  Inhibition of Ras signaling has been shown to improve insulin sensitivity and glucose uptake both *in vitro* and *in vivo*. Beneficial effects of Ras inhibition were observed, leading to attenuation of hyperglycemia in the conventional model of T2D, indicating that targeting Ras signaling may be a promising approach for improving glucose homeostasis in diabetes (the treatment of insulin resistance and T2D ^60^. |
| B3 | Very-long-chain 3-oxoacyl-CoA reductase  (DHB12) | DHB12_HUMAN | HSD17B12 | Very-long-chain 3-oxoacyl-CoA reductase (DHB12) is responsible for facilitating the second step in the elongation cycle of long-chain fatty acids. This process occurs within the endoplasmic reticulum and enables the addition of two carbon units to the existing chain of both long-chain and very long-chain fatty acids (VLCFAs) during each cycle. Specifically, this enzyme exhibits 3-ketoacyl-CoA reductase activity, which involves the conversion of 3-ketoacyl-CoA into 3-hydroxyacyl-CoA within the context of fatty acid elongation. As a result, it plays a role in the generation of VLCFAs with various chain lengths, which are integral to multiple biological functions, serving as precursors for membrane lipids and lipid mediators ^61^. |
| B3 | Cytosolic 10-formyltetrahydrofolate dehydrogenase  (AL1L1) | AL1L1_HUMAN | ALDH1L1 | ALDH1L1 (10-formyltetrahydrofolate dehydrogenase), an enzyme involved in folate metabolism that is abundantly expressed in the liver, catalyzes the conversion of 10-formyltetrahydrofolate into tetrahydrofolate (THF). This reaction potentially plays a role in regulating the levels of reduced folate, the synthesis of purines de novo, and the flux of methyl groups bound to folate compounds ^62^.  The function of ALDH1L2 plays a crucial role in maintaining overall metabolic balance, which has broader implications for various human diseases. For instance, an increase in plasma levels of C3 and C5 acylcarnitines, associated with the loss of ALDH1L2, could be linked to conditions like obesity, metabolic syndrome, insulin resistance, and T2D. This rise in acylcarnitines may be a consequence of impaired mitochondrial function or dysfunction. Furthermore, higher concentrations of butyrylcarnitine and methylbutyrylcarnitine were observed in individuals with non-alcoholic steatohepatitis (NASH). Additionally, variations in plasma acylcarnitine levels are indicative of the boundary between health and disease in cases of metabolic syndrome, especially among middle-aged Dutch individuals.  The role of ALDH1L2 in regulating mitochondrial function, beta-oxidation, and acylcarnitine levels underscores its potential relevance to diabetes. Elevated plasma acylcarnitines have been identified as predictive markers for mitochondrial dysfunction in T2D, possibly associated with incomplete beta-oxidation of fatty acids. Mechanistically, an overload of mitochondria could generate by-products that affect the insulin signaling pathway, leading to insulin resistance, as evidenced by altered mitochondrial beta-oxidation in patients with T2D. These alterations are likely exacerbated in obese patients, with T2D patients showing increased levels of short-chain acylcarnitines (C2 and C4). Elevated plasma levels of short-chain acylcarnitines have also been linked to a higher risk of gestational diabetes mellitus and identified as predictive markers for the development of diabetic nephropathy.  While further research is needed to fully understand the role of ALDH1L2 in diabetes, the changes in multiple metabolites observed in Aldh1l2 knockout mice in this study suggest a connection between Aldh1l2 and diabetes. This connection is supported by alterations in the levels of end-products of protein glycation, the glucose marker 1,5-anhydroglucitol, and 3-methylhistidine in Aldh1l2 knockout mice ^110^. |
| B3 | Ankyrin-3  (ANK3) | ANK3_HUMAN | ANK3 | Ankyrins are adaptor molecules found in eukaryotic cells, which interact with ion channel proteins, cell adhesion molecules, signaling molecules, and cytoskeleton components. They serve as crucial scaffolding proteins involved in anchoring to the muscle membrane, contributing to muscle development, neurogenesis, and synapse formation.  ANK R is predominantly found in red blood cells. ANK B, also known as Ankyrin-2 and Brain ankyrin is abundantly expressed in the nervous system. ANK G encoded by ank3 expressed in most human tissues ^63^.  Functional variants of ankyrin-B have been linked to certain human diseases, including hereditary cardiac arrhythmia and T2D ^111^.  The introduction of human mutations in the protein ankyrin-2 (AnkB) in mice leads to several outcomes, including abnormalities in the pancreas, increased susceptibility to weight gain, and eventual development of obesity and insulin resistance. It has been found that mice become significantly fatter a nd enter a pre-diabetic state when AnkB is eliminated specifically from fat cells. Deficiencies in AnkB lead to abnormalities in the cellular uptake of glucose by both fat cells and muscles.  Lorenzo & Bennett proposed a theory that adiposity, or increased fat accumulation, can be attributed to a cell-autonomous mechanism. It arises from a persistent presence of glucose transporter type 4 (GLUT4) on the cell surface of adipocytes, which is caused by the impaired function of ankyrin-B (AnkB) in coupling GLUT4 to clathrin-mediated endocytosis. Mice with adipose tissue-specific AnkB knockout (AnkB-KO) exhibit obesity and progressive dysfunction of pancreatic islets with age or high-fat diet (HFD). Adipocytes lacking AnkB display elevated lipid accumulation due to increased glucose uptake and impaired endocytosis of GLUT4. In adipocytes, AnkB directly interacts with GLUT4 and clathrin, promoting their association ^64^. |
| C1 | Serum amyloid P-component (SAMP) | SAMP_HUMAN | APCS | Serum Amyloid P is a member of the pentraxin protein family, is secreted by liver cells, and plays crucial roles in the immune response and inflammation. In cases of systemic amyloidosis, where amyloid deposits accumulate in various tissues, Serum Amyloid P is consistently found attached to these amyloid fibrils, making it more readily detectable than another related protein called Serum Amyloid A. Consequently, Serum Amyloid P could be a valuable biomarker and a potential target for treating obesity and the related amyloidosis ^65^. |
| C1 | Eukaryotic translation initiation factor 3 subunit F (EIF3F) | EIF3F_HUMAN | EIF3F | The eukaryotic initiation factor 3 (eIF3) is among the most intricate translation initiation factors in mammalian cells, comprising multiple subunits, from eIF3a to eIF3m. It plays a vital role in translation initiation, termination, and ribosomal recycling. Consequently, abnormal eIF3 expression is linked to various pathological conditions. A profound comprehension of the molecular mechanisms governing the role of eIF3 in human diseases is crucial for the development of novel therapeutic strategies targeting eIF3, with the aim of addressing these conditions effectively ^66^. |
| C1 | Proteasome subunit alpha type-6 (PSA6) | PSA6_HUMAN | PSMA6 | Proteasome subunit alpha type-6 (PSA6) is a constituent of the 20S core proteasome complex, which is responsible for breaking down most intracellular proteins through enzymatic processes. This complex performs a wide range of crucial functions within the cell, primarily by interacting with various regulatory components. When it combines with two 19S regulatory particles, it forms the 26S proteasome, thereby participating in the energy-dependent degradation of proteins marked with ubiquitin. The 26S proteasome plays a pivotal role in upholding the equilibrium of cellular proteins by eliminating misfolded or damaged proteins that could hinder cellular operations, as well as disposing of proteins that are no longer necessary. When associated with PA200 or PA28, the 20S proteasome facilitates the degradation of proteins independently of ubiquitin. This type of protein degradation is essential in various pathways, including spermatogenesis when interacting with the 20S-PA200 complex or in the generation of a specific set of antigenic peptides presented by MHC class I molecules when in combination with the 20S-PA28 complex ^58^. |
| C1 | Apolipoprotein E  (ApoE) | APOE_HUMAN | APOE | Apolipoprotein E (ApoE) is a crucial protein that plays a central role in maintaining lipid balance, as it regulates the metabolism of cholesterol, triglycerides, and phospholipids in both the bloodstream and the brain ^67^. One of its primary functions is to facilitate the binding of lipoproteins or lipid complexes in the plasma or interstitial fluids to specific cell-surface receptors. These receptors enable the internalization of lipoprotein particles containing ApoE, thereby participating in the distribution and redistribution of lipids across various tissues and cells in the body. Furthermore, ApoE, when found within cells, can modulate a range of physiological and pathophysiological cellular processes. These processes include influencing cytoskeletal assembly and stability, maintaining mitochondrial integrity and function, and impacting dendritic morphology and function ^112^.  Kawashima et al. ^113^ showed that the absence of ApoE eliminates insulin resistance in a mouse model of T2D. This implies that the primary factor contributing to insulin resistance in this mouse model is the buildup of lipids in the tissues ^113^. |
| C2, C3 | Very long-chain specific acyl-CoA dehydrogenase, mitochondrial  (ACADV) | ACADV_HUMAN | ACADVL | Very long-chain specific acyl-CoA dehydrogenase, mitochondrial (ACADV) is an enzyme belonging to the group of acyl-CoA dehydrogenases responsible for initiating the initial step of mitochondrial fatty acid beta-oxidation. This process, carried out aerobically, breaks down fatty acids into acetyl-CoA, enabling the generation of energy from fats ^114,115^.  Mitochondrial dysfunction with impaired skeletal muscle oxidative phosphorylation has been linked to the development of insulin resistance and T2D. In mice, a deficiency in long-chain acyl-CoA dehydrogenase (LCAD) resulted in mitochondrial dysfunction, causing fatty liver and hepatic insulin resistance. A closely related enzyme within the same metabolic pathway, known as very long chain acyl-CoA dehydrogenase (VLCAD), is an enzyme associated with the mitochondrial membrane that precedes long chain acyl-CoA dehydrogenase in the mitochondrial fatty acid beta-oxidation process. Previous research in humans has found that VLCAD deficiency leads to decreased fatty acid oxidation and is associated with symptoms such as fasting intolerance, a disease resembling Reye syndrome, and conditions affecting the heart and skeletal muscle. VLCAD−/− mice were found to be protected from diet-induced obesity and insulin resistance. This protection is attributed to the persistent activation of AMPK (in the liver and muscle) and PPARα (in muscle and brown adipose tissue), leading to elevated fatty acid oxidation and reduced levels of diacylglycerol within muscle and liver cells. Additionally, these results underscore the paradoxical outcome of heightened insulin sensitivity as a consequence of compensatory mechanisms triggered by mitochondrial dysfunction ^68^. |
| C2, C3 | Transmembrane protein 11, mitochondrial  (TMM11) | TMM11_HUMAN | TMEM11 | Mitochondria play vital roles in cellular metabolism and require regulation through various quality control pathways, including mitophagy, to maintain their integrity. In BNIP3/BNIP3L-dependent receptor-mediated mitophagy, mitochondria are selectively earmarked for degradation by directly engaging the autophagy protein LC3. Situational upregulation of BNIP3 and/or BNIP3L occurs, such as during hypoxia and erythrocyte maturation in development. Gok M.O. et al revealed that the relatively less understood mitochondrial protein TMEM11 forms a complex with BNIP3 and BNIP3L and co-localizes at sites where mitophagosomes are formed. They have observed that mitophagy is more active in the absence of TMEM11 under both normoxia and hypoxia-mimicking conditions, primarily because there is an increase in the number of BNIP3/BNIP3L mitophagy sites. This supports a model in which TMEM11 plays a role in restricting the spatial formation of mitophagosomes ^69^. |
| C2 | Laminin subunit beta-1 (LAMB1) | LAMB1_HUMAN | LAMB1 | Laminin, a crucial constituent of the extracellular matrix (ECM), ranks among the largest non-collagenous glycoproteins present in the basement membrane. (Laminin, a substantial glycoprotein found in the basement membrane, is a vital element of the extracellular matrix.)  The extracellular matrix (ECM) elements, especially laminin, have been identified as significant controllers of insulin activity within muscle, liver, and adipose tissue. Altun et al. indicated that serum laminin levels are lower in patients with insulin resistance possibly by regulating the insulin effect through integrins (that individuals with insulin resistance tend to have reduced serum levels of laminin, which may be influencing insulin's actions via integrin regulation). Targeting laminin may be beneficial for pathogenesis and associated complications of insulin resistance ^70^. |
| C2 | Lon protease homolog, mitochondrial (LONM) | LONM_HUMAN | LONP1 | LONP1, a member of the highly conserved AAA+ superfamily, plays a critical role in maintaining protein quality within the mitochondrial matrix by breaking down misfolded or oxidized polypeptides. It is also a key component of the UPRmt. The connection between LONP1 and metabolic disorders has been established through several findings, including the observation of low levels of LONP1 expression in the livers of diabetic db/db mice. Additionally, reducing LONP1 expression has been shown to lead to impaired insulin signaling and an increase in the expression of gluconeogenic enzymes in human liver cells. J. H. Lee et al. discovered that mitochondrial LONP1, a protein responsible for maintaining mitochondrial health when the body is under stress, exhibited varying levels of activity in visceral adipose tissue (VAT) depending on an individual's BMI. Furthermore, an analysis using bioinformatics revealed that elevated LONP1 levels in VAT were linked to improvements in glucose and lipid metabolism ^71^. |
| C2 | Ubiquitin carboxyl-terminal hydrolase isozyme L1 (UCHL1) | UCHL1_HUMAN | UCHL1 | Ubiquitin carboxyl-terminal hydrolase isozyme L1 UCHL1 enzyme is a thiol protease engaged both in the processing of ubiquitin precursors and of ubiquitinated proteins. Ubiquitin C-terminal hydrolase L1 (UCH-L1) is an abundantly expressed protein in the brain, specifically in the neurons. Besides, UCH-L1 has very limited expression in other healthy tissues, but it shows high expression in several cancer forms. UCH-L1 is not essential for neuronal development, but it is absolutely necessary for maintaining axonal integrity, and dysfunction of UCH-L1 is linked to neurodegenerative diseases ^72^. |
| C2 | Interferon-inducible double-stranded RNA-dependent protein kinase activator A  (PRKRA) | PRKRA_HUMAN | PRKRA | Chronic inflammation is a prominent feature of obesity, making it crucial to investigate pathways that link nutrient and pathogen sensing mechanisms. This understanding is vital for unraveling the mechanisms behind insulin resistance, T2D, and other persistent metabolic disorders. Nakamura et al. have presented evidence suggesting that the double-stranded RNA-dependent protein kinase (PKR) can not only respond to nutrient signals but also to endoplasmic reticulum (ER) stress. It coordinates the actions of other important inflammatory kinases, such as c-Jun N-terminal kinase (JNK), to regulate insulin function and metabolism. Additionally, PKR directly interacts with and modifies the insulin receptor substrate, effectively integrating nutrient signals and insulin responses with a defined pathogen response system. In cases of dietary and genetic obesity, PKR is significantly activated in adipose and liver tissues. Remarkably, the absence of PKR alleviates metabolic deterioration resulting from excess nutrients or energy in mice. These findings underscore the critical role of PKR as an integral component of an inflammatory complex that responds to nutrient signals and organelle dysfunction ^73^. |
| C2 | Cocaine esterase  (EST2) | EST2_HUMAN | CES2 | Cocaine esterase (EST2) participates in the detoxification of xenobiotics and facilitates the activation of ester and amide prodrugs ^74^. Additionally, it transforms monoacylglycerides into free fatty acids and glycerol, and carries out the hydrolysis of 2-arachidonoylglycerol and prostaglandins ^116^. |
| C2 | Acetyl-coenzyme A synthetase 2-like, mitochondrial  (ACS2L) | ACS2L_HUMAN | ACSS1 | Acetyl-CoA synthetase 2 (ACSS2), a significant member of the acetyl-CoA synthetase (ACSS) family, has the capability to convert acetate into acetyl coenzyme A (acetyl-CoA). Currently, acetyl-CoA is recognized as a crucial intermediate metabolite in the energy substrate metabolism. Moreover, various nutrients converge into a common metabolic pathway, the tricarboxylic acid cycle and oxidative phosphorylation, through acetyl-CoA. ACSS2 not only plays a pivotal role in material energy metabolism but also contributes to the regulation of several acetylation processes, including the control of histone and transcription factor acetylation ^75^.  ACSS2 possesses a dual role, serving as both an enzyme involved in adipogenesis and a regulator of the stress response. Its specific function depends on the nutritional and stress conditions. In typical circumstances when there is no injury or stress, ACSS2 primarily functions as a cytoplasmic enzyme responsible for promoting the synthesis and storage of lipids. However, in situations characterized by nutrient scarcity, stress, or injury, ACSS2 transitions to a regulatory role, promoting fatty acid oxidation and autophagy to uphold energy balance ^75^. |
| C3 | Glutathione S-transferase theta-1 (GSTT1) | GSTT1_HUMAN | GSTT1 | Glutathione S-transferase theta-1 binds reduced glutathione to a diverse range of hydrophobic electrophiles, both originating from external sources and within the body. It also exhibits glutathione peroxidase function when interacting with cumene hydroperoxide ^117,118^. Hepatic glutathione S-transferases (GSTs) experience abnormal regulation in conditions such as human obesity, nonalcoholic fatty liver disease, and diabetes. The versatile GST pi-isoform (GSTP) is responsible for facilitating the binding of glutathione to acrolein and also acts to block the activation of c-Jun NH2-terminal kinase (JNK) ^76^. |
| C3 | Thioredoxin-like protein 1 (TXNL1) | TXNL1_HUMAN | TXNL1 | Thioredoxin-like protein 1 (TXNL1), a member of the thioredoxin superfamily known for its redox-regulating capabilities, plays a crucial role in maintaining cell viability through various antioxidant and anti-apoptotic mechanisms. While the link between insulin resistance and oxidative stress is well-established, the specific role of TXNL1 in T2D remains an area that has not been extensively investigated ^78^.  Thioredoxin-interacting protein (TXNIP) is a factor that regulates the generation and loss of pancreatic β-cells. TXNIP, an α-arrestin, can bind to and inhibit thioredoxin, the antioxidant protein produced in pancreatic islets following glucose intake. Numerous studies have demonstrated that elevated TXNIP levels lead to β-cell apoptosis, while TXNIP deficiency protects against both type I and type II diabetes by promoting β-cell survival. Currently, depleting TXNIP is emerging as a crucial factor in enhancing pancreatic β-cell survival. Targeting TXNIP may serve as a unique therapeutic opportunity, not only for improving insulin secretion and sensitivity but also for ameliorating the long-term microvascular and macrovascular complications of the disease. Consequently, inhibitors of TXNIP that can reduce its expression and/or activity to non-diabetic levels are promising agents to curb the increasing prevalence of diabetes and its associated complications ^77^. |
| C3 | Ras-related protein Rab-5B (RAB5B) | RAB5B_HUMAN | RAB5B | Ras-related protein Rab-5B (RAB5B) is a member of the Ras GTPase protein family, which are involved in the regulation of MAPK signaling pathway, thereby controlling multiple cellular processes. Inhibition of Ras signaling has been shown to improve insulin sensitivity and glucose uptake both *in vitro* and *in vivo*. Beneficial effects of Ras inhibition were observed, leading to attenuation of hyperglycemia in the conventional model of T2D, indicating that targeting Ras signaling may be a promising approach for improving glucose homeostasis in diabetes ^60^. |
| C3 | Isocitrate dehydrogenase [NADP] cytoplasmic  (IDHC) | IDHC_HUMAN | IDH1 | NADPH serves as a crucial cofactor in various enzymatic reactions, playing a key role in processes such as glutathione metabolism, as well as the synthesis of fats and cholesterol. Recent research has highlighted the significance of mitochondrial NADP(+)-dependent isocitrate dehydrogenase (IDPc) in cellular defense against oxidative damage. This is achieved by facilitating the production of NADPH, essential for the regeneration of reduced glutathione. IDPc is particularly critical in the biosynthesis of fats and cholesterol.  During the differentiation of 3T3-L1 adipocytes, both the enzymatic activity and protein content of IDPc increased in a time-dependent manner. Augmented expression of IDPc through stable transfection of its cDNA was positively associated with the adipogenesis of 3T3-L1 cells. Conversely, decreased expression of IDPc using an antisense IDPc vector hindered adipogenesis. In transgenic mice with heightened IDPc expression, features such as fatty liver, hyperlipidemia, and obesity were observed.  In the epididymal fat pads of these transgenic mice, there was a notable increase in the expression of adipocyte-specific genes, including peroxisome proliferator-activated receptor gamma. Additionally, the hepatic and epididymal fat pad contents of acetyl-CoA and malonyl-CoA were significantly lower in the transgenic mice, while the total triglyceride and cholesterol contents were notably higher in the liver and serum compared to wild-type mice. This suggests that the elevated IDPc activity accelerates the consumption rate of lipogenic precursors required for fat biosynthesis. In summary, IDPc is a major NADPH producer crucial for the synthesis of fats and cholesterol ^79^. |
| C3 | Mth938 domain-containing protein  (AAMDC) | AAMDC_HUMAN | AAMDC | Adipogenesis Associated Mth938 Domain-Containing Protein (AAMDC) is an uncharacterized oncogene that undergoes amplification in aggressive estrogen receptor-positive breast cancers. AAMDC plays a role in regulating the expression of various metabolic enzymes associated with the one-carbon folate and methionine cycles, as well as lipid metabolism. AAMDC is demonstrated to influence PI3K-AKT-mTOR signaling, thereby controlling the translation of ATF4 and MYC and modulating the transcriptional activity of AAMDC-dependent promoters. Elevated AAMDC expression correlates with increased sensitivity to dactolisib and everolimus, with these PI3K-mTOR inhibitors displaying synergistic interactions with anti-estrogens in IntClust2 models.  Ectopic expression of AAMDC alone is sufficient to activate AKT signaling, leading to estrogen-independent tumor growth. Consequently, tumors overexpressing AAMDC may exhibit sensitivity to a combination of PI3K-mTORC1 blockers and anti-estrogens. Lastly, AAMDC can interact with RabGTPase-activating protein RabGAP1L, and that AAMDC, RabGAP1L, and Rab7a colocalize in endolysosomes. The identification of the RabGAP1L-AAMDC assembly platform offers valuable insights for designing selective blockers targeting malignancies characterized by AAMDC amplification ^80^. |
| C3 | Glycogen debranching enzyme  (GDE) | GDE_HUMAN | AGL | Glycogen debranching enzyme (GDE) serves as a versatile enzyme with dual functions, acting both as a 1,4-alpha-D-glucan:1,4-alpha-D-glucan 4-alpha-D-glycosyltransferase and an amylo-1,6-glucosidase in the process of glycogen degradation.  In the context of T2D, increased levels of plasma glucose contribute to dysfunction and death of pancreatic β cells. The accumulation of glycogen, resulting from impaired metabolism, plays a role in this "glucotoxicity" by disrupting biochemical pathways that contribute to β cell dysfunction.  It is noteworthy that under normal glucose conditions, β cells do not store glycogen. However, they can accumulate significant amounts in response to hyperglycemia, requiring a combination of stressors: elevated plasma glucose (or G-6-P), upregulation of PTG, and impaired oxidative metabolism ^81^. |
| C3 | Dolichyl-diphosphooligosaccharide--protein glycosyltransferase subunit 2  (RPN2) | RPN2_HUMAN | RPN2 | Dolichyl-diphosphooligosaccharide--protein glycosyltransferase subunit 2 (RPN2) is a part of the oligosaccharyltransferase complex responsible for transferring high-mannose oligosaccharides to asparagine residues on newly forming polypeptides within the rough endoplasmic reticulum. This protein complex is consistently isolated alongside ribosomes. Additionally, it is involved in processing advanced glycation endproducts (AGEs), which result from non-enzymatic interactions between sugars and proteins or lipids. These AGEs are linked to aging and elevated blood sugar levels. |
| C3 | T-complex protein 1 subunit delta  (TCPD) | TCPD_HUMAN | CCT4 | T-complex protein 1 subunit delta (TCPD) is part of the chaperonin-containing T-complex (TRiC), a molecular chaperone complex facilitating protein folding through ATP hydrolysis. The TRiC complex likely participates in the folding processes of actin and tubulin ^82,83^. |

# Appendix D: Proteins identified by the REHA algorithm in Figure 2

The table in Appendix D enriches Figure 2 by providing further details on proteins identified by the REHA algorithm as significant for classification tasks between tested groups. It contains a detailed list that includes the comparison groups involved, the occurrence frequency of each protein within the study, a description of each protein, its unique UniProtKB identifier, and the gene name associated with each protein.

| **Comparison** | **Occurence** | | **Protein description** | | **UniProtKB identifier** | | **Gene name** |
| --- | --- | --- | --- | --- | --- | --- | --- |
| **PD-NG** | 23 | | 14-3-3 protein eta | | 1433F_HUMAN | | YWHAH |
|  | 23 | | Eukaryotic translation initiation factor 4B | | IF4B_HUMAN | | EIF4B |
|  | 19 | | Adenylate kinase 2, mitochondrial | | KAD2_HUMAN | | AK2 |
|  | 18 | | HIG1 domain family member 1A, mitochondrial | | HIG1A_HUMAN | | HIGD1A |
|  | 13 | | Laminin subunit beta-2 | | LAMB2_HUMAN | | LAMB2 |
|  | 13 | | Serine/threonine-protein kinase OSR1 | | OXSR1_HUMAN | | OXSR1 |
|  | 9 | | MICOS complex subunit MIC13 | | MIC13_HUMAN | | MICOS13 |
|  | 8 | | Protein-L-isoaspartate(D-aspartate) O-methyltransferase | | PIMT_HUMAN | | PCMT1 |
|  | 8 | | Dehydrogenase/reductase SDR family member 7C | | DRS7C_HUMAN | | DHRS7C |
|  | 7 | | Branched-chain-amino-acid aminotransferase, mitochondrial | | BCAT2_HUMAN | | BCAT2 |
|  | 7 | | Inositol monophosphatase 2 | | IMPA2_HUMAN | | IMPA2 |
|  | 6 | | Epoxide hydrolase 1 | | HYEP_HUMAN | | EPHX1 |
|  | 6 | | Tubulin beta-6 chain | | TBB6_HUMAN | | TUBB6 |
|  | 5 | | Bifunctional glutamate/proline--tRNA ligase | | SYEP_HUMAN | | EPRS1 |
|  | 5 | | 60S ribosomal protein L30 | | RL30_HUMAN | | RPL30 |
|  | 5 | | Creatine kinase B-type | | KCRB_HUMAN | | CKB |
|  | 5 | | Short-chain specific acyl-CoA dehydrogenase, mitochondrial | | ACADS_HUMAN | | ACADS |
|  | 4 | | Small nuclear ribonucleoprotein-associated proteins B and  B';Small nuclear ribonucleoprotein-associated protein N | | RSMB_HUMAN  RSMN_HUMAN | | SNRPB  SNRPN |
|  | 4 | | Mitogen-activated protein kinase 1 | | MK01_HUMAN | | MAPK1 |
|  | 4 | | 26S proteasome regulatory subunit 6B | | PRS6B_HUMAN | | PSMC4 |
|  | 3 | | Caveolae-associated protein 4 | | CAVN4_HUMAN | | CAVIN4 |
|  | 3 | | ATP synthase subunit d, mitochondrial | | ATP5H_HUMAN | | ATP5PD |
|  | 3 | | Methylcrotonoyl-CoA carboxylase beta chain, mitochondrial | | MCCB_HUMAN | | MCCC2 |
|  | 3 | | ATP-dependent (S)-NAD(P)H-hydrate dehydratase | | NNRD_HUMAN | | NAXD |
|  | 3 | | Serine/threonine-protein kinase Nek7 | | NEK7_HUMAN | | NEK7 |
|  | 3 | | Glycerol-3-phosphate dehydrogenase [NAD(+)], cytoplasmic | | GPDA_HUMAN | | GPD1 |
|  | 3 | | Aldo-keto reductase family 1 member B1 | | ALDR_HUMAN | | AKR1B1 |
|  | 3 | | GTP-binding protein SAR1b | | SAR1B_HUMAN | | SAR1B |
|  | 3 | | Programmed cell death protein 5 | | PDCD5_HUMAN | | PDCD5 |
|  | 3 | | Dynamin-1-like protein | | DNM1L_HUMAN | | DNM1L |
|  | 3 | | Pterin-4-alpha-carbinolamine dehydratase | | PHS_HUMAN | | PCBD1 |
|  | 3 | | Leiomodin-3 | | LMOD3_HUMAN | | LMOD3 |
|  | 3 | | Protein CutA | | CUTA_HUMAN | | CUTA |
|  | 3 | | Pyruvate dehydrogenase protein X component, mitochondrial | | ODPX_HUMAN | | PDHX |
|  | 2 | | Proline-rich basic protein 1 | | PROB1_HUMAN | | PROB1 |
|  | 2 | | Mitochondrial coenzyme A transporter SLC25A42 | | S2542_HUMAN | | SLC25A42 |
| **Comparison** | | **Occurence** | | **Protein description** | | **UniProtKB identifier** | **Gene name** |
| **T2D-NG** | | 23 | | Pterin-4-alpha-carbinolamine dehydratase | | PHS_HUMAN | PCBD1 |
|  |  | 20 | | Thioredoxin reductase 1, cytoplasmic | | TRXR1_HUMAN | TXNRD1 |
|  |  | 13 | | Fumarylacetoacetase | | FAAA_HUMAN | FAH |
|  |  | 11 | | Mitochondrial coenzyme A transporter SLC25A42 | | S2542_HUMAN | SLC25A42 |
|  |  | 9 | | Perilipin-4 | | PLIN4_HUMAN | PLIN4 |
|  |  | 9 | | Methyltransferase-like protein 7A | | MET7A_HUMAN | METTL7A |
|  |  | 8 | | EH domain-binding protein 1-like protein 1 | | EH1L1_HUMAN | EHBP1L1 |
|  |  | 7 | | Ryanodine receptor 1 | | RYR1_HUMAN | RYR1 |
|  |  | 6 | | Cullin-associated NEDD8-dissociated protein 2 | | CAND2_HUMAN | CAND2 |
|  |  | 6 | | Endothelial differentiation-related factor 1 | | EDF1_HUMAN | EDF1 |
|  |  | 6 | | Heterogeneous nuclear ribonucleoprotein C-like 1;  Heterogeneous nuclear ribonucleoprotein C-like 2;  Heterogeneous nuclear ribonucleoprotein C-like 3;  Heterogeneous nuclear ribonucleoprotein C-like 4;  Heterogeneous nuclear ribonucleoproteins C1/C2 | | HNRC1_HUMAN  HNRC2_HUMAN  HNRC3_HUMAN  HNRC4_HUMAN  HNRPC_HUMAN | HNRNPCL1  HNRNPCL2  HNRNPCL3  HNRNPCL1  HNRNPC |
|  |  | 5 | | Fructosamine-3-kinase | | FN3K_HUMAN | FN3K |
|  |  | 5 | | Programmed cell death protein 5 | | PDCD5_HUMAN | PDCD5 |
|  |  | 5 | | Ras-related protein Rab-5B | | RAB5B_HUMAN | RAB5B |
|  |  | 5 | | Glutamine amidotransferase-like class 1 domain-containing protein 3B, mitochondrial;  Glutamine amidotransferase-like class 1 domain-containing protein 3A, mitochondrial | | GAL3B_HUMAN  GAL3A_HUMAN | GATD3B  GATD3A |
|  |  | 5 | | Alanine aminotransferase 1 | | ALAT1_HUMAN | GPT |
|  |  | 5 | | Isopentenyl-diphosphate delta-isomerase 2 | | IDI2_HUMAN | IDI2 |
|  |  | 5 | | Ras-related protein R-Ras2 | | RRAS2_HUMAN | RRAS2 |
|  |  | 5 | | 40S ribosomal protein S6 | | RS6_HUMAN | RPS6 |
|  |  | 5 | | Aldehyde dehydrogenase, mitochondrial | | ALDH2_HUMAN | ALDH2 |
|  |  | 5 | | Proteasome subunit alpha type-2 | | PSA2_HUMAN | PSMA2 |
|  |  | 5 | | Ankyrin-3 | | ANK3_HUMAN | ANK3 |
|  |  | 5 | | Nucleoredoxin | | NXN_HUMAN | NXN |
|  |  | 5 | | Dual specificity mitogen-activated protein kinase kinase 3 | | MP2K3_HUMAN | MAP2K3 |
|  |  | 5 | | Branched-chain-amino-acid aminotransferase, mitochondrial | | BCAT2_HUMAN | BCAT2 |
|  |  | 4 | | Charged multivesicular body protein 4b | | CHM4B_HUMAN | CHMP4B |
|  |  | 4 | | NADH dehydrogenase [ubiquinone] iron-sulfur protein 8, mitochondrial | | NDUS8_HUMAN | NDUFS8 |
|  |  | 4 | | 26S proteasome regulatory subunit 7 | | PRS7_HUMAN | PSMC2 |
|  |  | 4 | | Proline-rich basic protein 1 | | PROB1_HUMAN | PROB1 |
|  |  | 4 | | Electrogenic aspartate/glutamate antiporter SLC25A12, mitochondrial | | S2512_HUMAN | SLC25A12 |
|  |  | 4 | | Smoothelin-like protein 1 | | SMTL1_HUMAN | SMTNL1 |
|  |  | 3 | | PDZ and LIM domain protein 1 | | PDLI1_HUMAN | PDLIM1 |
|  |  | 3 | | Synaptophysin-like protein 2 | | SYPL2_HUMAN | SYPL2 |
|  |  | 3 | | DNA-(apurinic or apyrimidinic site) endonuclease | | APEX1_HUMAN | APEX1 |
|  |  | 3 | | NADH dehydrogenase [ubiquinone] 1 beta subcomplex subunit 6 | | NDUB6_HUMAN | NDUFB6 |
|  |  | 3 | | 60S ribosomal protein L18 | | RL18_HUMAN | RPL18 |

| **Comparison** | **Occurence** | **Protein description** | **UniProtKB identifier** | **Gene name** |
| --- | --- | --- | --- | --- |
| **T2D-PD** | 17 | Ubiquitin carboxyl-terminal hydrolase isozyme L1 | UCHL1_HUMAN | UCHL1 |
|  | 17 | Caveolae-associated protein 1 | CAVN1_HUMAN | CAVIN1 |
|  | 16 | 26S proteasome non-ATPase regulatory subunit 11 | PSD11_HUMAN | PSMD11 |
|  | 15 | Glycogen [starch] synthase, muscle | GYS1_HUMAN | GYS1 |
|  | 12 | Fatty acid-binding protein, adipocyte | FABP4_HUMAN | FABP4 |
|  | 12 | Apolipoprotein C-I | APOC1_HUMAN | APOC1 |
|  | 11 | Apolipoprotein E | APOE_HUMAN | APOE |
|  | 11 | Proteasome subunit alpha type-6 | PSA6_HUMAN | PSMA6 |
|  | 7 | 40S ribosomal protein S5 | RS5_HUMAN | RPS5 |
|  | 7 | Cocaine esterase | EST2_HUMAN | CES2 |
|  | 7 | Acetyl-coenzyme A synthetase 2-like, mitochondrial | ACS2L_HUMAN | ACSS1 |
|  | 7 | High mobility group protein B1 | HMGB1_HUMAN | HMGB1 |
|  | 7 | Serum amyloid P-component | SAMP_HUMAN | APCS |
|  | 6 | Fumarylacetoacetase | FAAA_HUMAN | FAH |
|  | 6 | Elongation factor 1-delta | EF1D_HUMAN | EEF1D |
|  | 6 | Inositol monophosphatase 1 | IMPA1_HUMAN | IMPA1 |
|  | 6 | 60S ribosomal protein L13 | RL13_HUMAN | RPL13 |
|  | 6 | Glutathione S-transferase theta-1 | GSTT1_HUMAN | GSTT1 |
|  | 5 | ATP synthase subunit delta, mitochondrial | ATPD_HUMAN | ATP5F1D |
|  | 5 | Ceruloplasmin | CERU_HUMAN | CP |
|  | 5 | 6-phosphogluconate dehydrogenase, decarboxylating | 6PGD_HUMAN | PGD |
|  | 5 | Thioredoxin-like protein 1 | TXNL1_HUMAN | TXNL1 |
|  | 5 | Laminin subunit beta-1 | LAMB1_HUMAN | LAMB1 |
|  | 5 | Ras-related protein Rab-5B | RAB5B_HUMAN | RAB5B |
|  | 5 | Mth938 domain-containing protein | AAMDC_HUMAN | AAMDC |
|  | 5 | Very long-chain specific acyl-CoA dehydrogenase, mitochondrial | ACADV_HUMAN | ACADVL |
|  | 5 | Lon protease homolog, mitochondrial | LONM_HUMAN | LONP1 |
|  | 5 | Eukaryotic translation initiation factor 3 subunit F | EIF3F_HUMAN | EIF3F |
|  | 5 | Glycogen debranching enzyme | GDE_HUMAN | AGL |
|  | 5 | Dolichyl-diphosphooligosaccharide--protein glycosyltransferase subunit 2 | RPN2_HUMAN | RPN2 |
|  | 4 | Gelsolin | GELS_HUMAN | GSN |
|  | 4 | 60S ribosomal protein L24 | RL24_HUMAN | RPL24 |
|  | 4 | Eukaryotic translation initiation factor 2 subunit 1 | IF2A_HUMAN | EIF2S1 |
|  | 4 | NADH dehydrogenase [ubiquinone] 1 beta subcomplex subunit 3 | NDUB3_HUMAN | NDUFB3 |
|  | 4 | 40S ribosomal protein S12 | RS12_HUMAN | RPS12 |
|  | 3 | Protein-cysteine N-palmitoyltransferase HHAT-like protein | HHATL_HUMAN | HHATL |
|  | 3 | Perilipin-4 | PLIN4_HUMAN | PLIN4 |
|  | 3 | Methylmalonyl-CoA epimerase, mitochondrial | MCEE_HUMAN | MCEE |
|  | 3 | 40S ribosomal protein S6 | RS6_HUMAN | RPS6 |
|  | 3 | Transmembrane protein 11, mitochondrial | TMM11_HUMAN | TMEM11 |
|  | 2 | F-actin-capping protein subunit alpha-2 | CAZA2_HUMAN | CAPZA2 |
|  | 2 | Interferon-inducible double-stranded RNA-dependent protein kinase activator A | PRKRA_HUMAN | PRKRA |
| **PD- NG, T2D- NG** | 26 | Pterin-4-alpha-carbinolamine dehydratase | PHS_HUMAN | PCBD1 |
|  | 12 | Branched-chain-amino-acid aminotransferase, mitochondrial | BCAT2_HUMAN | BCAT2 |
|  | 13 | Mitochondrial coenzyme A transporter SLC25A42 | S2542_HUMAN | SLC25A42 |
|  | 8 | Programmed cell death protein 5 | PDCD5_HUMAN | PDCD5 |
|  | 6 | Proline-rich basic protein 1 | PROB1_HUMAN | PROB1 |
| **T2D0NG, T2D-PD** | 19 | Fumarylacetoacetase | FAAA_HUMAN | FAH |
|  | 12 | Perilipin-4 | PLIN4_HUMAN | PLIN4 |
|  | 10 | Ras-related protein Rab-5B | RAB5B_HUMAN | RAB5B |
|  | 8 | 40S ribosomal protein S6 | RS6_HUMAN | RPS6 |

# Appendix E: Conventional statistical approach

The conventional statistical approach, grounded in the principles of the Wilcoxon signed-rank test or the t-test based on data distribution, adjusted with Bonferroni correction for multiple comparisons, serves to validate the machine learning findings by quantifying the univariate significance of individual protein expression differences. This one-dimensional view is essential for identifying potential biomarkers, categorized with asterisks in Table 2 to denote varying levels of significance. We refined our basic analysis by implementing the Kruskal–Wallis non-parametric ANOVA, complemented with Dunn's post-hoc test and adjusted using the Bonferroni correction. This enhanced method was designed to evaluate the differences in protein expression across three specific conditions in a cohesive manner, moving beyond isolated pairwise comparisons. Through the Kruskal–Wallis test, we identified nine proteins (BCAT2, RSMB, 14-3-3F, ABHDA, EH1L1, RL30, MK01, MIC13, TBB6) exhibiting p-values below 0.05. Subsequent analysis with Dunn's post-hoc tests highlighted the statistically significant disparities in expression levels among these proteins across the conditions. Comparing these findings with the data presented in following Table, we observed a significant overlap in the identified features, reinforcing the robustness of our analytical approach.

| **Problem** | **Significant proteins** | | | | | | | |
| --- | --- | --- | --- | --- | --- | --- | --- | --- |
| **PD-NG** | BCAT2** | RSMB** | ABHDA** | 1433F* | ALDR* |  |  |  |
| **T2D-PD** | LANC1* |  |  |  |  |  |  |  |
| **T2D-NG** | PROB1*** | TMM11** | CHM4B** | PANK4** | EH1L1** | RL37A** | SPEG** | VPS28** |
|  | GET3** | FN3K** | PHS** | UB2L3** | RAS4B** | RAB5B** | ACS2L** | RL10** |
|  | PMGE* | TPP2* | EIF3M* | IMPA1* | XPO1* | HIG1A* | UBE2N* | TXNL1* |
|  | RS6* | SLMAP* | TADBP* | PRS7* | ANK3* | HNRC2* | MGST3* | STIM1* |
|  | PPR3A* | RRAS2* | RS26* | PRR33* | PSD11* | MCEE* | CSN1* | COX7C* |
|  | PA1B2* | DRS7B* |  |  |  |  |  |  |

***Table****. Differential expression of proteins across different glycemic states. The asterisks denote significance levels with *** indicating p < 0.001, ** for p < 0.01, and * for p < 0.05.*

The IntelliOmics platform ^119^ was used to perform statistical validation. The t-test is applied to compare the means of two independent samples, positing a null hypothesis that both groups have equivalent average values. The assumption of equal variances, which the t-test defaults to, can significantly affect the test's reliability and is therefore carefully assessed before the test application. On the other hand, the Wilcoxon rank-sum test offers a non-parametric alternative, making no assumptions about the distribution of the data. It evaluates whether two samples are likely to derive from the same distribution. The Wilcoxon test is particularly useful when dealing with skewed distributions or when the sample sizes are small.

To account for the multiple comparisons problem and to control the family-wise error rate, a Bonferroni correction was applied to the p-values obtained from the tests. This adjustment is stringent and ensures that the likelihood of identifying false positives (type I errors) is minimized when multiple pairwise tests are conducted.

The statistical analysis focused on pinpointing the most significant proteins for each classification problem. The selection was based on the adjusted p-values, with the level of significance denoted by asterisks in Table 2: *** indicates a highly significant difference with a p-value < 0.001; ** denotes a p-value < 0.01; * signifies a p-value < 0.05.

This traditional method highlighted only a single significant hit between T2D and PD and five between NG and PD. Such results may reflect the conservative nature of the Bonferroni correction, which is well-suited for controlling the false discovery rate but at the risk of being overly stringent, potentially overlooking meaningful biological variations. Here are the results:

**NG vs PD**

['bcat2_human', 'rsmb_human__rsmn_human', 'eh1l1_human', 'abhda_human', '1433f_human', 'aldr_human']

[0.00130396, 0.00540107, 0.00764402, 0.00793747, 0.0193103, 0.038934]

**NG vs T2D**

['prob1_human', 'tmm11_human', 'chm4b_human', 'pank4_human', 'eh1l1_human', 'rl37a_human', 'speg_human', 'vps28_human', 'get3_human', 'fn3k_human', 'phs_human', 'ub2l3_human', 'csn1_human', 'cox7c_human', 'pa1b2_human', 'ras4b_human__rasl2_human', 'rab5b_human', 'acs2l_human', 'drs7b_human', 'rl10_human', 'rsmb_human__rsmn_human', 'pmge_human', 'tpp2_human', 'eif3m_human', 'bcat2_human', 'impa1_human', 'xpo1_human', 'hig1a_human', 'ube2n_human', 'txnl1_human', 'rs6_human', 'slmap_human', 'tadbp_human', 'prs7_human', 'ank3_human', 'hnrc2_human__hnrc3_human__hnrc1_human__hnrpc_human__hnrc4_human', 'mgst3_human', 'stim1_human', 'ppr3a_human', 'rras2_human', 'rs26_human', 'prr33_human', 'psd11_human', 'mcee_human']

[0.000593899, 0.00103214, 0.00136301, 0.00145789, 0.00155439, 0.00177149, 0.00188546, 0.0020176, 0.00261208, 0.00296921, 0.00296921, 0.00492548, 0.00603003, 0.0068121, 0.00715085, 0.00715085, 0.00798648, 0.00808663, 0.00913895, 0.00913895, 0.00913895, 0.0103216, 0.0116497, 0.0119807, 0.0131259, 0.0148119, 0.0148119, 0.0168581, 0.0175915, 0.0190706, 0.0211331, 0.0211331, 0.0211331, 0.0228616, 0.0237605, 0.0266973, 0.0266973, 0.0266973, 0.0309778, 0.0377251, 0.0377251, 0.0393615, 0.0406838, 0.0473527]

**PD vs T2D**

['lanc1_human']

[0.0372838]

We extended our analysis by also performing the Kruskal–Wallis non-parametric ANOVA with Dunn's post-correction (adjusted with Bonferroni correction). This additional analysis aimed to address the evaluation of differing proteins among the three conditions in a more integrated manner, as opposed to conducting individual comparisons. The Kruskal–Wallis test identified nine proteins ('bcat2’, 'rsmb_rsmn’, '1433f, 'abhda', 'eh1l1', 'rl30', 'mk01’, 'mic13’, 'tbb6' ) with p-values below 0.05, and subsequent Dunn's post-hoc tests further elucidated the statistical significance of differences between conditions for these proteins.

**Kruskal**

['bcat2_human', 'rsmb_human__rsmn_human', '1433f_human', 'abhda_human', 'eh1l1_human', 'rl30_human', 'mk01_human', 'mic13_human', 'tbb6_human']

[8.54532e-05, 0.00267321, 0.00302746, 0.00438203, 0.00903516, 0.0114455, 0.0162465, 0.0204593, 0.0402833]

**Dunn’s post-hoc tests for the Kruskal findings:**

**bcat2_human**

NG PD T2D

NG 1.000000 0.000002 0.000241

PD 0.000002 1.000000 1.000000

T2D 0.000241 1.000000 1.000000

**rsmb_human__rsmn_human**

NG PD T2D

NG 1.000000 0.000079 0.000006

PD 0.000079 1.000000 1.000000

T2D 0.000006 1.000000 1.000000

**1433f_human**

NG PD T2D

NG 1.000000 0.000045 0.000249

PD 0.000045 1.000000 1.000000

T2D 0.000249 1.000000 1.000000

**abhda_human**

NG PD T2D

NG 1.000000 0.000049 0.000345

PD 0.000049 1.000000 1.000000

T2D 0.000345 1.000000 1.000000

**eh1l1_human**

NG PD T2D

NG 1.000000e+00 0.000256 6.268376e-07

PD 2.564206e-04 1.000000 3.586765e-01

T2D 6.268376e-07 0.358676 1.000000e+00

**rl30_human**

NG PD T2D

NG 1.000000 0.000145 0.000094

PD 0.000145 1.000000 1.000000

T2D 0.000094 1.000000 1.000000

**mk01_human**

NG PD T2D

NG 1.000000 0.000094 0.004051

PD 0.000094 1.000000 1.000000

T2D 0.004051 1.000000 1.000000

**mic13_human**

NG PD T2D

NG 1.000000 0.001657 0.000022

PD 0.001657 1.000000 0.588183

T2D 0.000022 0.588183 1.000000

**tbb6_human**

NG PD T2D

NG 1.000000 0.000699 0.000071

PD 0.000699 1.000000 1.000000

T2D 0.000071 1.000000 1.000000

# Appendix F: Protein details from statistics tailored for proteome data analysis in Figure 4

| **Comparison** | **Nr** | **Protein description** | **UniProtKB identifier** | **Gene name** | **P-value (-Log10)** | **Fold change (Log2)** |
| --- | --- | --- | --- | --- | --- | --- |
| **T2D-NG** | 1 | Tripeptidyl-peptidase 2 | TPP2_HUMAN | TPP2 | 19.2586168 | -0.960835959 |
|  | 2 | COP9 signalosome complex subunit 1 | CSN1_HUMAN | GPS1 | 19.22517583 | -1.424666345 |
|  | 3 | EH domain-binding protein 1-like protein 1 | EH1L1_HUMAN | EHBP1L1 | 19.03102894 | -0.953124805 |
|  | 4 | Inositol monophosphatase 1 | IMPA1_HUMAN | IMPA1 | 18.70510909 | -1.523157482 |
|  | 5 | ATPase GET3 | GET3_HUMAN | GET3 | 18.62478379 | -1.393925988 |
|  | 6 | Palmitoyl-protein thioesterase ABHD10, mitochondrial | ABHDA_HUMAN | ABHD10 | 18.29771775 | -0.788447684 |
|  | 7 | Ubiquitin-conjugating enzyme E2 L3 | UB2L3_HUMAN | UBE2L3 | 17.69029388 | -1.097520104 |
|  | 8 | Small nuclear ribonucleoprotein-associated proteins B and B' | RSMB_HUMAN | SNRPB | 17.64725489 | -1.163587044 |
|  | 9 | Small nuclear ribonucleoprotein-associated protein N | RSMN_HUMAN | SNRPN | 17.64725489 | -1.163587044 |
|  | 10 | Acetyl-coenzyme A synthetase 2-like, mitochondrial | ACS2L_HUMAN | ACSS1 | 17.64725489 | -1.214285633 |
|  | 11 | 60S ribosomal protein L37a | RL37A_HUMAN | RPL37A | 17.58708657 | -1.277950481 |
|  | 12 | Palladin | PALLD_HUMAN | PALLD | 17.43354153 | -1.080176724 |
|  | 13 | Endothelial differentiation-related factor 1 | EDF1_HUMAN | EDF1 | 17.38797605 | -0.739317037 |
|  | 14 | Ras-related protein Rab-5B | RAB5B_HUMAN | RAB5B | 16.97746914 | -0.894584571 |
|  | 15 | TAR DNA-binding protein 43 | TADBP_HUMAN | TARDBP | 16.93566913 | -1.003079136 |
|  | 16 | Cytochrome c oxidase subunit 7C, mitochondrial | COX7C_HUMAN | COX7C | 16.69161816 | -0.762099169 |
|  | 17 | Triadin | TRDN_HUMAN | TRDN | 16.69161816 | -1.630607801 |
|  | 18 | Fructosamine-3-kinase | FN3K_HUMAN | FN3K | 16.69161816 | -0.677481371 |
|  | 19 | Vacuolar protein sorting-associated protein 28 homolog | VPS28_HUMAN | VPS28 | 16.51739883 | -1.101185895 |
|  | 20 | Equilibrative nucleoside transporter 1 | S29A1_HUMAN | SLC29A1 | 16.36875873 | -1.177893483 |
|  | 21 | 3-ketoacyl-CoA thiolase, peroxisomal | THIK_HUMAN | ACAA1 | 16.28367554 | -0.705535427 |
|  | 22 | HIG1 domain family member 1A, mitochondrial | HIG1A_HUMAN | HIGD1A | 16.18122087 | -0.857243452 |
|  | 23 | 4'-phosphopantetheine phosphatase | PANK4_HUMAN | PANK4 | 16.11483893 | -0.653885261 |
|  | 24 | Stromal interaction molecule 1 | STIM1_HUMAN | STIM1 | 16.0362008 | -1.246878757 |
|  | 25 | DNA-(apurinic or apyrimidinic site) endonuclease | APEX1_HUMAN | APEX1 | 15.98066059 | -1.063664316 |
|  | 26 | 40S ribosomal protein S26 | RS26_HUMAN | RPS26 | 15.66982109 | -0.940566388 |
|  | 27 | Transmembrane protein 11, mitochondrial | TMM11_HUMAN | TMEM11 | 15.56324563 | -0.612790556 |
|  | 28 | Crk-like protein | CRKL_HUMAN | CRKL | 15.56324563 | -1.31604401 |
|  | 29 | Dehydrogenase/reductase SDR family member 7B | DRS7B_HUMAN | DHRS7B | 15.56324563 | -0.836084894 |
|  | 30 | E3 ubiquitin-protein ligase RNF170 | RN170_HUMAN | RNF170 | 15.50276471 | -1.133136989 |
|  | 31 | Pterin-4-alpha-carbinolamine dehydratase | PHS_HUMAN | PCBD1 | 14.89186623 | -1.840396697 |
|  | 32 | 2'-deoxynucleoside 5'-phosphate N-hydrolase 1 | DNPH1_HUMAN | DNPH1 | 14.84619115 | -0.749556365 |
|  | 33 | 60S ribosomal protein L28 | RL28_HUMAN | RPL28 | 14.84619115 | -1.08843952 |
|  | 34 | Glutathione reductase, mitochondrial | GSHR_HUMAN | GSR | 14.80462182 | -0.795338143 |
|  | 35 | Methylmalonyl-CoA epimerase, mitochondrial | MCEE_HUMAN | MCEE | 14.80462182 | -1.253817287 |
|  | 36 | 5'-AMP-activated protein kinase subunit beta-2 | AAKB2_HUMAN | PRKAB2 | 14.77871595 | -0.675550814 |
|  | 37 | Complement C4-B | CO4B_HUMAN | C4B | 14.77871595 | -1.380211931 |
|  | 38 | Lon protease homolog, mitochondrial | LONM_HUMAN | LONP1 | 14.77095968 | -0.603272436 |
|  | 39 | Proline-rich protein 33 | PRR33_HUMAN | PRR33 | 14.39786592 | -0.9325654 |
|  | 40 | Thioredoxin-like protein 1 | TXNL1_HUMAN | TXNL1 | 14.37875816 | -0.590619722 |
|  | 41 | Plasminogen activator inhibitor 1 RNA-binding protein | PAIRB_HUMAN | SERBP1 | 14.37875816 | -0.945792608 |
|  | 42 | Thioredoxin-related transmembrane protein 4 | TMX4_HUMAN | TMX4 | 14.29316509 | -0.888819938 |
| **Comparison** | **Nr** | **Protein description** | **UniProtKB identifier** | **Gene name** | **P-value (-Log10)** | **Fold change (Log2)** |
| **T2D-NG** | 43 | Gamma-glutamylcyclotransferase | GGCT_HUMAN | GGCT | 14.26914603 | -1.083610233 |
|  | 44 | Protein preY, mitochondrial | PREY_HUMAN | PYURF | 14.12485063 | -0.783197409 |
|  | 45 | Alpha-mannosidase 2C1 | MA2C1_HUMAN | MAN2C1 | 13.86120831 | -0.679302656 |
|  | 46 | Carnosine synthase 1 | CRNS1_HUMAN | CARNS1 | 13.71135298 | -0.891231292 |
|  | 47 | NADH-ubiquinone oxidoreductase chain 2 | NU2M_HUMAN | MT-ND2 | 13.71135298 | -1.275725853 |
|  | 48 | 40S ribosomal protein S13 | RS13_HUMAN | RPS13 | 13.69348897 | -0.727999057 |
|  | 49 | Monocarboxylate transporter 1 | MOT1_HUMAN | SLC16A1 | 13.68697364 | -0.760349937 |
|  | 50 | Ras-related protein R-Ras2 | RRAS2_HUMAN | RRAS2 | 13.66375712 | -1.473026972 |
|  | 51 | Transmembrane protein 126A | T126A_HUMAN | TMEM126A | 13.59184972 | -0.839477951 |
|  | 52 | Mitochondrial coenzyme A transporter SLC25A42 | S2542_HUMAN | SLC25A42 | 13.55022806 | -1.604800895 |
|  | 53 | GDH/6PGL endoplasmic bifunctional protein | G6PE_HUMAN | H6PD | 13.50928296 | -0.832360405 |
|  | 54 | NIF3-like protein 1 | NIF3L_HUMAN | NIF3L1 | 13.38510065 | -1.144693678 |
|  | 55 | Hsp90 co-chaperone Cdc37 | CDC37_HUMAN | CDC37 | 13.23134772 | -0.731700799 |
|  | 56 | Diablo homolog, mitochondrial | DBLOH_HUMAN | DIABLO | 13.21946929 | -0.667286317 |
|  | 57 | SUN domain-containing protein 2 | SUN2_HUMAN | SUN2 | 13.21725799 | -1.004489853 |
|  | 58 | Protein phosphatase 1 regulatory subunit 7 | PP1R7_HUMAN | PPP1R7 | 13.11180557 | -1.495216811 |
|  | 59 | Eukaryotic translation initiation factor 3 subunit M | EIF3M_HUMAN | EIF3M | 12.99865078 | -0.769623986 |
|  | 60 | 60S ribosomal protein L27 | RL27_HUMAN | RPL27 | 12.99482915 | -1.048560296 |
|  | 61 | Copper homeostasis protein cutC homolog | CUTC_HUMAN | CUTC | 12.99482915 | -0.822006307 |
|  | 62 | Atlastin-2 | ATLA2_HUMAN | ATL2 | 12.78456688 | -1.414855807 |
|  | 63 | 40S ribosomal protein S6 | RS6_HUMAN | RPS6 | 12.7029841 | -1.291094916 |
|  | 64 | Dual specificity phosphatase 29 | DUS29_HUMAN | DUSP29 | 12.6981119 | -1.956593834 |
|  | 65 | Pyroglutamyl-peptidase 1 | PGPI_HUMAN | PGPEP1 | 12.65355868 | -0.974052182 |
|  | 66 | Proline-rich basic protein 1 | PROB1_HUMAN | PROB1 | 12.64577013 | -0.788297524 |
|  | 67 | Phospholipase A-2-activating protein | PLAP_HUMAN | PLAA | 12.59799701 | -0.645619406 |
|  | 68 | Exportin-1 | XPO1_HUMAN | XPO1 | 12.58154704 | -0.779615542 |
|  | 69 | 6-phosphogluconolactonase | 6PGL_HUMAN | PGLS | 12.55259566 | -0.829884681 |
|  | 70 | Platelet-activating factor acetylhydrolase IB subunit alpha2 | PA1B2_HUMAN | PAFAH1B2 | 12.51593184 | -1.098059225 |
|  | 71 | Cdc42-interacting protein 4 | CIP4_HUMAN | TRIP10 | 12.50617807 | -0.616441888 |
|  | 72 | Glucose-6-phosphate exchanger SLC37A4 | G6PT1_HUMAN | SLC37A4 | 12.4789557 | -0.829255056 |
|  | 73 | Copine-3 | CPNE3_HUMAN | CPNE3 | 12.44308303 | -0.79022084 |
|  | 74 | Protein ABHD14B | ABHEB_HUMAN | ABHD14B | 12.44308303 | -0.705446443 |
|  | 75 | Sarcolemmal membrane-associated protein | SLMAP_HUMAN | SLMAP | 12.37571557 | -1.184183147 |
|  | 76 | ATP-dependent (S)-NAD(P)H-hydrate dehydratase | NNRD_HUMAN | NAXD | 12.33312723 | -0.830381724 |
|  | 77 | GTP-binding protein SAR1a | SAR1A_HUMAN | SAR1A | 12.2720601 | -0.628243267 |
|  | 78 | Ankyrin-3 | ANK3_HUMAN | ANK3 | 12.18183096 | -1.675415176 |
|  | 79 | Branched-chain-amino-acid aminotransferase, mitochondrial | BCAT2_HUMAN | BCAT2 | 12.14032855 | -0.797862155 |
|  | 80 | Eukaryotic translation initiation factor 3 subunit J | EIF3J_HUMAN | EIF3J | 11.96247537 | -0.727500478 |
|  | 81 | Metaxin-2 | MTX2_HUMAN | MTX2 | 11.93698852 | -0.61633402 |
|  | 82 | AP-2 complex subunit beta | AP2B1_HUMAN | AP2B1 | 11.90748284 | -0.849879904 |
|  | 83 | Tubulin beta-6 chain | TBB6_HUMAN | TUBB6 | 11.90748284 | -1.151921726 |
|  | 84 | Caveolin-1 | CAV1_HUMAN | CAV1 | 11.86963459 | -0.929824035 |
|  | 85 | Bifunctional coenzyme A synthase | COASY_HUMAN | COASY | 11.86963459 | -0.882058791 |
|  | 86 | 60S ribosomal protein L10 | RL10_HUMAN | RPL10 | 11.79884213 | -0.608931046 |
|  | 87 | Smoothelin-like protein 2 | SMTL2_HUMAN | SMTNL2 | 11.72673161 | -0.619224986 |
| **Comparison** | **Nr** | **Protein description** | **UniProtKB identifier** | **Gene name** | **P-value (-Log10)** | **Fold change (Log2)** |
| **T2D-NG** | 88 | Pyridoxal kinase | PDXK_HUMAN | PDXK | 11.50132694 | -0.788552585 |
|  | 89 | 5'-deoxynucleotidase HDDC2 | HDDC2_HUMAN | HDDC2 | 11.4831995 | -0.816702735 |
|  | 90 | 60S ribosomal protein L13 | RL13_HUMAN | RPL13 | 11.37606935 | -0.693297468 |
|  | 91 | Proteasome activator complex subunit 2 | PSME2_HUMAN | PSME2 | 11.27204068 | -0.740131199 |
|  | 92 | 6-phosphogluconate dehydrogenase, decarboxylating | 6PGD_HUMAN | PGD | 11.25714635 | -0.701180046 |
|  | 93 | Complex III assembly factor LYRM7 | LYRM7_HUMAN | LYRM7 | 11.23613516 | -1.025692015 |
|  | 94 | Translocator protein | TSPO_HUMAN | TSPO | 11.18397681 | -0.67791508 |
|  | 95 | Dual specificity mitogen-activated protein kinase kinase 3 | MP2K3_HUMAN | MAP2K3 | 11.09110251 | -0.663197136 |
|  | 96 | Porphobilinogen deaminase | HEM3_HUMAN | HMBS | 11.08594913 | -0.776192424 |
|  | 97 | 26S proteasome regulatory subunit 7 | PRS7_HUMAN | PSMC2 | 11.01484719 | -0.875377358 |
|  | 98 | Glycine cleavage system H protein, mitochondrial | GCSH_HUMAN | GCSH | 11.00005746 | -0.98772039 |
|  | 99 | Interferon-inducible double-stranded RNA-dependent protein kinase activator A | PRKRA_HUMAN | PRKRA | 10.95242708 | -0.894359277 |
|  | 100 | Protein arginine N-methyltransferase 1 | ANM1_HUMAN | PRMT1 | 10.92928677 | -0.967961862 |
|  | 101 | Cardiac phospholamban | PPLA_HUMAN | PLN | 10.89873474 | -0.797985 |
|  | 102 | Calcium/calmodulin-dependent protein kinase type II subunit delta | KCC2D_HUMAN | CAMK2D | 10.89873474 | -0.652884654 |
|  | 103 | Eukaryotic translation initiation factor 3 subunit A | EIF3A_HUMAN | EIF3A | 10.89704153 | -0.644882308 |
|  | 104 | Biliverdin reductase A | BIEA_HUMAN | BLVRA | 10.79152866 | -0.955079005 |
|  | 105 | Moesin | MOES_HUMAN | MSN | 10.76632119 | -1.090704746 |
|  | 106 | Mitogen-activated protein kinase 1 | MK01_HUMAN | MAPK1 | 10.7514891 | -1.093732029 |
|  | 107 | 40S ribosomal protein S21 | RS21_HUMAN | RPS21 | 10.67234189 | -0.772441843 |
|  | 108 | Caveolae-associated protein 4 | CAVN4_HUMAN | CAVIN4 | 10.61441718 | -1.301013643 |
|  | 109 | Serine/threonine-protein kinase Nek7 | NEK7_HUMAN | NEK7 | 10.61290582 | -1.081864276 |
|  | 110 | Heterogeneous nuclear ribonucleoprotein A3 | ROA3_HUMAN | HNRNPA3 | 10.47430046 | -0.986132307 |
|  | 111 | T-complex protein 1 subunit alpha | TCPA_HUMAN | TCP1 | 10.37751831 | -0.647590902 |
|  | 112 | Bisphosphoglycerate mutase | PMGE_HUMAN | BPGM | 10.35990461 | -1.047747983 |
|  | 113 | Short/branched chain specific acyl-CoA dehydrogenase, mitochondrial | ACDSB_HUMAN | ACADSB | 10.17865873 | -0.632994083 |
|  | 114 | NEDD8 | NEDD8_HUMAN | NEDD8 | 10.09155594 | -0.964274802 |
|  | 115 | Ras GTPase-activating protein 4B | RAS4B_HUMAN | RASA4B | 9.993111309 | -2.266477549 |
|  | 116 | Ras GTPase-activating protein 4 | RASL2_HUMAN | RASA4 | 9.993111309 | -2.266477549 |
|  | 117 | Isobutyryl-CoA dehydrogenase, mitochondrial | ACAD8_HUMAN | ACAD8 | 9.993111309 | -0.699769253 |
|  | 118 | PGC-1 and ERR-induced regulator in muscle protein 1 | PERM1_HUMAN | PERM1 | 9.990319012 | -0.654952248 |
|  | 119 | Heterogeneous nuclear ribonucleoprotein D0 | HNRPD_HUMAN | HNRNPD | 9.941543329 | -1.187424382 |
|  | 120 | Coiled-coil domain-containing protein 72 | A0A024R1R8_HUMAN | TMA7B | 9.840656756 | -1.105470271 |
|  | 121 | Translation machinery-associated protein 7 | TMA7_HUMAN | TMA7 | 9.840656756 | -1.105470271 |
|  | 122 | Membrane-associated progesterone receptor component 2 | PGRC2_HUMAN | PGRMC2 | 9.82069182 | -0.846529722 |
|  | 123 | Charged multivesicular body protein 4b | CHM4B_HUMAN | CHMP4B | 9.771127409 | -1.669911639 |
|  | 124 | SH3 and cysteine-rich domain-containing protein 3 | STAC3_HUMAN | STAC3 | 9.752777576 | -1.01665269 |
|  | 125 | Enoyl-CoA hydratase domain-containing protein 3, mitochondrial | ECHD3_HUMAN | ECHDC3 | 9.679057352 | -0.717696361 |
|  | 126 | GMP reductase 1 | GMPR1_HUMAN | GMPR | 9.550121966 | -0.624984857 |
|  | 127 | Serine-threonine kinase receptor-associated protein | STRAP_HUMAN | STRAP | 9.491837439 | -0.65120016 |
|  | 128 | Ras-related protein Rab-21 | RAB21_HUMAN | RAB21 | 9.370258258 | -0.593764863 |
|  | 129 | Transcription factor A, mitochondrial | TFAM_HUMAN | TFAM | 9.35710295 | -0.737489061 |
|  | 130 | Very-long-chain 3-oxoacyl-CoA reductase | DHB12_HUMAN | HSD17B12 | 9.343756448 | -0.715377262 |
|  | 131 | ATP-dependent RNA helicase DDX19A | DD19A_HUMAN | DDX19A | 9.15026453 | -1.190137999 |
|  | 132 | Apolipoprotein E | APOE_HUMAN | APOE | 9.090665384 | -0.710119078 |
| **Comparison** | **Nr** | **Protein description** | **UniProtKB identifier** | **Gene name** | **P-value (-Log10)** | **Fold change (Log2)** |
| **T2D-NG** | 133 | Cytosolic non-specific dipeptidase | CNDP2_HUMAN | CNDP2 | 9.077715724 | -0.730457486 |
|  | 134 | Protein S100-A4 | S10A4_HUMAN | S100A4 | 9.020578933 | -1.122617243 |
|  | 135 | Proteasome inhibitor PI31 subunit | PSMF1_HUMAN | PSMF1 | 8.999640733 | -0.651593528 |
|  | 136 | MICOS complex subunit MIC13 | MIC13_HUMAN | MICOS13 | 8.9015951 | -0.606412357 |
|  | 137 | 26S proteasome non-ATPase regulatory subunit 11 | PSD11_HUMAN | PSMD11 | 8.858925902 | -0.800974738 |
|  | 138 | Prostaglandin E synthase 3 | TEBP_HUMAN | PTGES3 | 8.81325385 | -1.286301627 |
|  | 139 | Heterogeneous nuclear ribonucleoprotein C-like 2 | HNRC2_HUMAN | HNRNPCL2 | 8.706531097 | -1.733076965 |
|  | 140 | Heterogeneous nuclear ribonucleoprotein C-like 3 | HNRC3_HUMAN | HNRNPCL3 | 8.706531097 | -1.733076965 |
|  | 141 | Heterogeneous nuclear ribonucleoprotein C-like 1 | HNRC1_HUMAN | HNRNPCL1 | 8.706531097 | -1.733076965 |
|  | 142 | Heterogeneous nuclear ribonucleoproteins C1/C2 | HNRPC_HUMAN | HNRNPC | 8.706531097 | -1.733076965 |
|  | 143 | Heterogeneous nuclear ribonucleoprotein C-like 4 | HNRC4_HUMAN | HNRNPC4 | 8.706531097 | -1.733076965 |
|  | 144 | Histone H1.0 | H10_HUMAN | H1-0 | 8.66131599 | -0.648685578 |
|  | 145 | Inter-alpha-trypsin inhibitor heavy chain H4 | ITIH4_HUMAN | ITIH4 | 8.601354626 | -0.670509479 |
|  | 146 | 40S ribosomal protein S12 | RS12_HUMAN | RPS12 | 8.593753421 | -0.845842873 |
|  | 147 | AMP deaminase 1 | AMPD1_HUMAN | AMPD1 | 8.461200526 | 0.628052268 |
|  | 148 | ATPase family AAA domain-containing protein 3A | ATD3A_HUMAN | ATAD3A | 8.455824426 | -0.817588215 |
|  | 149 | Methylcrotonoyl-CoA carboxylase beta chain, mitochondrial | MCCB_HUMAN | MCCC2 | 8.368325382 | -0.621076969 |
|  | 150 | Propionyl-CoA carboxylase alpha chain, mitochondrial | PCCA_HUMAN | PCCA | 8.247319744 | -0.760508651 |
|  | 151 | Ferrochelatase, mitochondrial | HEMH_HUMAN | FECH | 8.197090941 | -0.627715578 |
|  | 152 | Mitogen-activated protein kinase 12 | MK12_HUMAN | MAPK12 | 8.186683792 | -0.625970626 |
|  | 153 | 60S ribosomal protein L5 | RL5_HUMAN | RPL5 | 8.181085134 | -0.67036951 |
|  | 154 | Beta-1-syntrophin | SNTB1_HUMAN | SNTB1 | 7.978421138 | -0.826789672 |
|  | 155 | Hypoxanthine-guanine phosphoribosyltransferase | HPRT_HUMAN | HPRT1 | 7.950272123 | -0.836196284 |
|  | 156 | Leiomodin-2 | LMOD2_HUMAN | LMOD2 | 7.761822227 | -0.680936038 |
|  | 157 | Endoplasmin | ENPL_HUMAN | HSP90B1 | 7.733747477 | -1.092729185 |
|  | 158 | Immunoglobulin heavy constant gamma 3 | IGHG3_HUMAN | IGHG3 | 7.712476895 | -1.080352986 |
|  | 159 | Myosin-binding protein H | MYBPH_HUMAN | MYBPH | 7.60537302 | -2.196416429 |
|  | 160 | Tripeptidyl-peptidase 1 | TPP1_HUMAN | TPP1 | 7.493891396 | -0.905758593 |
|  | 161 | Basigin | BASI_HUMAN | BSG | 7.448666066 | -0.674941666 |
|  | 162 | Nucleophosmin | NPM_HUMAN | NPM1 | 7.347246131 | -1.087475935 |
|  | 163 | Signal recognition particle 14 kDa protein | SRP14_HUMAN | SRP14 | 7.308053295 | -0.812674525 |
|  | 164 | Protein 4.1 | EPB41_HUMAN | EPB41 | 7.102440648 | -1.301451253 |
|  | 165 | Muscular LMNA-interacting protein | MLIP_HUMAN | MLIP | 6.990818598 | -0.736118139 |
|  | 166 | 40S ribosomal protein S20 | RS20_HUMAN | RPS20 | 6.952153852 | -0.649300459 |
|  | 167 | Eukaryotic translation initiation factor 1A, Y-chromosomal | IF1AY_HUMAN | EIF1AY | 6.838558411 | -0.707178762 |
|  | 168 | Eukaryotic translation initiation factor 1A, X-chromosomal | IF1AX_HUMAN | EIF1AX | 6.838558411 | -0.707178762 |
|  | 169 | 60S ribosomal protein L17 | RL17_HUMAN | RPL17 | 6.790067444 | -0.618311633 |
|  | 170 | V-type proton ATPase subunit B, brain isoform | VATB2_HUMAN | ATP6V1B2 | 6.70359078 | -1.225687611 |
|  | 171 | Endoplasmic reticulum aminopeptidase 1 | ERAP1_HUMAN | ERAP1 | 6.63449319 | -0.838184331 |
|  | 172 | Programmed cell death protein 5 | PDCD5_HUMAN | PDCD5 | 6.548411554 | -0.880527295 |
|  | 173 | 14-3-3 protein eta | 1433F_HUMAN | YWHAH | 6.344258617 | -0.975157108 |
|  | 174 | Alpha-actinin-3 | ACTN3_HUMAN | ACTN3 | 6.336388514 | 1.264340383 |
|  | 175 | ATP synthase protein 8 | ATP8_HUMAN | MT-ATP8 | 6.289301061 | -0.646860786 |
|  | 176 | Aldehyde dehydrogenase family 3 member A2 | AL3A2_HUMAN | ALDH3A2 | 6.185764673 | -0.763288628 |
|  | 177 | 28 kDa heat- and acid-stable phosphoprotein | HAP28_HUMAN | PDAP1 | 6.177762302 | -0.619205331 |

| **Comparison** | **Nr** | **Protein description** | **UniProtKB identifier** | **Gene name** | **P-value (-Log10)** | **Fold change (Log2)** |
| --- | --- | --- | --- | --- | --- | --- |
| **T2D-PD** | 178 | Methyltransferase-like protein 7A | MET7A_HUMAN | METTL7A | 6.168002212 | 0.614144522 |
|  | 179 | Heterogeneous nuclear ribonucleoprotein U | HNRPU_HUMAN | HNRNPU | 6.155272519 | -0.610229843 |
|  | 180 | Translational activator of cytochrome c oxidase 1 | TACO1_HUMAN | TACO1 | 5.982369379 | -0.635469048 |
|  | 181 | Tubulin beta-2A chain | TBB2A_HUMAN | TUBB2A | 5.893698542 | -0.708925403 |
|  | 182 | Tubulin beta-2B chain | TBB2B_HUMAN | TUBB2B | 5.893698542 | -0.708925403 |
|  | 183 | Proteasome subunit alpha type-4 | PSA4_HUMAN | PSMA4 | 5.880600498 | 0.849700541 |
|  | 184 | Isopentenyl-diphosphate Delta-isomerase 1 | IDI1_HUMAN | IDI1 | 5.838538742 | -1.243696484 |
|  | 185 | 60S ribosomal protein L21 | RL21_HUMAN | RPL21 | 5.822244181 | -1.599401797 |
|  | 186 | Citramalyl-CoA lyase, mitochondrial | CLYBL_HUMAN | CLYBL | 5.817825829 | -1.12608342 |
|  | 187 | Alpha-synuclein | SYUA_HUMAN | SNCA | 5.169969438 | -0.980565549 |
|  | 188 | Complement factor B | CFAB_HUMAN | CFB | 5.070643344 | -0.614617412 |
|  | 189 | Protein 4.2 | EPB42_HUMAN | EPB42 | 5.039754952 | -0.79872249 |
|  | 190 | Alpha-hemoglobin-stabilizing protein | AHSP_HUMAN | AHSP | 5.012097604 | -0.978119632 |
|  | 191 | Ras-related protein Rab-2A | RAB2A_HUMAN | RAB2A | 4.987678275 | -0.658791126 |
|  | 192 | Inter-alpha-trypsin inhibitor heavy chain H1 | ITIH1_HUMAN | ITIH1 | 4.940417928 | -0.596076103 |
|  | 193 | Solute carrier family 2, facilitated glucose transporter member 1 | GTR1_HUMAN | SLC2A1 | 4.923233941 | -0.726410213 |
|  | 194 | All-trans-retinol dehydrogenase [NAD(+)] ADH1B | ADH1B_HUMAN | ADH1B | 4.820606847 | -0.85153097 |
|  | 195 | Catechol O-methyltransferase domain-containing protein 1 | CMTD1_HUMAN | COMTD1 | 4.297323646 | -0.852577943 |
|  | 196 | Ankyrin-1 | ANK1_HUMAN | ANK1 | 4.105368653 | -0.621269381 |
|  | 197 | Dermatopontin | DERM_HUMAN | DPT | 3.862423216 | -1.309915988 |
|  | 198 | Methylcrotonoyl-CoA carboxylase subunit alpha, mitochondrial | MCCA_HUMAN | MCCC1 | 3.647306899 | 0.632825119 |
|  | 199 | Mitochondrial import inner membrane translocase subunit TIM44 | TIM44_HUMAN | TIMM44 | 3.457214493 | -0.614808647 |
|  | 200 | Charged multivesicular body protein 2a | CHM2A_HUMAN | CHMP2A | 3.448868445 | -0.604499234 |
|  | 201 | Alpha-aminoadipic semialdehyde dehydrogenase | AL7A1_HUMAN | ALDH7A1 | 3.393868265 | -0.859557194 |
|  | 202 | Dihydropyrimidinase-related protein 2 | DPYL2_HUMAN | DPYSL2 | 3.345708469 | -3.608427536 |
|  | 203 | Smoothelin | SMTN_HUMAN | SMTN | 3.059816284 | -0.683961232 |
|  | 204 | Thioredoxin, mitochondrial | THIOM_HUMAN | TXN2 | 3.037604085 | -1.489449457 |
|  | 205 | Fibrinogen gamma chain | FIBG_HUMAN | FGG | 2.118787766 | -0.654903853 |
|  | 206 | Hemoglobin subunit theta-1 | HBAT_HUMAN | HBQ1 | 2.056967805 | -0.806488881 |
|  | 207 | Trifunctional purine biosynthetic protein adenosine-3 | PUR2_HUMAN | GART | 1.709455461 | -0.591723111 |

| **Comparison** | **Nr** | **Protein description** | **UniProtKB identifier** | **Gene name** | **P-value (-Log10)** | **Fold change (Log2)** |
| --- | --- | --- | --- | --- | --- | --- |
| **T2D-PD** | 1 | Ras GTPase-activating protein 4B | RAS4B_HUMAN | RASA4B | 16.29689172 | -0.987122164 |
|  | 2 | Ras GTPase-activating protein 4 | RASL2_HUMAN | RASA4 | 16.29689172 | -0.987122164 |
|  | 3 | Palladin | PALLD_HUMAN | PALLD | 13.977845 | -0.71638643 |
|  | 4 | Acetyl-coenzyme A synthetase 2-like, mitochondrial | ACS2L_HUMAN | ACSS1 | 13.80611311 | -0.87785461 |
|  | 5 | Equilibrative nucleoside transporter 1 | S29A1_HUMAN | SLC29A1 | 11.92751669 | -0.85274064 |
|  | 6 | Inositol monophosphatase 1 | IMPA1_HUMAN | IMPA1 | 11.78574598 | -1.111595725 |
|  | 7 | Thioredoxin, mitochondrial | THIOM_HUMAN | TXN2 | 11.73453909 | -0.923694368 |
|  | 8 | 60S ribosomal protein L28 | RL28_HUMAN | RPL28 | 11.52004438 | -0.757258161 |
|  | 9 | 60S ribosomal protein L21 | RL21_HUMAN | RPL21 | 10.759585 | -0.805232709 |
|  | 10 | Heterogeneous nuclear ribonucleoprotein C-like 2 | HNRC2_HUMAN | HNRNPCL2 | 10.67234189 | -0.730382192 |
|  | 11 | Heterogeneous nuclear ribonucleoprotein C-like 3 | HNRC3_HUMAN | HNRNPCL3 | 10.67234189 | -0.730382192 |
|  | 12 | Heterogeneous nuclear ribonucleoprotein C-like 1 | HNRC1_HUMAN | HNRNPCL1 | 10.67234189 | -0.730382192 |
|  | 13 | Heterogeneous nuclear ribonucleoproteins C1/C2 | HNRPC_HUMAN | HNRNPC | 10.67234189 | -0.730382192 |
|  | 14 | Heterogeneous nuclear ribonucleoprotein C-like 4 | HNRC4_HUMAN | HNRNPC4 | 10.67234189 | -0.730382192 |
|  | 15 | Triadin | TRDN_HUMAN | TRDN | 10.58623291 | -0.79321784 |
|  | 16 | Dihydropyrimidinase-related protein 2 | DPYL2_HUMAN | DPYSL2 | 10.55299055 | -1.321946408 |
|  | 17 | 3-ketoacyl-CoA thiolase, peroxisomal | THIK_HUMAN | ACAA1 | 10.53065959 | -0.586519587 |
|  | 18 | COP9 signalosome complex subunit 1 | CSN1_HUMAN | GPS1 | 10.37751831 | -0.885147739 |
|  | 19 | Complement C4-B | CO4B_HUMAN | C4B | 10.17865873 | -0.809326958 |
|  | 20 | Crk-like protein | CRKL_HUMAN | CRKL | 9.667703065 | -0.894180317 |
|  | 21 | Ankyrin-3 | ANK3_HUMAN | ANK3 | 9.635039511 | -0.753930604 |
|  | 22 | Porphobilinogen deaminase | HEM3_HUMAN | HMBS | 9.499900728 | -0.635711722 |
|  | 23 | 40S ribosomal protein S6 | RS6_HUMAN | RPS6 | 9.290310332 | -0.847154598 |
|  | 24 | Bisphosphoglycerate mutase | PMGE_HUMAN | BPGM | 9.077715724 | -0.721776985 |
|  | 25 | Biliverdin reductase A | BIEA_HUMAN | BLVRA | 8.843423097 | -0.750134485 |
|  | 26 | Dual specificity phosphatase 29 | DUS29_HUMAN | DUSP29 | 8.716869691 | -0.978690931 |
|  | 27 | 6-phosphogluconate dehydrogenase, decarboxylating | 6PGD_HUMAN | PGD | 8.565046689 | -0.655956491 |
|  | 28 | Charged multivesicular body protein 4b | CHM4B_HUMAN | CHMP4B | 8.281005885 | -1.234665395 |
|  | 29 | Immunoglobulin heavy variable 3-74 | HV374_HUMAN | IGHV3-74 | 8.23222306 | -0.71068808 |
|  | 30 | NIF3-like protein 1 | NIF3L_HUMAN | NIF3L1 | 8.213465756 | -0.630273869 |
|  | 31 | Stromal interaction molecule 1 | STIM1_HUMAN | STIM1 | 7.574111692 | -0.67031369 |
|  | 32 | Immunoglobulin heavy constant gamma 3 | IGHG3_HUMAN | IGHG3 | 7.556185183 | -0.943908046 |
|  | 33 | Methylmalonyl-CoA epimerase, mitochondrial | MCEE_HUMAN | MCEE | 7.421893538 | -0.706832907 |
|  | 34 | ATPase GET3 | GET3_HUMAN | GET3 | 7.266113682 | -0.796280164 |
|  | 35 | Apolipoprotein C-I | APOC1_HUMAN | APOC1 | 7.08982096 | -1.141216963 |
|  | 36 | Apolipoprotein B-100 | APOB_HUMAN | APOB | 7.08982096 | -0.665267164 |
|  | 37 | Inter-alpha-trypsin inhibitor heavy chain H4 | ITIH4_HUMAN | ITIH4 | 6.802338247 | -0.806655788 |
|  | 38 | Vacuolar protein sorting-associated protein 28 homolog | VPS28_HUMAN | VPS28 | 6.528811319 | -0.810346411 |
|  | 39 | Protein phosphatase 1 regulatory subunit 7 | PP1R7_HUMAN | PPP1R7 | 6.231388224 | -0.806363894 |
|  | 40 | Alpha-synuclein | SYUA_HUMAN | SNCA | 5.822244181 | -0.811204755 |
|  | 41 | Apolipoprotein E | APOE_HUMAN | APOE | 5.804308492 | -0.820342173 |
|  | 42 | Pterin-4-alpha-carbinolamine dehydratase | PHS_HUMAN | PCBD1 | 5.771595878 | -0.923575057 |
|  | 43 | NADH-ubiquinone oxidoreductase chain 2 | NU2M_HUMAN | MT-ND2 | 5.667013994 | -0.635121557 |
|  | 44 | Solute carrier family 2, facilitated glucose transporter member 1 | GTR1_HUMAN | SLC2A1 | 5.61919245 | -0.663681357 |
|  | 45 | Complement factor B | CFAB_HUMAN | CFB | 5.236649444 | -0.617495544 |
| **Comparison** | **Nr** | **Protein description** | **UniProtKB identifier** | **Gene name** | **P-value (-Log10)** | **Fold change (Log2)** |
| **T2D-PD** | 46 | Cytosolic 5'-nucleotidase 1A | 5NT1A_HUMAN | NT5C1A | 5.179974618 | -0.616858655 |
|  | 47 | Prothrombin | THRB_HUMAN | F2 | 4.820889792 | -0.609306278 |
|  | 48 | Alpha-hemoglobin-stabilizing protein | AHSP_HUMAN | AHSP | 4.35929491 | -0.712589683 |
|  | 49 | Atlastin-2 | ATLA2_HUMAN | ATL2 | 4.355193701 | -0.612198742 |
|  | 50 | Complex III assembly factor LYRM7 | LYRM7_HUMAN | LYRM7 | 4.297802595 | -0.610645945 |
|  | 51 | Complement C4-A | CO4A_HUMAN | C4A | 4.292109314 | -0.779371698 |
|  | 52 | Ankyrin-1 | ANK1_HUMAN | ANK1 | 4.248622379 | -0.618558296 |
|  | 53 | Protein 4.2 | EPB42_HUMAN | EPB42 | 4.036942152 | -0.737287086 |
|  | 54 | Spectrin alpha chain, erythrocytic 1 | SPTA1_HUMAN | SPTA1 | 3.945987757 | -0.608981211 |
|  | 55 | Isopentenyl-diphosphate Delta-isomerase 1 | IDI1_HUMAN | IDI1 | 3.405762816 | -0.854490448 |
|  | 56 | Ubiquitin carboxyl-terminal hydrolase isozyme L1 | UCHL1_HUMAN | UCHL1 | 3.379685296 | 1.080762318 |
|  | 57 | Fibronectin | FINC_HUMAN | FN1 | 3.366653526 | -0.661901303 |
|  | 58 | Ferritin heavy chain | FRIH_HUMAN | FTH1 | 2.937257584 | 0.622890449 |
|  | 59 | Sorbitol dehydrogenase | DHSO_HUMAN | SORD | 2.833855287 | -0.606820467 |
|  | 60 | Protein 4.1 | EPB41_HUMAN | EPB41 | 2.709179961 | -0.736567055 |
|  | 61 | Smoothelin | SMTN_HUMAN | SMTN | 2.460013139 | -0.705944595 |
|  | 62 | Keratin, type II cytoskeletal 5 | K2C5_HUMAN | KRT5 | 2.39043478 | 1.351229781 |
|  | 63 | Keratin, type II cytoskeletal 1 | K2C1_HUMAN | KRT1 | 2.370772979 | 0.791162071 |
|  | 64 | Malectin | MLEC_HUMAN | MLEC | 2.357759225 | 1.138926068 |
|  | 65 | Citramalyl-CoA lyase, mitochondrial | CLYBL_HUMAN | CLYBL | 2.138270875 | -0.672988281 |
|  | 66 | Protein S100-A13 | S10AD_HUMAN | S100A13 | 1.920983286 | 0.624862314 |
|  | 67 | Fibrinogen gamma chain | FIBG_HUMAN | FGG | 1.885634927 | -0.896559407 |
|  | 68 | Tryptase beta-2 | TRYB2_HUMAN | TPSB2 | 1.860222342 | 0.65589388 |
|  | 69 | Tryptase alpha/beta-1 | TRYB1_HUMAN | TPSAB1 | 1.860222342 | 0.65589388 |
|  | 70 | Fibrinogen alpha chain | FIBA_HUMAN | FGA | 1.815427091 | -0.833049372 |
|  | 71 | Keratin, type II cytoskeletal 2 epidermal | K22E_HUMAN | KRT2 | 1.777342549 | 2.79920315 |
|  | 72 | Keratin, type I cytoskeletal 10 | K1C10_HUMAN | KRT10 | 1.769528802 | 1.573059655 |
|  | 73 | Transgelin | TAGL_HUMAN | TAGLN | 1.765554546 | 1.687050419 |
|  | 74 | Cysteine-rich protein 1 | CRIP1_HUMAN | CRIP1 | 1.735589699 | 0.703789582 |
|  | 75 | Fibrinogen beta chain | FIBB_HUMAN | FGB | 1.698885088 | -0.830411812 |
|  | 76 | Decorin | PGS2_HUMAN | DCN | 1.566223649 | 0.611413235 |
|  | 77 | Filamin-A | FLNA_HUMAN | FLNA | 1.560040161 | 0.71970435 |
|  | 78 | Myosin-11 | MYH11_HUMAN | MYH11 | 1.533640686 | 0.671957823 |
|  | 79 | Prolargin | PRELP_HUMAN | PRELP | 1.394445158 | 0.748024186 |

| **Comparison** | **Nr** | **Protein description** | **UniProtKB identifier** | **Gene name** | **P-value (-Log10)** | **Fold change (Log2)** |
| --- | --- | --- | --- | --- | --- | --- |
| **PD-NG** | 1 | Palmitoyl-protein thioesterase ABHD10, mitochondrial | ABHDA_HUMAN | ABHD10 | 14.80462182 | -0.786009954 |
|  | 2 | Branched-chain-amino-acid aminotransferase, mitochondrial | BCAT2_HUMAN | BCAT2 | 14.44658574 | -0.952061254 |
|  | 3 | Small nuclear ribonucleoprotein-associated proteins B and B' | RSMB_HUMAN | SNRPB | 11.51689142 | -0.907605853 |
|  | 4 | Small nuclear ribonucleoprotein-associated protein N | RSMN_HUMAN | SNRPN | 11.51689142 | -0.907605853 |
|  | 5 | Short/branched chain specific acyl-CoA dehydrogenase, mitochondrial | ACDSB_HUMAN | ACADSB | 10.55299055 | -0.65472193 |
|  | 6 | Ras-related protein R-Ras2 | RRAS2_HUMAN | RRAS2 | 10.27202816 | -1.007417082 |
|  | 7 | Mitogen-activated protein kinase 1 | MK01_HUMAN | MAPK1 | 9.775056038 | -1.273394163 |
|  | 8 | ATP-dependent (S)-NAD(P)H-hydrate dehydratase | NNRD_HUMAN | NAXD | 9.554497055 | -0.656319341 |
|  | 9 | Pyridoxal kinase | PDXK_HUMAN | PDXK | 9.519150766 | -0.626371644 |
|  | 10 | Mitochondrial coenzyme A transporter SLC25A42 | S2542_HUMAN | SLC25A42 | 9.281309485 | -1.253543672 |
|  | 11 | Cytosolic non-specific dipeptidase | CNDP2_HUMAN | CNDP2 | 9.006448655 | -0.597967127 |
|  | 12 | 5'-deoxynucleotidase HDDC2 | HDDC2_HUMAN | HDDC2 | 8.646341293 | -0.619497116 |
|  | 13 | Histone H2B type 1-K | H2B1K_HUMAN | H2BC12 | 8.640858146 | -0.646411235 |
|  | 14 | Histone H2B type F-S | H2BFS_HUMAN | H2BC12L | 8.640858146 | -0.646411235 |
|  | 15 | Histone H2B type 1-D | H2B1D_HUMAN | H2BC5 | 8.640858146 | -0.646411235 |
|  | 16 | Histone H2B type 1-C/E/F/G/I | H2B1C_HUMAN | H2BC4 | 8.640858146 | -0.646411235 |
|  | 17 | Histone H2B type 2-F | H2B2F_HUMAN | H2BC18 | 8.640858146 | -0.646411235 |
|  | 18 | Histone H2B type 1-H | H2B1H_HUMAN | H2BC9 | 8.640858146 | -0.646411235 |
|  | 19 | Histone H2B type 1-N | H2B1N_HUMAN | H2BC15 | 8.640858146 | -0.646411235 |
|  | 20 | Histone H2B type 1-M | H2B1M_HUMAN | H2BC14 | 8.640858146 | -0.646411235 |
|  | 21 | Histone H2B type 1-L | H2B1L_HUMAN | H2BC13 | 8.640858146 | -0.646411235 |
|  | 22 | Gamma-glutamylcyclotransferase | GGCT_HUMAN | GGCT | 8.41824318 | -0.64843078 |
|  | 23 | Serine-threonine kinase receptor-associated protein | STRAP_HUMAN | STRAP | 8.212283257 | -0.612377633 |
|  | 24 | Ubiquitin-conjugating enzyme E2 L3 | UB2L3_HUMAN | UBE2L3 | 8.0281371 | -0.611517294 |
|  | 25 | Carnosine synthase 1 | CRNS1_HUMAN | CARNS1 | 7.978421138 | -0.726980115 |
|  | 26 | Tubulin beta-6 chain | TBB6_HUMAN | TUBB6 | 7.842261921 | -0.934469368 |
|  | 27 | Myosin-binding protein H | MYBPH_HUMAN | MYBPH | 7.7324673 | -2.243315362 |
|  | 28 | Caveolae-associated protein 4 | CAVN4_HUMAN | CAVIN4 | 7.575738424 | -1.122961461 |
|  | 29 | Coiled-coil domain-containing protein 72 | A0A024R1R8_HUMAN | TMA7B | 7.569354145 | -0.735321514 |
|  | 30 | Translation machinery-associated protein 7 | TMA7_HUMAN | TMA7 | 7.569354145 | -0.735321514 |
|  | 31 | Transgelin | TAGL_HUMAN | TAGLN | 7.55206409 | -1.270545922 |
|  | 32 | E3 ubiquitin-protein ligase RNF170 | RN170_HUMAN | RNF170 | 7.374809185 | -0.651418672 |
|  | 33 | 60S ribosomal protein L37a | RL37A_HUMAN | RPL37A | 7.369499632 | -0.707077321 |
|  | 34 | Heterogeneous nuclear ribonucleoprotein D0 | HNRPD_HUMAN | HNRNPD | 7.3656471 | -0.862294004 |
|  | 35 | Triadin | TRDN_HUMAN | TRDN | 7.300108856 | -0.837389961 |
|  | 36 | Caveolin-1 | CAV1_HUMAN | CAV1 | 7.23541409 | -0.713395394 |
|  | 37 | Cysteine-rich protein 1 | CRIP1_HUMAN | CRIP1 | 7.210643532 | -1.256123629 |
|  | 38 | Fatty acid-binding protein, adipocyte | FABP4_HUMAN | FABP4 | 7.075528128 | -0.644926029 |
|  | 39 | Pterin-4-alpha-carbinolamine dehydratase | PHS_HUMAN | PCBD1 | 6.93963156 | -0.916821641 |
|  | 40 | ATPase GET3 | GET3_HUMAN | GET3 | 6.829184546 | -0.597645824 |
|  | 41 | Mitogen-activated protein kinase 12 | MK12_HUMAN | MAPK12 | 6.577054287 | -0.621338152 |
|  | 42 | 14-3-3 protein eta | 1433F_HUMAN | YWHAH | 6.546656197 | -0.975880735 |
|  | 43 | V-type proton ATPase subunit B, brain isoform | VATB2_HUMAN | ATP6V1B2 | 6.542584696 | -1.11542542 |
|  | 44 | Beta-1-syntrophin | SNTB1_HUMAN | SNTB1 | 6.514907533 | -0.767757213 |
|  | 45 | Creatine kinase B-type | KCRB_HUMAN | CKB | 6.417828552 | -0.602106834 |
| **Comparison** | **Nr** | **Protein description** | **UniProtKB identifier** | **Gene name** | **P-value (-Log10)** | **Fold change (Log2)** |
| **PD-NG** | 46 | Diablo homolog, mitochondrial | DBLOH_HUMAN | DIABLO | 6.409659609 | -0.607216256 |
|  | 47 | 60S ribosomal protein L27 | RL27_HUMAN | RPL27 | 6.28531057 | -0.616194371 |
|  | 48 | Propionyl-CoA carboxylase alpha chain, mitochondrial | PCCA_HUMAN | PCCA | 5.962786786 | -0.67847377 |
|  | 49 | Myosin-11 | MYH11_HUMAN | MYH11 | 5.917065886 | -0.732632931 |
|  | 50 | 60S ribosomal protein L24 | RL24_HUMAN | RPL24 | 5.869668248 | -0.635895901 |
|  | 51 | Glutathione peroxidase 3 | GPX3_HUMAN | GPX3 | 5.846452532 | -0.587298062 |
|  | 52 | Atlastin-2 | ATLA2_HUMAN | ATL2 | 5.843094769 | -0.802657065 |
|  | 53 | Myosin light polypeptide 6 | MYL6_HUMAN | MYL6 | 5.824526189 | -0.664134132 |
|  | 54 | Moesin | MOES_HUMAN | MSN | 5.748575953 | -0.664672529 |
|  | 55 | Signal recognition particle 14 kDa protein | SRP14_HUMAN | SRP14 | 5.714309956 | -0.668526682 |
|  | 56 | Glycine cleavage system H protein, mitochondrial | GCSH_HUMAN | GCSH | 5.701315364 | -0.613187295 |
|  | 57 | Platelet-activating factor acetylhydrolase IB subunit alpha2 | PA1B2_HUMAN | PAFAH1B2 | 5.622112992 | -0.594616607 |
|  | 58 | GDH/6PGL endoplasmic bifunctional protein | G6PE_HUMAN | H6PD | 5.487727778 | -0.726699954 |
|  | 59 | Leiomodin-2 | LMOD2_HUMAN | LMOD2 | 5.14852873 | -0.622282426 |
|  | 60 | Dual specificity phosphatase 29 | DUS29_HUMAN | DUSP29 | 5.14741049 | -0.977902903 |
|  | 61 | Alpha-actinin-3 | ACTN3_HUMAN | ACTN3 | 5.127806072 | 0.841191932 |
|  | 62 | Protein phosphatase 1 regulatory subunit 7 | PP1R7_HUMAN | PPP1R7 | 5.00454507 | -0.688852917 |
|  | 63 | Aldehyde dehydrogenase family 3 member A2 | AL3A2_HUMAN | ALDH3A2 | 4.912974896 | -0.758460694 |
|  | 64 | NADH-ubiquinone oxidoreductase chain 2 | NU2M_HUMAN | MT-ND2 | 4.863180502 | -0.640604296 |
|  | 65 | Ankyrin-3 | ANK3_HUMAN | ANK3 | 4.863180502 | -0.921484572 |
|  | 66 | Heterogeneous nuclear ribonucleoprotein C-like 2 | HNRC2_HUMAN | HNRNPCL2 | 4.84467219 | -1.002694773 |
|  | 67 | Heterogeneous nuclear ribonucleoprotein C-like 3 | HNRC3_HUMAN | HNRNPCL3 | 4.84467219 | -1.002694773 |
|  | 68 | Heterogeneous nuclear ribonucleoprotein C-like 1 | HNRC1_HUMAN | HNRNPCL1 | 4.84467219 | -1.002694773 |
|  | 69 | Heterogeneous nuclear ribonucleoproteins C1/C2 | HNRPC_HUMAN | HNRNPC | 4.84467219 | -1.002694773 |
|  | 70 | Heterogeneous nuclear ribonucleoprotein C-like 4 | HNRC4_HUMAN | HNRNPC4 | 4.84467219 | -1.002694773 |
|  | 71 | Ras GTPase-activating protein 4B | RAS4B_HUMAN | RASA4B | 4.744112939 | -1.279355384 |
|  | 72 | Ras GTPase-activating protein 4 | RASL2_HUMAN | RASA4 | 4.744112939 | -1.279355384 |
|  | 73 | Keratin, type II cytoskeletal 2 epidermal | K22E_HUMAN | KRT2 | 4.68242773 | -1.427758809 |
|  | 74 | Sarcolemmal membrane-associated protein | SLMAP_HUMAN | SLMAP | 4.669254108 | -0.630273496 |
|  | 75 | Protein S100-A4 | S10A4_HUMAN | S100A4 | 4.578718955 | -0.685455012 |
|  | 76 | Endoplasmin | ENPL_HUMAN | HSP90B1 | 4.511715856 | -0.608952968 |
|  | 77 | Actin, aortic smooth muscle | ACTA_HUMAN | ACTA2 | 4.346150446 | -0.871578548 |
|  | 78 | Nucleophosmin | NPM_HUMAN | NPM1 | 4.326280779 | -0.669964717 |
|  | 79 | 40S ribosomal protein S26 | RS26_HUMAN | RPS26 | 4.323014483 | -0.622265124 |
|  | 80 | ATP-dependent RNA helicase DDX19A | DD19A_HUMAN | DDX19A | 4.29446118 | -0.656927838 |
|  | 81 | Complement C4-A | CO4A_HUMAN | C4A | 4.093046477 | 0.707511147 |
|  | 82 | Serine/threonine-protein kinase Nek7 | NEK7_HUMAN | NEK7 | 4.033292032 | -0.603330243 |
|  | 83 | Myelin P2 protein | MYP2_HUMAN | PMP2 | 4.002641816 | -0.618875501 |
|  | 84 | Prostaglandin E synthase 3 | TEBP_HUMAN | PTGES3 | 3.864178988 | -0.887438887 |
|  | 85 | Apolipoprotein C-I | APOC1_HUMAN | APOC1 | 3.696639647 | 0.726226626 |
|  | 86 | 60S ribosomal protein L21 | RL21_HUMAN | RPL21 | 3.652345448 | -0.794169088 |
|  | 87 | Annexin A1 | ANXA1_HUMAN | ANXA1 | 3.126736936 | -0.716307942 |
|  | 88 | Dermatopontin | DERM_HUMAN | DPT | 2.966084554 | -0.819788313 |
|  | 89 | Protein S100-A13 | S10AD_HUMAN | S100A13 | 2.799322551 | -1.325911534 |
|  | 90 | Alpha-aminoadipic semialdehyde dehydrogenase | AL7A1_HUMAN | ALDH7A1 | 2.38282314 | -0.591216892 |

| **Comparison** | **Nr** | **Protein description** | **UniProtKB identifier** | **Gene name** | **P-value (-Log10)** | **Fold change (Log2)** |
| --- | --- | --- | --- | --- | --- | --- |
| **PD-NG** | 91 | Ubiquitin carboxyl-terminal hydrolase isozyme L1 | UCHL1_HUMAN | UCHL1 | 2.262939241 | -0.75476404 |
|  | 92 | Decorin | PGS2_HUMAN | DCN | 1.843003225 | -0.689529937 |
|  | 93 | Immunoglobulin heavy constant gamma 4 | IGHG4_HUMAN | IGHG4 | 1.804706402 | 0.610805402 |
|  | 94 | Dihydropyrimidinase-related protein 2 | DPYL2_HUMAN | DPYSL2 | 1.785442779 | -2.286481127 |
|  | 95 | Mimecan | MIME_HUMAN | OGN | 1.592690909 | -0.767386643 |
|  | 96 | Prolargin | PRELP_HUMAN | PRELP | 1.530951891 | -0.905487232 |
|  | 97 | Keratin, type II cytoskeletal 5 | K2C5_HUMAN | KRT5 | 1.486394265 | -0.644253882 |
|  | 98 | Catechol O-methyltransferase domain-containing protein 1 | CMTD1_HUMAN | COMTD1 | 1.376303831 | -0.70819504 |

| **Comparison** | **Nr** | **Protein description** | **UniProtKB identifier** | **Gene name** | **P-value (-Log10)** | **Fold change (Log2)** |
| --- | --- | --- | --- | --- | --- | --- |
| **PD-NG, T2D-NG** | 1 | Palmitoyl-protein thioesterase ABHD10, mitochondrial | ABHDA_HUMAN | ABHD10 | 14.80462182 | -0.786009954 |
|  |  |  |  |  | 18.29771775 | -0.788447684 |
|  | 2 | Short/branched chain specific acyl-CoA dehydrogenase, mitochondrial | ACDSB_HUMAN | ACADSB | 10.55299055 | -0.65472193 |
|  |  |  |  |  | 10.17865873 | -0.632994083 |
|  | 3 | Alpha-actinin-3 | ACTN3_HUMAN | ACTN3 | 5.127806072 | 0.841191932 |
|  |  |  |  |  | 6.336388514 | 1.264340383 |
|  | 4 | Aldehyde dehydrogenase family 3 member A2 | AL3A2_HUMAN | ALDH3A2 | 4.912974896 | -0.758460694 |
|  |  |  |  |  | 6.185764673 | -0.763288628 |
|  | 5 | Alpha-aminoadipic semialdehyde dehydrogenase | AL7A1_HUMAN | ALDH7A1 | 2.38282314 | -0.591216892 |
|  |  |  |  |  | 3.393868265 | -0.859557194 |
|  | 6 | V-type proton ATPase subunit B, brain isoform | VATB2_HUMAN | ATP6V1B2 | 6.542584696 | -1.11542542 |
|  |  |  |  |  | 6.70359078 | -1.225687611 |
|  | 7 | Branched-chain-amino-acid aminotransferase, mitochondrial | BCAT2_HUMAN | BCAT2 | 14.44658574 | -0.952061254 |
|  |  |  |  |  | 12.14032855 | -0.797862155 |
|  | 8 | Carnosine synthase 1 | CRNS1_HUMAN | CARNS1 | 7.978421138 | -0.726980115 |
|  |  |  |  |  | 13.71135298 | -0.891231292 |
|  | 9 | Caveolin-1 | CAV1_HUMAN | CAV1 | 7.23541409 | -0.713395394 |
|  |  |  |  |  | 11.86963459 | -0.929824035 |
|  | 10 | Caveolae-associated protein 4 | CAVN4_HUMAN | CAVIN4 | 7.575738424 | -1.122961461 |
|  |  |  |  |  | 10.61441718 | -1.301013643 |
|  | 11 | Cytosolic non-specific dipeptidase | CNDP2_HUMAN | CNDP2 | 9.006448655 | -0.597967127 |
|  |  |  |  |  | 9.077715724 | -0.730457486 |
|  | 12 | Catechol O-methyltransferase domain-containing protein 1 | CMTD1_HUMAN | COMTD1 | 1.376303831 | -0.70819504 |
|  |  |  |  |  | 4.297323646 | -0.852577943 |
|  | 13 | ATP-dependent RNA helicase DDX19A | DD19A_HUMAN | DDX19A | 4.29446118 | -0.656927838 |
|  |  |  |  |  | 9.15026453 | -1.190137999 |
|  | 14 | Diablo homolog, mitochondrial | DBLOH_HUMAN | DIABLO | 6.409659609 | -0.607216256 |
|  |  |  |  |  | 13.21946929 | -0.667286317 |
|  | 15 | Dermatopontin | DERM_HUMAN | DPT | 2.966084554 | -0.819788313 |
|  |  |  |  |  | 3.862423216 | -1.309915988 |
|  | 16 | Glycine cleavage system H protein, mitochondrial | GCSH_HUMAN | GCSH | 5.701315364 | -0.613187295 |
|  |  |  |  |  | 11.00005746 | -0.98772039 |
|  | 17 | Gamma-glutamylcyclotransferase | GGCT_HUMAN | GGCT | 8.41824318 | -0.64843078 |
|  |  |  |  |  | 14.26914603 | -1.083610233 |
|  | 18 | GDH/6PGL endoplasmic bifunctional protein | G6PE_HUMAN | H6PD | 5.487727778 | -0.726699954 |
|  |  |  |  |  | 13.50928296 | -0.832360405 |
|  | 19 | 5'-deoxynucleotidase HDDC2 | HDDC2_HUMAN | HDDC2 | 8.646341293 | -0.619497116 |
|  |  |  |  |  | 11.4831995 | -0.816702735 |
|  | 20 | Heterogeneous nuclear ribonucleoprotein D0 | HNRPD_HUMAN | HNRNPD | 7.3656471 | -0.862294004 |
|  |  |  |  |  | 9.941543329 | -1.187424382 |
|  | 21 | Endoplasmin | ENPL_HUMAN | HSP90B1 | 4.511715856 | -0.608952968 |
|  |  |  |  |  | 7.733747477 | -1.092729185 |
|  | 22 | Leiomodin-2 | LMOD2_HUMAN | LMOD2 | 5.14852873 | -0.622282426 |
|  |  |  |  |  | 7.761822227 | -0.680936038 |
| **Comparison** | **Nr** | **Protein description** | **UniProtKB identifier** | **Gene name** | **P-value (-Log10)** | **Fold change (Log2)** |
| **PD-NG, T2D-NG** | 23 | Mitogen-activated protein kinase 1 | MK01_HUMAN | MAPK1 | 9.775056038 | -1.273394163 |
|  |  |  |  |  | 10.7514891 | -1.093732029 |
|  | 24 | Mitogen-activated protein kinase 12 | MK12_HUMAN | MAPK12 | 6.577054287 | -0.621338152 |
|  |  |  |  |  | 8.186683792 | -0.625970626 |
|  | 25 | Moesin | MOES_HUMAN | MSN | 5.748575953 | -0.664672529 |
|  |  |  |  |  | 10.76632119 | -1.090704746 |
|  | 26 | Myosin-binding protein H | MYBPH_HUMAN | MYBPH | 7.7324673 | -2.243315362 |
|  |  |  |  |  | 7.60537302 | -2.196416429 |
|  | 27 | ATP-dependent (S)-NAD(P)H-hydrate dehydratase | NNRD_HUMAN | NAXD | 9.554497055 | -0.656319341 |
|  |  |  |  |  | 12.33312723 | -0.830381724 |
|  | 28 | Serine/threonine-protein kinase Nek7 | NEK7_HUMAN | NEK7 | 4.033292032 | -0.603330243 |
|  |  |  |  |  | 10.61290582 | -1.081864276 |
|  | 29 | Nucleophosmin | NPM_HUMAN | NPM1 | 4.326280779 | -0.669964717 |
|  |  |  |  |  | 7.347246131 | -1.087475935 |
|  | 30 | Platelet-activating factor acetylhydrolase IB subunit alpha2 | PA1B2_HUMAN | PAFAH1B2 | 5.622112992 | -0.594616607 |
|  |  |  |  |  | 12.51593184 | -1.098059225 |
|  | 31 | Propionyl-CoA carboxylase alpha chain, mitochondrial | PCCA_HUMAN | PCCA | 5.962786786 | -0.67847377 |
|  |  |  |  |  | 8.247319744 | -0.760508651 |
|  | 32 | Pyridoxal kinase | PDXK_HUMAN | PDXK | 9.519150766 | -0.626371644 |
|  |  |  |  |  | 11.50132694 | -0.788552585 |
|  | 33 | Prostaglandin E synthase 3 | TEBP_HUMAN | PTGES3 | 3.864178988 | -0.887438887 |
|  |  |  |  |  | 8.81325385 | -1.286301627 |
|  | 34 | E3 ubiquitin-protein ligase RNF170 | RN170_HUMAN | RNF170 | 7.374809185 | -0.651418672 |
|  |  |  |  |  | 15.50276471 | -1.133136989 |
|  | 35 | 60S ribosomal protein L27 | RL27_HUMAN | RPL27 | 6.28531057 | -0.616194371 |
|  |  |  |  |  | 12.99482915 | -1.048560296 |
|  | 36 | 60S ribosomal protein L37a | RL37A_HUMAN | RPL37A | 7.369499632 | -0.707077321 |
|  |  |  |  |  | 17.58708657 | -1.277950481 |
|  | 37 | 40S ribosomal protein S26 | RS26_HUMAN | RPS26 | 4.323014483 | -0.622265124 |
|  |  |  |  |  | 15.66982109 | -0.940566388 |
|  | 38 | Ras-related protein R-Ras2 | RRAS2_HUMAN | RRAS2 | 10.27202816 | -1.007417082 |
|  |  |  |  |  | 13.66375712 | -1.473026972 |
|  | 39 | Protein S100-A4 | S10A4_HUMAN | S100A4 | 4.578718955 | -0.685455012 |
|  |  |  |  |  | 9.020578933 | -1.122617243 |
|  | 40 | Mitochondrial coenzyme A transporter SLC25A42 | S2542_HUMAN | SLC25A42 | 9.281309485 | -1.253543672 |
|  |  |  |  |  | 13.55022806 | -1.604800895 |
|  | 41 | Sarcolemmal membrane-associated protein | SLMAP_HUMAN | SLMAP | 4.669254108 | -0.630273496 |
|  |  |  |  |  | 12.37571557 | -1.184183147 |
|  | 42 | Small nuclear ribonucleoprotein-associated proteins B and B' | RSMB_HUMAN | SNRPB | 11.51689142 | -0.907605853 |
|  |  |  |  |  | 17.64725489 | -1.163587044 |
|  | 43 | Small nuclear ribonucleoprotein-associated protein N | RSMN_HUMAN | SNRPN | 11.51689142 | -0.907605853 |
|  |  |  |  |  | 17.64725489 | -1.163587044 |
|  | 44 | Beta-1-syntrophin | SNTB1_HUMAN | SNTB1 | 6.514907533 | -0.767757213 |
|  |  |  |  |  | 7.978421138 | -0.826789672 |
| **Comparison** | **Nr** | **Protein description** | **UniProtKB identifier** | **Gene name** | **P-value (-Log10)** | **Fold change (Log2)** |
| **PD-NG, T2D-NG** | 45 | Signal recognition particle 14 kDa protein | SRP14_HUMAN | SRP14 | 5.714309956 | -0.668526682 |
|  |  |  |  |  | 7.308053295 | -0.812674525 |
|  | 46 | Serine-threonine kinase receptor-associated protein | STRAP_HUMAN | STRAP | 8.212283257 | -0.612377633 |
|  |  |  |  |  | 9.491837439 | -0.65120016 |
|  | 47 | Translation machinery-associated protein 7 | TMA7_HUMAN | TMA7 | 7.569354145 | -0.735321514 |
|  |  |  |  |  | 9.840656756 | -1.105470271 |
|  | 48 | Coiled-coil domain-containing protein 72 | A0A024R1R8_HUMAN | TMA7B | 7.569354145 | -0.735321514 |
|  |  |  |  |  | 9.840656756 | -1.105470271 |
|  | 49 | Tubulin beta-6 chain | TBB6_HUMAN | TUBB6 | 7.842261921 | -0.934469368 |
|  |  |  |  |  | 11.90748284 | -1.151921726 |
|  | 50 | Ubiquitin-conjugating enzyme E2 L3 | UB2L3_HUMAN | UBE2L3 | 8.0281371 | -0.611517294 |
|  |  |  |  |  | 17.69029388 | -1.097520104 |
|  | 51 | 14-3-3 protein eta | 1433F_HUMAN | YWHAH | 6.546656197 | -0.975880735 |
|  |  |  |  |  | 6.344258617 | -0.975157108 |

| **Comparison** | **Nr** | **Protein description** | **UniProtKB identifier** | **Gene name** | **P-value (-Log10)** | **Fold change (Log2)** |
| --- | --- | --- | --- | --- | --- | --- |
| **PD-NG, T2D-NG** | 1 | Acetyl-coenzyme A synthetase 2-like, mitochondrial | ACS2L_HUMAN | ACSS1 | 17.64725489 | -1.214285633 |
|  |  |  |  |  | 13.80611311 | -0.87785461 |
|  | 2 | Alpha-hemoglobin-stabilizing protein | AHSP_HUMAN | AHSP | 5.012097604 | -0.978119632 |
|  |  |  |  |  | 4.35929491 | -0.712589683 |
|  | 3 | Ankyrin-1 | ANK1_HUMAN | ANK1 | 4.105368653 | -0.621269381 |
|  |  |  |  |  | 4.248622379 | -0.618558296 |
|  | 4 | Apolipoprotein E | APOE_HUMAN | APOE | 9.090665384 | -0.710119078 |
|  |  |  |  |  | 5.804308492 | -0.820342173 |
|  | 5 | Biliverdin reductase A | BIEA_HUMAN | BLVRA | 10.79152866 | -0.955079005 |
|  |  |  |  |  | 8.843423097 | -0.750134485 |
|  | 6 | Bisphosphoglycerate mutase | PMGE_HUMAN | BPGM | 10.35990461 | -1.047747983 |
|  |  |  |  |  | 9.077715724 | -0.721776985 |
|  | 7 | Complement C4-B | CO4B_HUMAN | C4B; C4B_2 | 14.77871595 | -1.380211931 |
|  |  |  |  |  | 10.17865873 | -0.809326958 |
|  | 8 | Complement factor B | CFAB_HUMAN | CFB | 5.070643344 | -0.614617412 |
|  |  |  |  |  | 5.236649444 | -0.617495544 |
|  | 9 | Charged multivesicular body protein 4b | CHM4B_HUMAN | CHMP4B | 9.771127409 | -1.669911639 |
|  |  |  |  |  | 8.281005885 | -1.234665395 |
|  | 10 | Citramalyl-CoA lyase, mitochondrial | CLYBL_HUMAN | CLYBL | 5.817825829 | -1.12608342 |
|  |  |  |  |  | 2.138270875 | -0.672988281 |
|  | 11 | Crk-like protein | CRKL_HUMAN | CRKL | 15.56324563 | -1.31604401 |
|  |  |  |  |  | 9.667703065 | -0.894180317 |
|  | 12 | Protein 4.1 | EPB41_HUMAN | EPB41 | 7.102440648 | -1.301451253 |
|  |  |  |  |  | 2.709179961 | -0.736567055 |
|  | 13 | Protein 4.2 | EPB42_HUMAN | EPB42 | 5.039754952 | -0.79872249 |
|  |  |  |  |  | 4.036942152 | -0.737287086 |
|  | 14 | Fibrinogen gamma chain | FIBG_HUMAN | FGG | 2.118787766 | -0.654903853 |
|  |  |  |  |  | 1.885634927 | -0.896559407 |
|  | 15 | COP9 signalosome complex subunit 1 | CSN1_HUMAN | GPS1 | 19.22517583 | -1.424666345 |
|  |  |  |  |  | 10.37751831 | -0.885147739 |
|  | 16 | Porphobilinogen deaminase | HEM3_HUMAN | HMBS | 11.08594913 | -0.776192424 |
|  |  |  |  |  | 9.499900728 | -0.635711722 |
|  | 17 | Isopentenyl-diphosphate Delta-isomerase 1 | IDI1_HUMAN | IDI1 | 5.838538742 | -1.243696484 |
|  |  |  |  |  | 3.405762816 | -0.854490448 |
|  | 18 | Immunoglobulin heavy constant gamma 3 | IGHG3_HUMAN | IGHG3 | 7.712476895 | -1.080352986 |
|  |  |  |  |  | 7.556185183 | -0.943908046 |
|  | 19 | Inositol monophosphatase 1 | IMPA1_HUMAN | IMPA1 | 18.70510909 | -1.523157482 |
|  |  |  |  |  | 11.78574598 | -1.111595725 |
|  | 20 | Inter-alpha-trypsin inhibitor heavy chain H4 | ITIH4_HUMAN | ITIH4 | 8.601354626 | -0.670509479 |
|  |  |  |  |  | 6.802338247 | -0.806655788 |
|  | 21 | Complex III assembly factor LYRM7 | LYRM7_HUMAN | LYRM7 | 11.23613516 | -1.025692015 |
|  |  |  |  |  | 4.297802595 | -0.610645945 |
|  | 22 | Methylmalonyl-CoA epimerase, mitochondrial | MCEE_HUMAN | MCEE | 14.80462182 | -1.253817287 |
|  |  |  |  |  | 7.421893538 | -0.706832907 |
| **Comparison** | **Nr** | **Protein description** | **UniProtKB identifier** | **Gene name** | **P-value (-Log10)** | **Fold change (Log2)** |
| **PD-NG, T2D-NG** | 23 | NIF3-like protein 1 | NIF3L_HUMAN | NIF3L1 | 13.38510065 | -1.144693678 |
|  |  |  |  |  | 8.213465756 | -0.630273869 |
|  | 24 | Palladin | PALLD_HUMAN | PALLD | 17.43354153 | -1.080176724 |
|  |  |  |  |  | 13.977845 | -0.71638643 |
|  | 25 | 6-phosphogluconate dehydrogenase, decarboxylating | 6PGD_HUMAN | PGD | 11.25714635 | -0.701180046 |
|  |  |  |  |  | 8.565046689 | -0.655956491 |
|  | 26 | 60S ribosomal protein L28 | RL28_HUMAN | RPL28 | 14.84619115 | -1.08843952 |
|  |  |  |  |  | 11.52004438 | -0.757258161 |
|  | 27 | 40S ribosomal protein S6 | RS6_HUMAN | RPS6 | 12.7029841 | -1.291094916 |
|  |  |  |  |  | 9.290310332 | -0.847154598 |
|  | 28 | Solute carrier family 2, facilitated glucose transporter member 1 | GTR1_HUMAN  GTR1_HUMAN | SLC2A1  SLC2A1 | 4.923233941 | -0.726410213 |
|  |  |  |  |  | 5.61919245 | -0.663681357 |
|  | 29 | Smoothelin | SMTN_HUMAN | SMTN | 3.059816284 | -0.683961232 |
|  |  |  |  |  | 2.460013139 | -0.705944595 |
|  | 30 | Alpha-synuclein | SYUA_HUMAN | SNCA | 5.169969438 | -0.980565549 |
|  |  |  |  |  | 5.822244181 | -0.811204755 |
|  | 31 | Stromal interaction molecule 1 | STIM1_HUMAN | STIM1 | 16.0362008 | -1.246878757 |
|  |  |  |  |  | 7.574111692 | -0.67031369 |
|  | 32 | Thioredoxin, mitochondrial | THIOM_HUMAN | TXN2 | 3.037604085 | -1.489449457 |
|  |  |  |  |  | 11.73453909 | -0.923694368 |
|  | 33 | Vacuolar protein sorting-associated protein 28 homolog | VPS28_HUMAN | VPS28 | 16.51739883 | -1.101185895 |
|  |  |  |  |  | 6.528811319 | -0.810346411 |
| **PD-NG, T2D-PD** | 1 | Apolipoprotein C-I | APOC1_HUMAN | APOC1 | 3.696639647 | 0.726226626 |
|  |  |  |  |  | 7.08982096 | -1.141216963 |
|  | 2 | Complement C4-A | CO4A_HUMAN | C4A | 4.093046477 | 0.707511147 |
|  |  |  |  |  | 4.292109314 | -0.779371698 |
|  | 3 | Cysteine-rich protein 1 | CRIP1_HUMAN | CRIP1 | 7.210643532 | -1.256123629 |
|  |  |  |  |  | 1.735589699 | 0.703789582 |
|  | 4 | Decorin | PGS2_HUMAN | DCN | 1.843003225 | -0.689529937 |
|  |  |  |  |  | 1.566223649 | 0.611413235 |
|  | 5 | Keratin, type II cytoskeletal 2 epidermal | K22E_HUMAN | KRT2 | 4.68242773 | -1.427758809 |
|  |  |  |  |  | 1.777342549 | 2.79920315 |
|  | 6 | Keratin, type II cytoskeletal 5 | K2C5_HUMAN | KRT5 | 1.486394265 | -0.644253882 |
|  |  |  |  |  | 2.39043478 | 1.351229781 |
|  | 7 | Myosin-11 | MYH11_HUMAN | MYH11 | 5.917065886 | -0.732632931 |
|  |  |  |  |  | 1.533640686 | 0.671957823 |
|  | 8 | Prolargin | PRELP_HUMAN | PRELP | 1.530951891 | -0.905487232 |
|  |  |  |  |  | 1.394445158 | 0.748024186 |
|  | 9 | Protein S100-A13 | S10AD_HUMAN | S100A13 | 2.799322551 | -1.325911534 |
|  |  |  |  |  | 1.920983286 | 0.624862314 |
|  | 10 | Transgelin | TAGL_HUMAN | TAGLN | 7.55206409 | -1.270545922 |
|  |  |  |  |  | 1.765554546 | 1.687050419 |
|  | 11 | Ubiquitin carboxyl-terminal hydrolase isozyme L1 | UCHL1_HUMAN | UCHL1 | 2.262939241 | -0.75476404 |
|  |  |  |  |  | 3.379685296 | 1.080762318 |
| **Comparison** | **Nr** | **Protein description** | **UniProtKB identifier** | **Gene name** | **P-value (-Log10)** | **Fold change (Log2)** |
| **PD-NG, T2D-NG, T2D-PD** | 1 | Ankyrin-3 | ANK3_HUMAN | ANK3 | 4.863180502 | -0.921484572 |
|  |  |  |  |  | 12.18183096 | -1.675415176 |
|  |  |  |  |  | 9.635039511 | -0.753930604 |
|  | 2 | Atlastin-2 | ATLA2_HUMAN | ATL2 | 5.843094769 | -0.802657065 |
|  |  |  |  |  | 12.78456688 | -1.414855807 |
|  |  |  |  |  | 4.355193701 | -0.612198742 |
|  | 3 | Dihydropyrimidinase-related protein 2 | DPYL2_HUMAN | DPYSL2 | 1.785442779 | -2.286481127 |
|  |  |  |  |  | 3.345708469 | -3.608427536 |
|  |  |  |  |  | 10.55299055 | -1.321946408 |
|  | 4 | Dual specificity phosphatase 29 | DUS29_HUMAN | DUSP29 | 5.14741049 | -0.977902903 |
|  |  |  |  |  | 12.6981119 | -1.956593834 |
|  |  |  |  |  | 8.716869691 | -0.978690931 |
|  | 5 | ATPase GET3 | GET3_HUMAN | GET3 | 6.829184546 | -0.597645824 |
|  |  |  |  |  | 18.62478379 | -1.393925988 |
|  |  |  |  |  | 7.266113682 | -0.796280164 |
|  | 6 | Heterogeneous nuclear ribonucleoproteins C1/C2 | HNRPC_HUMAN | HNRNPC | 4.84467219 | -1.002694773 |
|  |  |  |  |  | 8.706531097 | -1.733076965 |
|  |  |  |  |  | 10.67234189 | -0.730382192 |
|  | 7 | Heterogeneous nuclear ribonucleoprotein C-like 4 | HNRC4_HUMAN | HNRNPC4 | 4.84467219 | -1.002694773 |
|  |  |  |  |  | 8.706531097 | -1.733076965 |
|  |  |  |  |  | 10.67234189 | -0.730382192 |
|  | 8 | Heterogeneous nuclear ribonucleoprotein C-like 1 | HNRC1_HUMAN | HNRNPCL1 | 4.84467219 | -1.002694773 |
|  |  |  |  |  | 8.706531097 | -1.733076965 |
|  |  |  |  |  | 10.67234189 | -0.730382192 |
|  | 9 | Heterogeneous nuclear ribonucleoprotein C-like 2 | HNRC2_HUMAN | HNRNPCL2 | 4.84467219 | -1.002694773 |
|  |  |  |  |  | 8.706531097 | -1.733076965 |
|  |  |  |  |  | 10.67234189 | -0.730382192 |
|  | 10 | Heterogeneous nuclear ribonucleoprotein C-like 3 | HNRC3_HUMAN | HNRNPCL3 | 4.84467219 | -1.002694773 |
|  |  |  |  |  | 8.706531097 | -1.733076965 |
|  |  |  |  |  | 10.67234189 | -0.730382192 |
|  | 11 | NADH-ubiquinone oxidoreductase chain 2 | NU2M_HUMAN | MT-ND2 | 4.863180502 | -0.640604296 |
|  |  |  |  |  | 13.71135298 | -1.275725853 |
|  |  |  |  |  | 5.667013994 | -0.635121557 |
|  | 12 | Pterin-4-alpha-carbinolamine dehydratase | PHS_HUMAN | PCBD1 | 6.93963156 | -0.916821641 |
|  |  |  |  |  | 14.89186623 | -1.840396697 |
|  |  |  |  |  | 5.771595878 | -0.923575057 |
|  | 13 | Protein phosphatase 1 regulatory subunit 7 | PP1R7_HUMAN | PPP1R7 | 5.00454507 | -0.688852917 |
|  |  |  |  |  | 13.11180557 | -1.495216811 |
|  |  |  |  |  | 6.231388224 | -0.806363894 |
|  | 14 | Ras GTPase-activating protein 4 | RASL2_HUMAN | RASA4 | 4.744112939 | -1.279355384 |
|  |  |  |  |  | 9.993111309 | -2.266477549 |
|  |  |  |  |  | 16.29689172 | -0.987122164 |
|  | 15 | Ras GTPase-activating protein 4B | RAS4B_HUMAN | RASA4B | 4.744112939 | -1.279355384 |
|  |  |  |  |  | 9.993111309 | -2.266477549 |
|  |  |  |  |  | 16.29689172 | -0.987122164 |
| **Comparison** | **Nr** | **Protein description** | **UniProtKB identifier** | **Gene name** | **P-value (-Log10)** | **Fold change (Log2)** |
| **PD-NG, T2D-NG, T2D-PD** | 16 | 60S ribosomal protein L21 | RL21_HUMAN | RPL21 | 3.652345448 | -0.794169088 |
|  |  |  |  |  | 5.822244181 | -1.599401797 |
|  |  |  |  |  | 10.759585 | -0.805232709 |
|  | 17 | Triadin | TRDN_HUMAN | TRDN | 7.300108856 | -0.837389961 |
|  |  |  |  |  | 16.69161816 | -1.630607801 |
|  |  |  |  |  | 10.58623291 | -0.79321784 |

# Appendix G: Proteins identified by the REHA algorithm in Figure 6

The table in Appendix E enriches Figure 6 by providing further details on proteins identified by the REHA algorithm as significant for classification tasks between merged tested groups. It contains a detailed list that includes the comparison groups involved, the occurrence frequency of each protein within the study, a description of each protein, its unique UniProtKB identifier, and the gene name associated with each protein.

| **Comparison** | **Occurence** | **Protein description** | **UniProtKB identifier** | **Gene name** |
| --- | --- | --- | --- | --- |
| **PD-NG** | 25 | Protein preY, mitochondrial | PREY_HUMAN | PYURF |
|  | 22 | 60S acidic ribosomal protein P2 | RLA2_HUMAN | RPLP2 |
|  | 16 | Short-chain specific acyl-CoA dehydrogenase, mitochondrial | ACADS_HUMAN | ACADS |
|  | 14 | cAMP-dependent protein kinase type II-alpha regulatory subunit | KAP2_HUMAN | PRKAR2A |
|  | 12 | Serine/threonine-protein kinase OSR1 | OXSR1_HUMAN | OXSR1 |
|  | 12 | ATP-binding cassette sub-family F member 1 | ABCF1_HUMAN | ABCF1 |
|  | 12 | Troponin C, slow skeletal and cardiac muscles | TNNC1_HUMAN | TNNC1 |
|  | 10 | 26S proteasome regulatory subunit 7 | PRS7_HUMAN | PSMC2 |
|  | 10 | ATP-dependent (S)-NAD(P)H-hydrate dehydratase | NNRD_HUMAN | NAXD |
|  | 9 | Ras-related protein Rab-21 | RAB21_HUMAN | RAB21 |
|  | 9 | Creatine kinase M-type | KCRM_HUMAN | CKM |
|  | 9 | ATP synthase subunit d, mitochondrial | ATP5H_HUMAN | ATP5PD |
|  | 8 | Pterin-4-alpha-carbinolamine dehydratase | PHS_HUMAN | PCBD1 |
|  | 7 | Fibrillin-1 | FBN1_HUMAN | FBN1 |
|  | 7 | 60S ribosomal protein L30 | RL30_HUMAN | RPL30 |
|  | 6 | Mannose-6-phosphate isomerase | MPI_HUMAN | MPI |
|  | 6 | Cullin-associated NEDD8-dissociated protein 1 | CAND1_HUMAN | CAND1 |
|  | 5 | 40S ribosomal protein S19 | RS19_HUMAN | RPS19 |
|  | 4 | All-trans-retinol dehydrogenase [NAD(+)] ADH1B | ADH1B_HUMAN | ADH1B |
|  | 4 | Hemoglobin subunit alpha | HBA_HUMAN | HBA1; HBA2 |
|  | 4 | GMP reductase 1 | GMPR1_HUMAN | GMPR |
|  | 3 | NADH dehydrogenase [ubiquinone] 1 subunit C2 | NDUC2_HUMAN | NDUFC2 |
|  | 3 | Leucine-rich PPR motif-containing protein, mitochondrial | LPPRC_HUMAN | LRPPRC |
|  | 3 | Adenylyl cyclase-associated protein 1 | CAP1_HUMAN | CAP1 |
|  | 3 | Annexin A4 | ANXA4_HUMAN | ANXA4 |
|  | 3 | Dihydrolipoyl dehydrogenase, mitochondrial | DLDH_HUMAN | DLD |
|  | 3 | 14-3-3 protein gamma | 1433G_HUMAN | YWHAG |
|  | 2 | Starch-binding domain-containing protein 1 | STBD1_HUMAN | STBD1 |
|  | 2 | ATP synthase subunit beta, mitochondrial | ATPB_HUMAN | ATP5F1B |
|  | 2 | Ferritin heavy chain | FRIH_HUMAN | FTH1 |
|  | 2 | Glutaredoxin-1 | GLRX1_HUMAN | GLRX |
|  | 2 | 40S ribosomal protein S15a | RS15A_HUMAN | RPS15A |
|  | 2 | Carboxymethylenebutenolidase homolog | CMBL_HUMAN | CMBL |
|  | 2 | Transaldolase | TALDO_HUMAN | TALDO1 |
|  | 2 | Eukaryotic translation initiation factor 5 | IF5_HUMAN | EIF5 |
|  | 2 | Electrogenic aspartate/glutamate antiporter SLC25A12, mitochondrial | S2512_HUMAN | SLC25A12 |
|  | 2 | Cytochrome c oxidase subunit 2 | COX2_HUMAN | MT-CO2 |
|  | 2 | Monocarboxylate transporter 4 | MOT4_HUMAN | SLC16A3 |
|  | 2 | Isocitrate dehydrogenase [NADP] cytoplasmic | IDHC_HUMAN | IDH1 |
|  | 2 | Fibrinogen gamma chain | FIBG_HUMAN | FGG |
|  | 2 | Lactoylglutathione lyase | LGUL_HUMAN | GLO1 |
|  | 2 | Importin subunit alpha-3 | IMA3_HUMAN | KPNA4 |
|  | 2 | Charged multivesicular body protein 4b | CHM4B_HUMAN | CHMP4B |
|  | 2 | Cytosolic 5'-nucleotidase 1A | 5NT1A_HUMAN | NT5C1A |
|  | 2 | ATP synthase protein 8 | ATP8_HUMAN | MT-ATP8 |
|  | 2 | Dual specificity protein phosphatase 3 | DUS3_HUMAN | DUSP3 |
|  | 2 | Nucleoredoxin | NXN_HUMAN | NXN |
|  | 2 | Leiomodin-3 | LMOD3_HUMAN | LMOD3 |

| **Comparison** | **Occurence** | **Protein description** | **UniProtKB identifier** | **Gene name** |
| --- | --- | --- | --- | --- |
| **PD-NG,  T2D-NG** | 17 | Ras-related protein Rab-21 | RAB21_HUMAN | RAB21 |
|  | 16 | Adenylyl cyclase-associated protein 1 | CAP1_HUMAN | CAP1 |
|  | 16 | GMP reductase 1 | GMPR1_HUMAN | GMPR |
|  | 15 | Monocarboxylate transporter 4 | MOT4_HUMAN | SLC16A3 |
|  | 14 | ATP synthase protein 8 | ATP8_HUMAN | MT-ATP8 |
|  | 5 | Dihydrolipoyl dehydrogenase, mitochondrial | DLDH_HUMAN | DLD |
| **T2D-NG,  T2D-PD** | 24 | 40S ribosomal protein S12 | RS12_HUMAN | RPS12 |
|  | 19 | ATP-dependent 6-phosphofructokinase, muscle type | PFKAM_HUMAN | PFKM |
|  | 11 | Tubulin beta-4B chain | TBB4B_HUMAN | TUBB4B |
|  | 9 | EH domain-binding protein 1-like protein 1 | EH1L1_HUMAN | EHBP1L1 |
|  | 4 | PDZ and LIM domain protein 1 | PDLI1_HUMAN | PDLIM1 |
|  | 2 | Mitochondrial import inner membrane translocase subunit TIM44 | TIM44_HUMAN | TIMM44 |
| **PD-NG,  T2D-PD** | 23 | 60S acidic ribosomal protein P2 | RLA2_HUMAN | RPLP2 |
|  | 11 | Hemoglobin subunit alpha | HBA_HUMAN | HBA1; HBA2 |
|  | 3 | Nucleoredoxin | NXN_HUMAN | NXN |
|  | 3 | Fibrinogen gamma chain | FIBG_HUMAN | FGG |
| **PD-NG,   T2D-NG,  T2D-PD** | 6 | Transaldolase | TALDO_HUMAN | TALDO1 |
|  | 5 | Dual specificity protein phosphatase 3 | DUS3_HUMAN | DUSP3 |

| **Comparison** | **Occurence** | **Protein description** | **UniProtKB identifier** | **Gene name** |
| --- | --- | --- | --- | --- |
| **T2D-NG** | 13 | Monocarboxylate transporter 4 | MOT4_HUMAN | SLC16A3 |
|  | 13 | Adenylyl cyclase-associated protein 1 | CAP1_HUMAN | CAP1 |
|  | 13 | Inorganic pyrophosphatase | IPYR_HUMAN | PPA1 |
|  | 12 | GMP reductase 1 | GMPR1_HUMAN | GMPR |
|  | 12 | Exportin-1 | XPO1_HUMAN | XPO1 |
|  | 12 | 40S ribosomal protein S12 | RS12_HUMAN | RPS12 |
|  | 12 | ATP synthase protein 8 | ATP8_HUMAN | MT-ATP8 |
|  | 11 | Protein S100-A4 | S10A4_HUMAN | S100A4 |
|  | 11 | Small muscular protein | SMPX_HUMAN | SMPX |
|  | 11 | ATP-dependent 6-phosphofructokinase, muscle type | PFKAM_HUMAN | PFKM |
|  | 9 | Ribonuclease inhibitor | RINI_HUMAN | RNH1 |
|  | 8 | Ras-related protein Rab-21 | RAB21_HUMAN | RAB21 |
|  | 8 | DnaJ homolog subfamily A member 2 | DNJA2_HUMAN | DNAJA2 |
|  | 8 | Aldehyde dehydrogenase, mitochondrial | ALDH2_HUMAN | ALDH2 |
|  | 7 | Ankyrin-3 | ANK3_HUMAN | ANK3 |
|  | 6 | EH domain-binding protein 1-like protein 1 | EH1L1_HUMAN | EHBP1L1 |
|  | 3 | Short/branched chain specific acyl-CoA dehydrogenase, mitochondrial | ACDSB_HUMAN | ACADSB |
|  | 3 | PDZ and LIM domain protein 1 | PDLI1_HUMAN | PDLIM1 |
|  | 3 | Calpain-3 | CAN3_HUMAN | CAPN3 |
|  | 2 | Myotrophin | MTPN_HUMAN | MTPN |
|  | 2 | Dihydrolipoyl dehydrogenase, mitochondrial | DLDH_HUMAN | DLD |
|  | 2 | Transaldolase | TALDO_HUMAN | TALDO1 |
|  | 2 | Protein-glutamine gamma-glutamyltransferase 2 | TGM2_HUMAN | TGM2 |
|  | 2 | Fructose-1,6-bisphosphatase isozyme 2 | F16P2_HUMAN | FBP2 |
|  | 2 | T-complex protein 1 subunit epsilon | TCPE_HUMAN | CCT5 |
|  | 1 | Hemopexin | HEMO_HUMAN | HPX |
|  | 1 | Myosin light chain 6B | MYL6B_HUMAN | MYL6B |
|  | 1 | Delta-1-pyrroline-5-carboxylate dehydrogenase, mitochondrial | AL4A1_HUMAN | ALDH4A1 |
|  | 1 | Nebulin-related-anchoring protein | NRAP_HUMAN | NRAP |
|  | 1 | Apolipoprotein A-I | APOA1_HUMAN | APOA1 |
|  | 1 | Transthyretin | TTHY_HUMAN | TTR |
|  | 1 | Dual specificity protein phosphatase 3 | DUS3_HUMAN | DUSP3 |
|  | 1 | Tubulin beta-4B chain | TBB4B_HUMAN | TUBB4B |
|  | 1 | Calcium-binding protein 39 | CAB39_HUMAN | CAB39 |
|  | 1 | Long-chain-fatty-acid--CoA ligase 3 | ACSL3_HUMAN | ACSL3 |
|  | 1 | Basement membrane-specific heparan sulfate proteoglycan core protein | PGBM_HUMAN | HSPG2 |
|  | 1 | Mitochondrial import inner membrane translocase subunit TIM44 | TIM44_HUMAN | TIMM44 |
|  | 1 | Creatine kinase B-type | KCRB_HUMAN | CKB |
|  | 1 | COP9 signalosome complex subunit 5 | CSN5_HUMAN | COPS5 |

| **Comparison** | **Occurence** | **Protein description** | **UniProtKB identifier** | **Gene name** |
| --- | --- | --- | --- | --- |
| **T2D-PD** | 31 | 3-ketoacyl-CoA thiolase, mitochondrial | THIM_HUMAN | ACAA2 |
|  | 28 | Talin-2 | TLN2_HUMAN | TLN2 |
|  | 24 | Peroxiredoxin-2 | PRDX2_HUMAN | PRDX2 |
|  | 19 | Serum amyloid P-component | SAMP_HUMAN | APCS |
|  | 12 | 40S ribosomal protein S12 | RS12_HUMAN | RPS12 |
|  | 11 | NADH dehydrogenase [ubiquinone] iron-sulfur protein 2, mitochondrial | NDUS2_HUMAN | NDUFS2 |
|  | 11 | Radixin | RADI_HUMAN | RDX |
|  | 10 | Tubulin beta-4B chain | TBB4B_HUMAN | TUBB4B |
|  | 8 | ATP-dependent 6-phosphofructokinase, muscle type | PFKAM_HUMAN | PFKM |
|  | 7 | 60S ribosomal protein L6 | RL6_HUMAN | RPL6 |
|  | 7 | Hemoglobin subunit alpha | HBA_HUMAN | HBA1; HBA2 |
|  | 7 | Glycogen debranching enzyme | GDE_HUMAN | AGL |
|  | 5 | Keratin, type II cytoskeletal 5 | K2C5_HUMAN | KRT5 |
|  | 4 | Cytochrome c oxidase subunit NDUFA4 | NDUA4_HUMAN | NDUFA4 |
|  | 3 | Aldo-keto reductase family 1 member A1 | AK1A1_HUMAN | AKR1A1 |
|  | 3 | Fructose-1,6-bisphosphatase 1 | F16P1_HUMAN | FBP1 |
|  | 3 | EH domain-binding protein 1-like protein 1 | EH1L1_HUMAN | EHBP1L1 |
|  | 3 | Lumican | LUM_HUMAN | LUM |
|  | 3 | Dual specificity mitogen-activated protein kinase kinase 1 | MP2K1_HUMAN | MAP2K1 |
|  | 3 | Alcohol dehydrogenase class-3 | ADHX_HUMAN | ADH5 |
|  | 2 | Aminopeptidase B | AMPB_HUMAN | RNPEP |
|  | 2 | Endonuclease domain-containing 1 protein | ENDD1_HUMAN | ENDOD1 |
|  | 2 | Immunoglobulin heavy constant gamma 1 | IGHG1_HUMAN | IGHG1 |
|  | 2 | Transaldolase | TALDO_HUMAN | TALDO1 |
|  | 2 | Dual specificity protein phosphatase 3 | DUS3_HUMAN | DUSP3 |
|  | 2 | NADH dehydrogenase [ubiquinone] iron-sulfur protein 5 | NDUS5_HUMAN | NDUFS5 |
|  | 1 | 4-trimethylaminobutyraldehyde dehydrogenase | AL9A1_HUMAN | ALDH9A1 |
|  | 1 | Electron transfer flavoprotein-ubiquinone oxidoreductase, mitochondrial | ETFD_HUMAN | ETFDH |
|  | 1 | 60S ribosomal protein L5 | RL5_HUMAN | RPL5 |
|  | 1 | Dystroglycan | DAG1_HUMAN | DAG1 |
|  | 1 | Receptor of activated protein C kinase 1 | RACK1_HUMAN | RACK1 |
|  | 1 | Elongation factor 1-gamma | EF1G_HUMAN | EEF1G |
|  | 1 | ATP synthase subunit O, mitochondrial | ATPO_HUMAN | ATP5PO |
|  | 1 | PDZ and LIM domain protein 1 | PDLI1_HUMAN | PDLIM1 |
|  | 1 | 60S acidic ribosomal protein P2 | RLA2_HUMAN | RPLP2 |
|  | 1 | Mitochondrial import inner membrane translocase subunit TIM44 | TIM44_HUMAN | TIMM44 |
|  | 1 | T-complex protein 1 subunit theta | TCPQ_HUMAN | CCT8 |
|  | 1 | ADP/ATP translocase 1 | ADT1_HUMAN | SLC25A4 |
|  | 1 | Immunoglobulin kappa constant | IGKC_HUMAN | IGKC |
|  | 1 | Nucleoredoxin | NXN_HUMAN | NXN |
|  | 1 | Cofilin-1 | COF1_HUMAN | CFL1 |
|  | 1 | Fibrinogen gamma chain | FIBG_HUMAN | FGG |
|  | 1 | Peroxiredoxin-1 | PRDX1_HUMAN | PRDX1 |
|  | 1 | Bifunctional purine biosynthesis protein ATIC | PUR9_HUMAN | ATIC |
|  | 1 | Transgelin | TAGL_HUMAN | TAGLN |

# Appendix H: Network diagram from String software


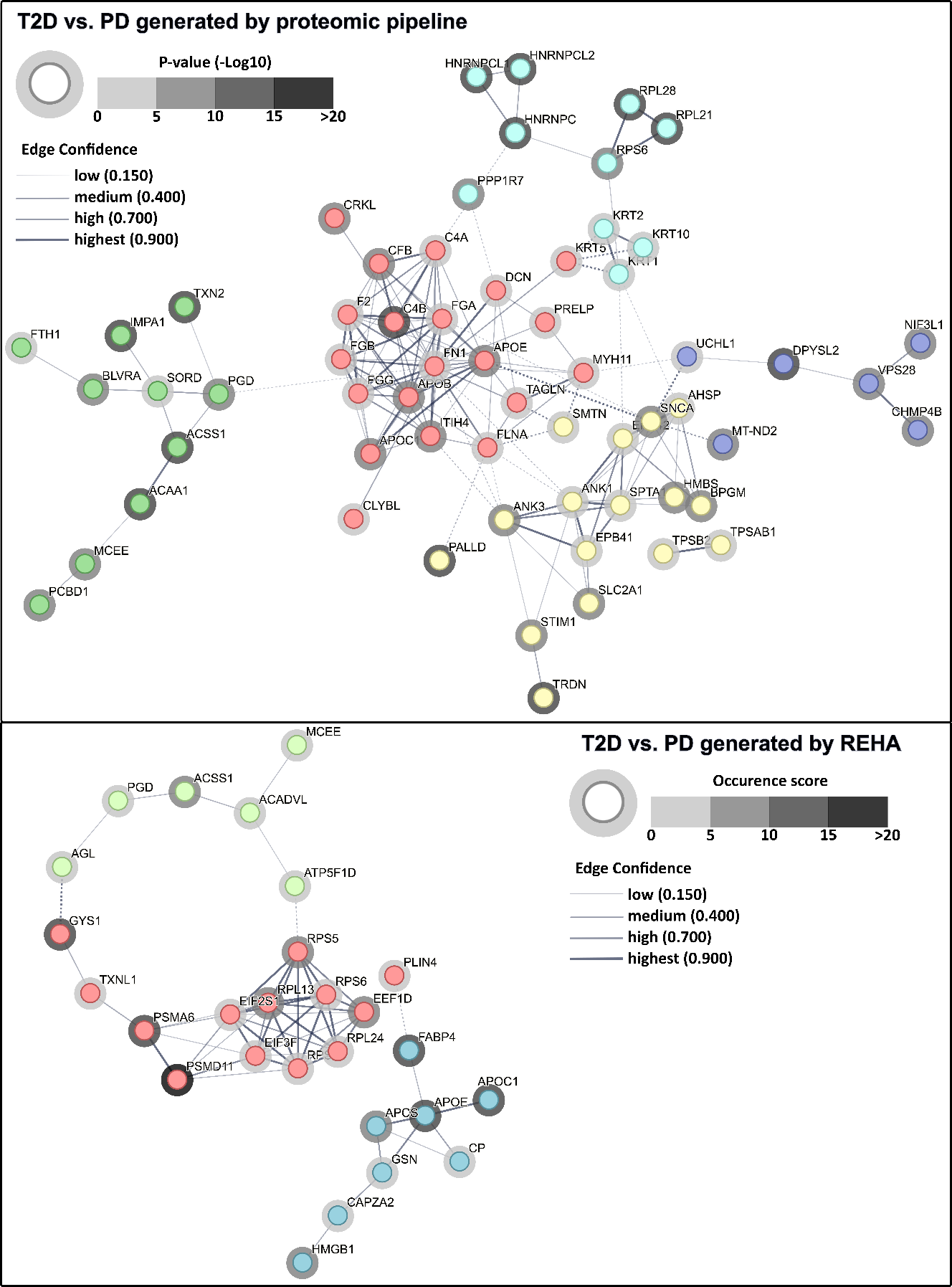


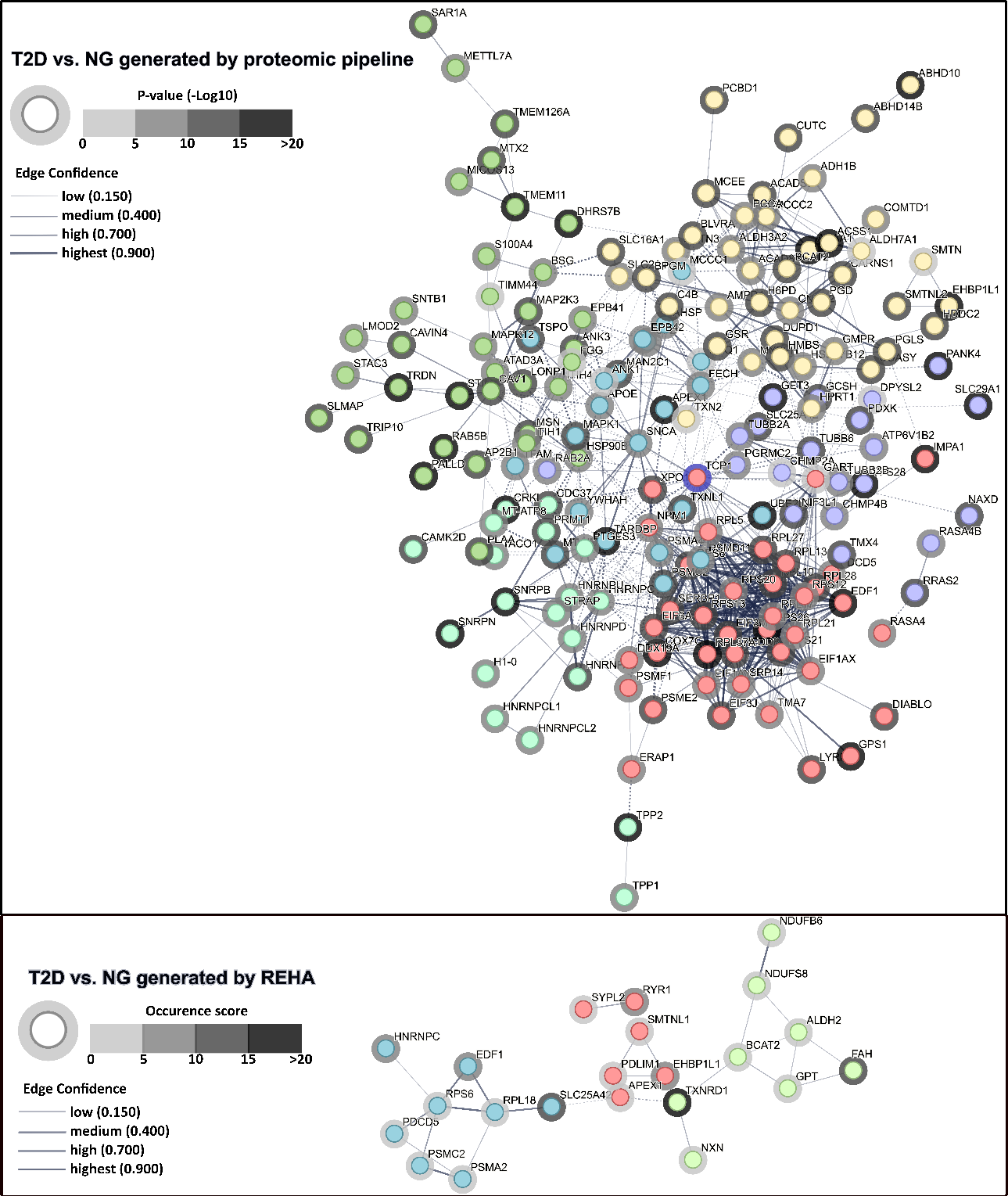


# Appendix I: Network diagram of REHA approach

Examining decision trees produced by the REHA algorithm unveils intricate networks of metabolic and cellular interplays across three comparisons: NG-PD, NG-T2D, and T2D-PD. The additional network diagram in the following Figure illustrates protein-protein interactions and comparisons, likely grouped based on the selected decision trees generated by REHA algorithm. It demonstrates how certain proteins are associated with the transition between NG, PD, and T2D states. Detailed information on each decision tree and the specific role and implications of each protein in metabolic disorders can be found in Appendix B and C, respectively.


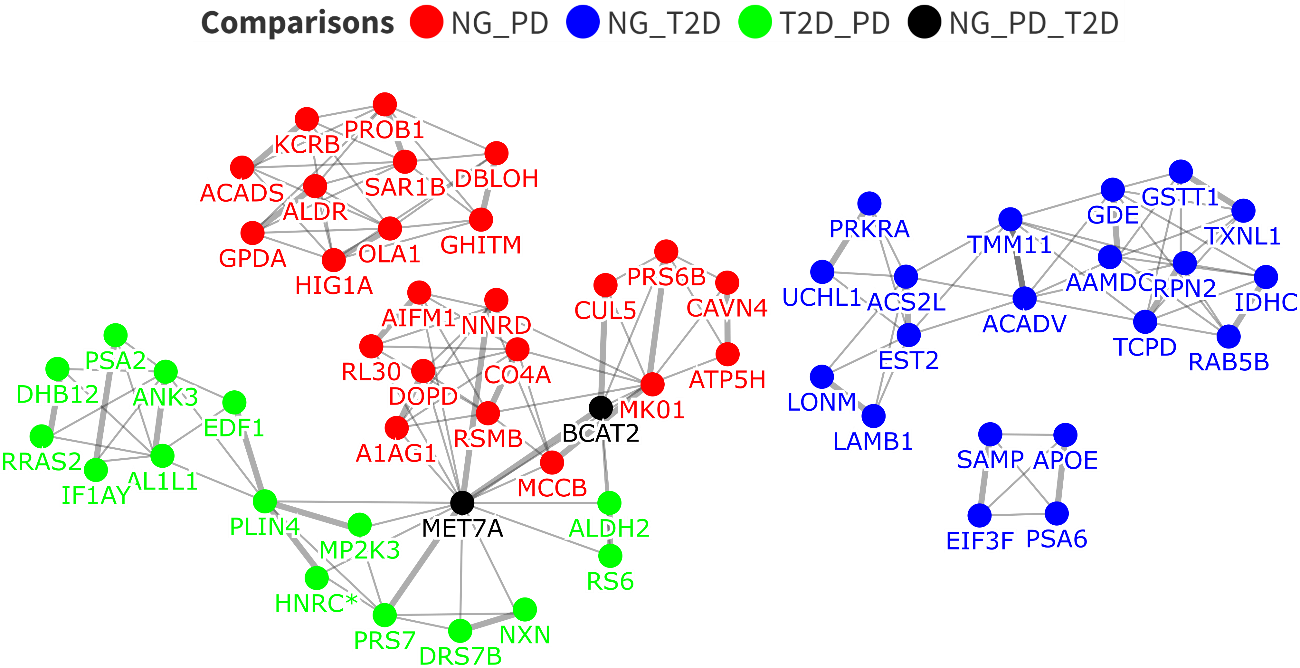


The decision trees for NG-PD primarily emphasize proteins associated with cellular metabolism, signaling, and energy homeostasis. Comparisons between NG and PD underscore the significance of 26S proteasome regulatory subunit 6B (PRS6B), Mitogen-activated protein kinase 1 (MK01), and mitochondrial ATP synthase subunit d (ATP5H), revealing the crucial roles of proteasomal activity, MAPK signaling, and mitochondrial function in the transition from normal glucose to prediabetes.

For NG-T2D, the selected proteins such as 40S ribosomal protein S6 (RS6), Aldehyde dehydrogenase, mitochondrial (ALDH2), and Methyltransferase-like protein 7A (MET7A) highlight a strong emphasis on ribosomal function, mitochondrial metabolism, and methylation processes. The focus on mitochondrial and ribosomal proteins suggests a concerted effort in cells to rebalance energy production and protein turnover as a means of reversing diabetic pathology.

In the context of T2D-PD, noteworthy proteins involved in mitochondrial function (Very long-chain specific acyl-CoA dehydrogenase; ACADV, Transmembrane protein 11; TMM11), oxidative stress response (Glutathione S-transferase theta-1 (GSTT1), Thioredoxin-like protein 1 (TXNL1)), and insulin signaling (Ras-related protein Rab-5B; RAB5B, Glycogen debranching enzyme; GDE) were employed. These choices underscore the significance of energy balance, oxidative stress management, and insulin pathway efficiency in the transition from a PD state to T2D. These proteins play a pivotal role in metabolic control and stress response, crucial for overcoming insulin resistance.

The analysis of proteomic data within human skeletal muscle unveiled several pathways associated with the development and biology of insulin resistance and diabetes, as well as regulated by exercise, including those of canonical exercise response kinases such as mitogen-activated protein kinases (MAPKs).

The tree structure, based on the comparative expression of these proteins, offers insight into the underlying biological processes that differentiate NG, PD, and T2D states. A cross-sectional view reveals both common threads and distinct features. This intricate interplay of proteins unveils a nuanced picture of how metabolic states evolve from normoglycemia to prediabetes and then to T2D, providing a window into the complex mechanisms at play.

Central to these findings is the role of mitochondrial function and energy metabolism, which emerge as consistent themes across the decision trees. This emphasizes the critical role of mitochondria in regulating the metabolic phenotype characteristic of type 2 diabetes. Additionally, proteins linked to stress response and proteostasis underscore the cellular adaptation to metabolic stress encountered in diabetes and prediabetes. Particularly noteworthy in the decision trees for T2D-PD comparisons is the focus on glucose metabolism and insulin signaling-related proteins, highlighting the fundamental role of insulin sensitivity in the dynamics of diabetes progression.

In conclusion, our findings reveal a diverse range of alterations in protein expression within exercise-induced insulin-resistant skeletal muscle, as elucidated by the REHA algorithm. The decision trees for each clinical scenario, namely NG-PD, NG-T2D, and T2D-PD, offer rich insights into potential interconnections among different pathways that may collectively contribute to the development of insulin resistance.

Our findings also suggest that REHA identified metabolic and proteomic markers that were overlooked by traditional statistical approaches. This includes proteins related to proteasomal activity, MAPK signaling, mitochondrial function, and insulin signaling pathways, which were not as prominently featured in the conventional analysis. These "biological switches" located usually at the root of the decision trees are meaningful in understanding the progression and regression of diabetes and prediabetic states, highlighting the utility of machine learning in elucidating complex biological relationships.

However, these patterns and the physiological significance of the cellular signaling modulation identified remain incompletely explored. Further investigation is required, especially considering the results of the external case study and Venn diagrams from the results section, which indicated variations in model performance when dealing with merged datasets. Additional research is needed to validate our findings in larger cohorts and diverse population groups, as well as to compare the effectiveness of REHA against other popular machine learning algorithms, both white and black box, in the context of proteomic data analysis.

Network diagram illustrating protein-protein interactions and comparisons, grouped based on the REHA machine learning approach for NG vs PD, NG vs T2D and T2D vs PD problems for external dataset case study. In analyzing the nine decision trees generated by the REHA algorithm a complex network of metabolic and cellular interactions is revealed for three comparisons (see Figure 8): NG-PD (normal glucose tolerance to prediabetes), NG-T2D (normal glucose tolerance to type 2 diabetes), and T2D-PD (type 2 diabetes to prediabetes). This complexity reflects the intricate nature of prediabetic states and diabetes. The additional network diagram demonstrates how certain proteins are associated with the transition between NG, PD, and T2D states. Thicker lines indicate direct protein interactions in the pair ordering relationship. Thinner lines represent secondary (hierarchical or vertical) connections with proteins that the REHA algorithm has highlighted due to their proximity in differentiating groups.

**
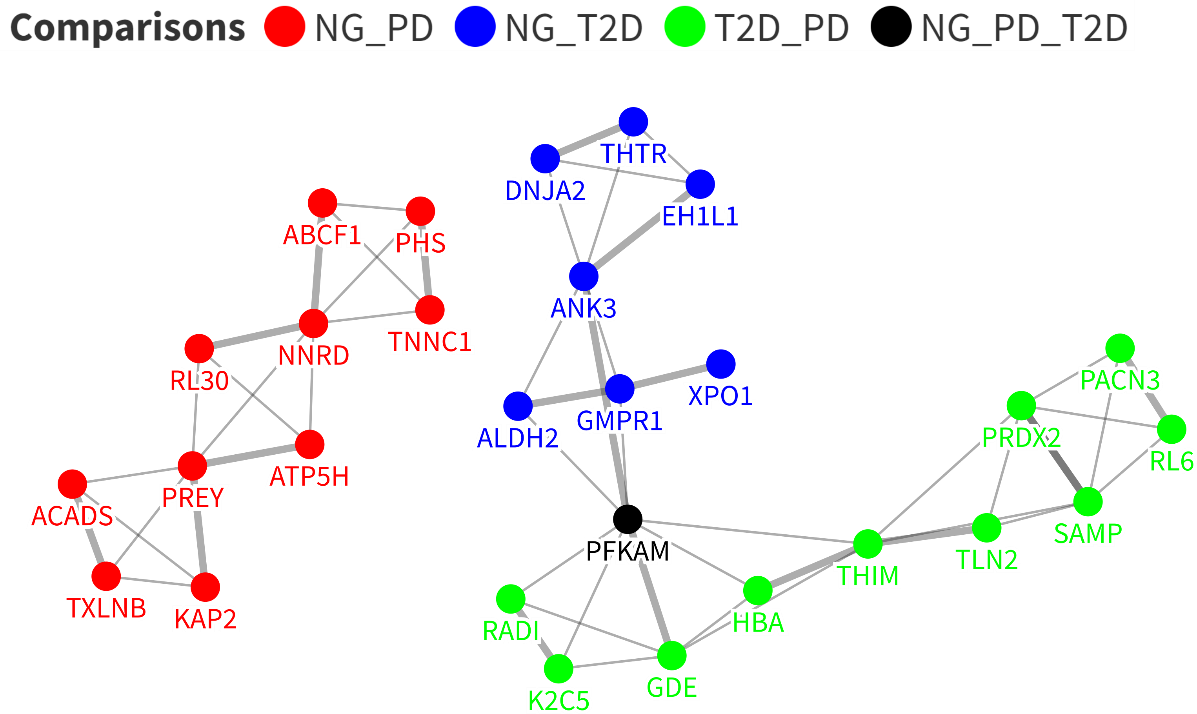
**

# Appendix J: ROC curves of REHA analysis

ROC curves of REHA analysis performed on original data illustrates the binary classifications using REHA: A) NG vs. PD, B) NG vs. T2D, and C) T2D vs. PD. The red lines represent the average ROC across 10-fold cross-validation. Also presented are combined performance metrics and their standard deviations for a full view of model performance.

**
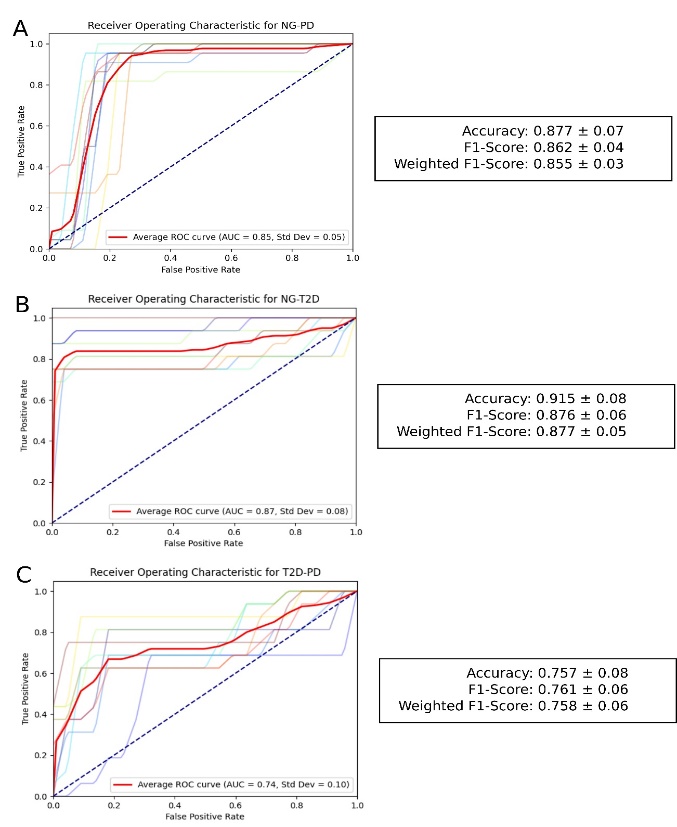

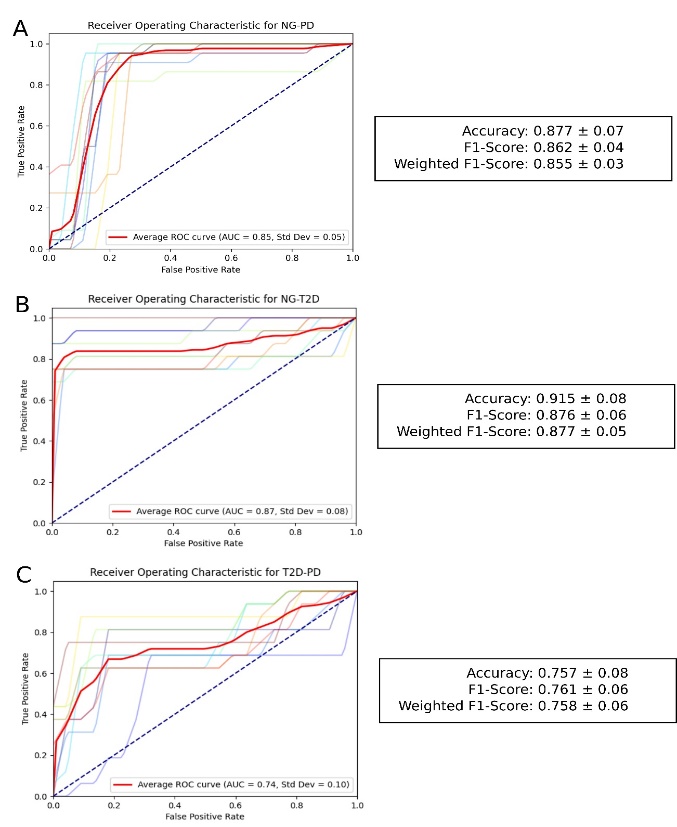
**

**
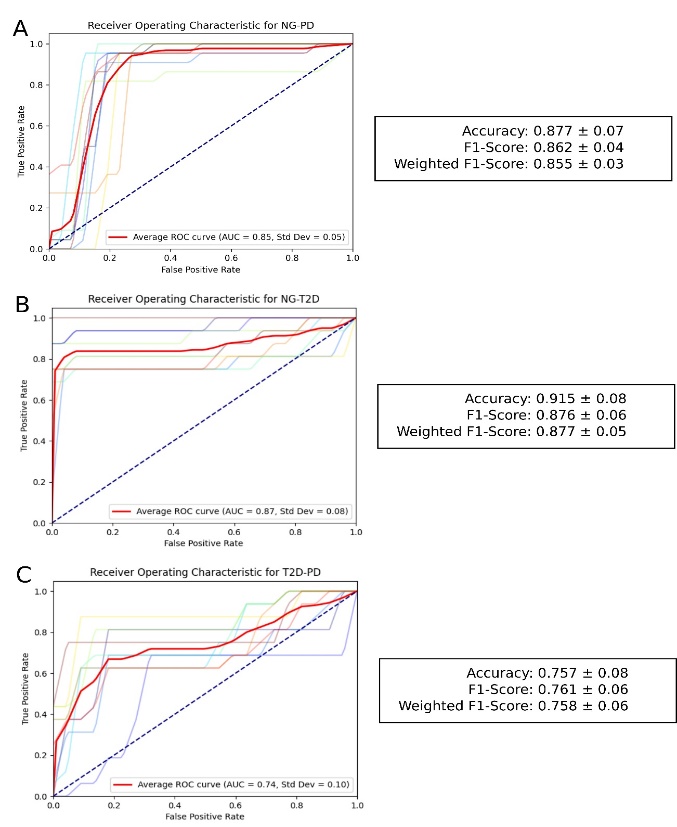
**

Following figure concerns performed external case study and shows averaged ROC curves across 10 fold CV for classifications using REHA on merged datasets. Also presented are combined performance metrics and their standard deviations for a full view of model performance.

# **
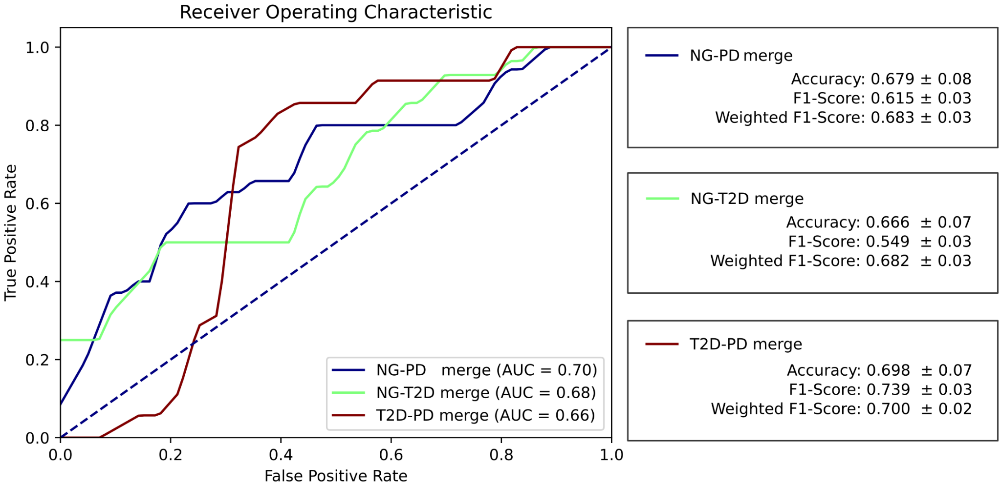
**Appendix K: References

1. Grøntved, A., Rimm, E. B., Willett, W. C., Andersen, L. B. & Hu, F. B. A prospective study of weight training and risk of type 2 diabetes mellitus in men. *Arch. Intern. Med.* **172**, 1306–1312 (2012).

2. Cuff, D. J. *et al.* Effective Exercise Modality to Reduce Insulin Resistance in Women With Type 2 Diabetes. *Diabetes Care* **26**, 2977–2982 (2003).

3. Sigal, R. J. *et al.* Effects of aerobic training, resistance training, or both on glycemic control in type 2 diabetes: a randomized trial. *Ann. Intern. Med.* **147**, 357–369 (2007).

4. BALKE, B. & WARE, R. W. An experimental study of physical fitness of Air Force personnel. *U. S. Armed Forces Med. J.* **10**, 675–688 (1959).

5. 2. Classification and Diagnosis of Diabetes: Standards of Medical Care in Diabetes-2020. *Diabetes Care* **43**, S14–S31 (2020).

6. Matthews, D. R. *et al.* Homeostasis model assessment: insulin resistance and beta-cell function from fasting plasma glucose and insulin concentrations in man. *Diabetologia* **28**, 412–419 (1985).

7. Shanely, R. A. *et al.* Human skeletal muscle biopsy procedures using the modified Bergström technique. *J. Vis. Exp.* 51812 (2014) doi:10.3791/51812.

8. León, I. R., Schwämmle, V., Jensen, O. N. & Sprenger, R. R. Quantitative assessment of in-solution digestion efficiency identifies optimal protocols for unbiased protein analysis. *Mol. Cell. Proteomics* **12**, 2992–3005 (2013).

9. Amodei, D. *et al.* Improving Precursor Selectivity in Data-Independent Acquisition Using Overlapping Windows. *J. Am. Soc. Mass Spectrom.* **30**, 669–684 (2019).

10. Pino, L. K., Just, S. C., MacCoss, M. J. & Searle, B. C. Acquiring and Analyzing Data Independent Acquisition Proteomics Experiments without Spectrum Libraries. *Mol. Cell. Proteomics* **19**, 1088–1103 (2020).

11. Searle, B. C. *et al.* Chromatogram libraries improve peptide detection and quantification by data independent acquisition mass spectrometry. *Nat. Commun.* **9**, 5128 (2018).

12. Searle, B. C. *et al.* Generating high quality libraries for DIA MS with empirically corrected peptide predictions. *Nat. Commun.* **11**, 1548 (2020).

13. Kanayama, H. O. *et al.* Demonstration that a human 26S proteolytic complex consists of a proteasome and multiple associated protein components and hydrolyzes ATP and ubiquitin-ligated proteins by closely linked mechanisms. *Eur. J. Biochem.* **206**, 567–578 (1992).

14. Al-Khalili, L. *et al.* Proteasome inhibition in skeletal muscle cells unmasks metabolic derangements in type 2 diabetes. *Am. J. Physiol. Cell Physiol.* **307**, C774-87 (2014).

15. Wang, X., Hu, Z., Hu, J., Du, J. & Mitch, W. E. Insulin resistance accelerates muscle protein degradation: Activation of the ubiquitin-proteasome pathway by defects in muscle cell signaling. *Endocrinology* **147**, 4160–4168 (2006).

16. Hwang, H. *et al.* Proteomics analysis of human skeletal muscle reveals novel abnormalities in obesity and type 2 diabetes. *Diabetes* **59**, 33–42 (2010).

17. Arkun, Y. Dynamic Modeling and Analysis of the Cross-Talk between Insulin/AKT and MAPK/ERK Signaling Pathways. *PLoS One* **11**, e0149684 (2016).

18. Formentini, L. *et al.* Mitochondrial H(+)-ATP synthase in human skeletal muscle: contribution to dyslipidaemia and insulin resistance. *Diabetologia* **60**, 2052–2065 (2017).

19. Oh, Y. S. *et al.* Exercise type and muscle fiber specific induction of caveolin-1 expression for insulin sensitivity of skeletal muscle. *Exp. Mol. Med.* **39**, 395–401 (2007).

20. Haddad, D., Al Madhoun, A., Nizam, R. & Al-Mulla, F. Role of Caveolin-1 in Diabetes and Its Complications. *Oxid. Med. Cell. Longev.* **2020**, 9761539 (2020).

21. Bano, I. *et al.* A Comprehensive Review of Biological Roles and Interactions of Cullin-5 Protein. *ACS omega* **7**, 5615–5624 (2022).

22. Chen, C. *et al.* Cullin neddylation inhibitor attenuates hyperglycemia by enhancing hepatic insulin signaling through insulin receptor substrate stabilization. *Proc. Natl. Acad. Sci. U. S. A.* **119**, (2022).

23. Jiang, P. *et al.* Transcriptomic Analysis of Short/Branched-Chain Acyl-Coenzyme a Dehydrogenase Knocked Out bMECs Revealed Its Regulatory Effect on Lipid Metabolism. *Front. Vet. Sci.* **8**, 744287 (2021).

24. Zong, L. & Liang, Z. Apoptosis-inducing factor: a mitochondrial protein associated with metabolic diseases-a narrative review. *Cardiovasc. Diagn. Ther.* **13**, 609–622 (2023).

25. Hu, J. J. *et al.* Discovery, structure, and function of filamentous 3-methylcrotonyl-CoA carboxylase. *Structure* **31**, 100-110.e4 (2023).

26. Meierhofer, D., Halbach, M., Şen, N. E., Gispert, S. & Auburger, G. Ataxin-2 (Atxn2)-Knock-Out Mice Show Branched Chain Amino Acids and Fatty Acids Pathway Alterations. *Mol. Cell. Proteomics* **15**, 1728–1739 (2016).

27. de Medeiros, S. F., de Medeiros, M. A. S., Barbosa, B. B., Yamamoto, A. K. L. W. & Yamamoto, M. M. W. The connection of alpha-1 acid glycoprotein inflammatory marker with anthropometric, hormonal, and metabolic characteristic of women with polycystic ovary syndrome. *J. Obstet. Gynaecol. Res.* **47**, 3571–3582 (2021).

28. Qi, D. *et al.* The vestigial enzyme D-dopachrome tautomerase protects the heart against ischemic injury. *J. Clin. Invest.* **124**, 3540–3550 (2014).

29. Campeanu, I. J. *et al.* Multi-omics integration of methyltransferase-like protein family reveals clinical outcomes and functional signatures in human cancer. *Sci. Rep.* **11**, 14784 (2021).

30. Marbaix, A. Y. *et al.* Occurrence and subcellular distribution of the NADPHX repair system in mammals. *Biochem. J.* **460**, 49–58 (2014).

31. Van Bergen, N. J. *et al.* NAD(P)HX dehydratase (NAXD) deficiency: a novel neurodegenerative disorder exacerbated by febrile illnesses. *Brain* **142**, 50–58 (2019).

32. Shim, K., Begum, R., Yang, C. & Wang, H. Complement activation in obesity, insulin resistance, and type 2 diabetes mellitus. *World J. Diabetes* **11**, 1–12 (2020).

33. Will, C. L. & Lührmann, R. Spliceosome structure and function. *Cold Spring Harb. Perspect. Biol.* **3**, (2011).

34. White, P. J. *et al.* Insulin action, type 2 diabetes, and branched-chain amino acids: A two-way street. *Mol. Metab.* **52**, 101261 (2021).

35. Field, M. L., Khan, O., Abbaraju, J. & Clark, J. F. Functional compartmentation of glycogen phosphorylase with creatine kinase and Ca2+ ATPase in skeletal muscle. *J. Theor. Biol.* **238**, 257–268 (2006).

36. Bekkelund, S. I. Leisure physical exercise and creatine kinase activity. The Tromsø study. *Scand. J. Med. Sci. Sports* **30**, 2437–2444 (2020).

37. Bekkelund, S. I. Creatine kinase is associated with glycated haemoglobin in a nondiabetic population. The Tromsø study. *PLoS One* **18**, e0281239 (2023).

38. Orliaguet, L. *et al.* Early macrophage response to obesity encompasses Interferon Regulatory Factor 5 regulated mitochondrial architecture remodelling. *Nat. Commun.* **13**, 5089 (2022).

39. Du, C., Fang, M., Li, Y., Li, L. & Wang, X. Smac, a mitochondrial protein that promotes cytochrome c-dependent caspase activation by eliminating IAP inhibition. *Cell* **102**, 33–42 (2000).

40. Harding, J. W. J., Pyeritz, E. A., Copeland, E. S. & White, H. B. 3rd. Role of glycerol 3-phosphate dehydrogenase in glyceride metabolism. Effect of diet on enzyme activities in chicken liver. *Biochem. J.* **146**, 223–229 (1975).

41. Pastel, E., Pointud, J.-C., Volat, F., Martinez, A. & Lefrançois-Martinez, A.-M. Aldo-Keto Reductases 1B in Endocrinology and Metabolism. *Front. Pharmacol.* **3**, 148 (2012).

42. Marcil, V. *et al.* Tissue distribution and regulation of the small Sar1b GTPase in mice. *Cell. Physiol. Biochem. Int. J. Exp. Cell. Physiol. Biochem. Pharmacol.* **33**, 1815–1826 (2014).

43. Karolak, J. A. *et al.* Variants in SKP1, PROB1, and IL17B genes at keratoconus 5q31.1-q35.3 susceptibility locus identified by whole-exome sequencing. *Eur. J. Hum. Genet.* **25**, 73–78 (2017).

44. Lin, T.-F. *et al.* Association of Common Variants in OLA1 Gene with Preclinical Atherosclerosis. *Int. J. Mol. Sci.* **23**, (2022).

45. Jia, Y. zhao *et al.* HIG1 domain family member 1A is a crucial regulator of disorders associated with hypoxia. *Mitochondrion* **69**, 171–182 (2023).

46. Harris, T. E. *et al.* mTOR-dependent stimulation of the association of eIF4G and eIF3 by insulin. *EMBO J.* **25**, 1659–1668 (2006).

47. Li, S.-Y., Gilbert, S. A. B., Li, Q. & Ren, J. Aldehyde dehydrogenase-2 (ALDH2) ameliorates chronic alcohol ingestion-induced myocardial insulin resistance and endoplasmic reticulum stress. *J. Mol. Cell. Cardiol.* **47**, 247–255 (2009).

48. Lee, E., Kim, J., Kim, T.-K., Park, S.-Y. & Im, G.-I. Methyltransferase-like protein 7A (METTL7A) promotes cell survival and osteogenic differentiation under metabolic stress. *Cell Death Discov.* **7**, 154 (2021).

49. Mo, L. *et al.* An analysis of the role of HnRNP C dysregulation in cancers. *Biomark. Res.* **10**, 19 (2022).

50. Zhao, M. *et al.* Loss of hnRNP A1 in murine skeletal muscle exacerbates high-fat diet-induced onset of insulin resistance and hepatic steatosis. *J. Mol. Cell Biol.* **12**, 277–290 (2020).

51. Pourteymour, S. *et al.* Perilipin 4 in human skeletal muscle: localization and effect of physical activity. *Physiol. Rep.* **3**, (2015).

52. Raingeaud, J., Whitmarsh, A. J., Barrett, T., Dérijard, B. & Davis, R. J. MKK3- and MKK6-regulated gene expression is mediated by the p38 mitogen-activated protein kinase signal transduction pathway. *Mol. Cell. Biol.* **16**, 1247–1255 (1996).

53. Cariolato, L., Cavin, S. & Diviani, D. A-kinase anchoring protein (AKAP)-Lbc anchors a PKN-based signaling complex involved in α1-adrenergic receptor-induced p38 activation. *J. Biol. Chem.* **286**, 7925–7937 (2011).

54. Idelfonso-García, O. G. *et al.* Is Nucleoredoxin a Master Regulator of Cellular Redox Homeostasis? Its Implication in Different Pathologies. *Antioxidants 2022, Vol. 11, Page 670* **11**, 670 (2022).

55. Bahn, Y. J. *et al.* Nucleoredoxin promotes adipogenic differentiation through regulation of wnt/β -catenin signaling. *J. Lipid Res.* **56**, 294–303 (2015).

56. Stambergova, H., Skarydova, L., Dunford, J. E. & Wsol, V. Biochemical properties of human dehydrogenase/reductase (SDR family) member 7. *Chem. Biol. Interact.* **207**, 52–57 (2014).

57. Ballabio, E., Mariotti, M., De Benedictis, L. & Maier, J. A. M. The dual role of endothelial differentiation-related factor-1 in the cytosol and nucleus: modulation by protein kinase A. *Cell. Mol. Life Sci.* **61**, 1069–1074 (2004).

58. Groettrup, M. *et al.* A role for the proteasome regulator PA28alpha in antigen presentation. *Nature* **381**, 166–168 (1996).

59. Chaudhuri, J., Si, K. & Maitra, U. Function of eukaryotic translation initiation factor 1A (eIF1A) (formerly called eIF-4C) in initiation of protein synthesis. *J. Biol. Chem.* **272**, 7883–7891 (1997).

60. Mor, A., Aizman, E., George, J. & Kloog, Y. Ras inhibition induces insulin sensitivity and glucose uptake. *PLoS One* **6**, e21712 (2011).

61. Moon, Y.-A. & Horton, J. D. Identification of two mammalian reductases involved in the two-carbon fatty acyl elongation cascade. *J. Biol. Chem.* **278**, 7335–7343 (2003).

62. Krupenko, N. I. *et al.* Cytosolic 10-formyltetrahydrofolate dehydrogenase regulates glycine metabolism in mouse liver. *Sci. Rep.* **9**, 14937 (2019).

63. Chagula, D. B., Rechciński, T., Rudnicka, K. & Chmiela, M. Ankyrins in human health and disease - an update of recent experimental findings. *Arch. Med. Sci.* **16**, 715–726 (2020).

64. Lorenzo, D. N. & Bennett, V. Cell-autonomous adiposity through increased cell surface GLUT4 due to ankyrin-B deficiency. *Proc. Natl. Acad. Sci. U. S. A.* **114**, 12743–12748 (2017).

65. Anwer, M. & Iqbal, M. J. Serum Amyloid P and Endocrine Markers in a Cohort of Obese Children. *Indian J. Endocrinol. Metab.* **22**, 683–688 (2018).

66. Gomes-Duarte, A., Lacerda, R., Menezes, J. & Romão, L. eIF3: a factor for human health and disease. *RNA Biol.* **15**, 26–34 (2018).

67. Chouinard-Watkins, R. & Plourde, M. Fatty Acid Metabolism in Carriers of Apolipoprotein E Epsilon 4 Allele: Is It Contributing to Higher Risk of Cognitive Decline and Coronary Heart Disease? *Nutr. 2014, Vol. 6, Pages 4452-4471* **6**, 4452–4471 (2014).

68. Zhang, D. *et al.* Resistance to high-fat diet-induced obesity and insulin resistance in mice with very long-chain acyl-CoA dehydrogenase deficiency. *Cell Metab.* **11**, 402–411 (2010).

69. Gok, M. O. *et al.* The outer mitochondrial membrane protein TMEM11 demarcates spatially restricted BNIP3/BNIP3L-mediated mitophagy. *J. Cell Biol.* **222**, (2023).

70. Altun, Ö. *et al.* The association of laminin levels with insulin resistance and non-alcoholic hepatosteatosis. *Diabetol. Metab. Syndr.* **13**, 1–7 (2021).

71. Lee, J. H. *et al.* Expression of LONP1 Is High in Visceral Adipose Tissue in Obesity, and Is Associated with Glucose and Lipid Metabolism. *Endocrinol. Metab. (Seoul, Korea)* **36**, 661–671 (2021).

72. Bishop, P., Rocca, D. & Henley, J. M. Ubiquitin C-terminal hydrolase L1 (UCH-L1): structure, distribution and roles in brain function and dysfunction. *Biochem. J.* **473**, 2453–2462 (2016).

73. Nakamura, T. *et al.* Double-stranded RNA-dependent protein kinase links pathogen sensing with stress and metabolic homeostasis. *Cell* **140**, 338–348 (2010).

74. Pindel, E. V *et al.* Purification and cloning of a broad substrate specificity human liver carboxylesterase that catalyzes the hydrolysis of cocaine and heroin. *J. Biol. Chem.* **272**, 14769–14775 (1997).

75. Ling, R. *et al.* Acetyl-CoA synthetase 2(ACSS2): a review with a focus on metabolism and tumor development. *Discov. Oncol.* **13**, 58 (2022).

76. Ghosh Dastidar, S. *et al.* Glutathione S-transferase P deficiency induces glucose intolerance via JNK-dependent enhancement of hepatic gluconeogenesis. *Am. J. Physiol. Endocrinol. Metab.* **315**, E1005–E1018 (2018).

77. Wondafrash, D. Z. *et al.* Thioredoxin-Interacting Protein as a Novel Potential Therapeutic Target in Diabetes Mellitus and Its Underlying Complications. *Diabetes. Metab. Syndr. Obes.* **13**, 43–51 (2020).

78. Cha, H. J. *et al.* Tat-Thioredoxin-like protein 1 attenuates ischemic brain injury by regulation of MAPKs and apoptosis signaling. *BMB reports* vol. 56 234–239 (2023).

79. Koh, H.-J. *et al.* Cytosolic NADP+-dependent isocitrate dehydrogenase plays a key role in lipid metabolism. *J. Biol. Chem.* **279**, 39968–39974 (2004).

80. Golden, E. *et al.* The oncogene AAMDC links PI3K-AKT-mTOR signaling with metabolic reprograming in estrogen receptor-positive breast cancer. *Nat. Commun.* **12**, 1920 (2021).

81. Ashcroft, F. M., Rohm, M., Clark, A. & Brereton, M. F. Is Type 2 Diabetes a Glycogen Storage Disease of Pancreatic β Cells? *Cell Metab.* **26**, 17–23 (2017).

82. Freund, A. *et al.* Proteostatic control of telomerase function through TRiC-mediated folding of TCAB1. *Cell* **159**, 1389–1403 (2014).

83. Seo, S. *et al.* BBS6, BBS10, and BBS12 form a complex with CCT/TRiC family chaperonins and mediate BBSome assembly. *Proc. Natl. Acad. Sci. U. S. A.* **107**, 1488–1493 (2010).

84. Liu, H., Yu, S., Xu, W. & Xu, J. Enhancement of 26S proteasome functionality connects oxidative stress and vascular endothelial inflammatory response in diabetes mellitus. *Arterioscler. Thromb. Vasc. Biol.* **32**, 2131–2140 (2012).

85. Cheroni, C. *et al.* Functional alterations of the ubiquitin-proteasome system in motor neurons of a mouse model of familial amyotrophic lateral sclerosis. *Hum. Mol. Genet.* **18**, 82–96 (2009).

86. Ciechanover, A. & Brundin, P. The ubiquitin proteasome system in neurodegenerative diseases: sometimes the chicken, sometimes the egg. *Neuron* **40**, 427–446 (2003).

87. Bastiani, M. *et al.* MURC/Cavin-4 and cavin family members form tissue-specific caveolar complexes. *J. Cell Biol.* **185**, 1259–1273 (2009).

88. Stadler, S. C. *et al.* Newborn screening for 3-methylcrotonyl-CoA carboxylase deficiency: population heterogeneity of MCCA and MCCB mutations and impact on risk assessment. *Hum. Mutat.* **27**, 748–759 (2006).

89. Morscher, R. J. *et al.* A single mutation in MCCC1 or MCCC2 as a potential cause of positive screening for 3-methylcrotonyl-CoA carboxylase deficiency. *Mol. Genet. Metab.* **105**, 602–606 (2012).

90. Grünert, S. C. *et al.* 3-methylcrotonyl-CoA carboxylase deficiency: clinical, biochemical, enzymatic and molecular studies in 88 individuals. *Orphanet J. Rare Dis.* **7**, 31 (2012).

91. Cozzolino, C. *et al.* Biochemical and molecular characterization of 3-Methylcrotonylglycinuria in an Italian asymptomatic girl. *Genet. Mol. Biol.* **41**, 379–385 (2018).

92. Zandberg, L., van Dyk, H. C., van der Westhuizen, F. H. & van Dijk, A. A. A 3-methylcrotonyl-CoA carboxylase deficient human skin fibroblast transcriptome reveals underlying mitochondrial dysfunction and oxidative stress. *Int. J. Biochem. Cell Biol.* **78**, 116–129 (2016).

93. He, J. *et al.* Methylcrotonoyl-CoA Carboxylase 2 Promotes Proliferation, Migration and Invasion and Inhibits Apoptosis of Prostate Cancer Cells Through Regulating GLUD1-P38 MAPK Signaling Pathway. *Onco. Targets. Ther.* **13**, 7317–7327 (2020).

94. Hochepied, T., Berger, F. G., Baumann, H. & Libert, C. Alpha(1)-acid glycoprotein: an acute phase protein with inflammatory and immunomodulating properties. *Cytokine Growth Factor Rev.* **14**, 25–34 (2003).

95. Odh, G., Hindemith, A., Rosengren, A. M., Rosengren, E. & Rorsman, H. Isolation of a new tautomerase monitored by the conversion of D-dopachrome to 5,6-dihydroxyindole. *Biochem. Biophys. Res. Commun.* **197**, 619–624 (1993).

96. Zan, C., Yang, B., Brandhofer, M., El Bounkari, O. & Bernhagen, J. D-dopachrome tautomerase in cardiovascular and inflammatory diseases-A new kid on the block or just another MIF? *FASEB J. Off. Publ. Fed. Am. Soc. Exp. Biol.* **36**, e22601 (2022).

97. McMillan, D. E. Elevation of complement components in diabetes mellitus. *Diabete Metab.* **6**, 265–270 (1980).

98. Nie, J. *et al.* Resting and post-exercise serum biomarkers of cardiac and skeletal muscle damage in adolescent runners. *Scand. J. Med. Sci. Sports* **21**, 625–629 (2011).

99. Kenney, K. *et al.* Serum creatine kinase after exercise: drawing the line between physiological response and exertional rhabdomyolysis. *Muscle Nerve* **45**, 356–362 (2012).

100. Ou, X. *et al.* Crystal structures of human glycerol 3-phosphate dehydrogenase 1 (GPD1). *J. Mol. Biol.* **357**, 858–869 (2006).

101. Eto, K. *et al.* Role of NADH shuttle system in glucose-induced activation of mitochondrial metabolism and insulin secretion. *Science* **283**, 981–985 (1999).

102. Thakur, S. *et al.* Metformin Targets Mitochondrial Glycerophosphate Dehydrogenase to Control Rate of Oxidative Phosphorylation and Growth of Thyroid Cancer In Vitro and In Vivo. *Clin. cancer Res. an Off. J. Am. Assoc. Cancer Res.* **24**, 4030–4043 (2018).

103. Langston, P. K. *et al.* Glycerol phosphate shuttle enzyme GPD2 regulates macrophage inflammatory responses. *Nat. Immunol.* **20**, 1186–1195 (2019).

104. Zheng, Y. *et al.* Deficiency of Mitochondrial Glycerol 3-Phosphate Dehydrogenase Contributes to Hepatic Steatosis. *Hepatology* **70**, 84–97 (2019).

105. Liu, X. *et al.* Mitochondrial glycerol 3-phosphate dehydrogenase promotes skeletal muscle regeneration. *EMBO Mol. Med.* **10**, (2018).

106. Qu, H. *et al.* Deficiency of Mitochondrial Glycerol 3-Phosphate Dehydrogenase Exacerbates Podocyte Injury and the Progression of Diabetic Kidney Disease. *Diabetes* **70**, 1372–1387 (2021).

107. Auclair, N. *et al.* High-fat diet reveals the impact of Sar1b defects on lipid and lipoprotein profile and cholesterol metabolism. *J. Lipid Res.* **64**, 100423 (2023).

108. Li, J. *et al.* Aldehyde dehydrogenase 2 alleviates mitochondrial dysfunction by promoting PGC-1α-mediated biogenesis in acute kidney injury. *Cell Death Dis.* **14**, 45 (2023).

109. Gui, W. *et al.* LncRNAH19 improves insulin resistance in skeletal muscle by regulating heterogeneous nuclear ribonucleoprotein A1. *Cell Commun. Signal.* **18**, 173 (2020).

110. Krupenko, N. I. *et al.* Aldh1l2 knockout mouse metabolomics links the loss of the mitochondrial folate enzyme to deregulation of a lipid metabolism observed in rare human disorder. *Hum. Genomics* **14**, 41 (2020).

111. Lorenzo, D. N. *et al.* Ankyrin-B metabolic syndrome combines age-dependent adiposity with pancreatic β cell insufficiency. *J. Clin. Invest.* **125**, 3087–3102 (2015).

112. Huang, Y. & Mahley, R. W. Apolipoprotein E: Structure and function in lipid metabolism, neurobiology, and Alzheimer’s diseases. *Neurobiol. Dis.* **72**, 3–12 (2014).

113. Kawashima, Y. *et al.* Apolipoprotein e deficiency abrogates insulin resistance in a mouse model of type 2 diabetes mellitus. *Diabetologia* **52**, 1434–1441 (2009).

114. McAndrew, R. P. *et al.* Structural basis for substrate fatty acyl chain specificity: crystal structure of human very-long-chain acyl-CoA dehydrogenase. *J. Biol. Chem.* **283**, 9435–9443 (2008).

115. He, M. *et al.* Identification and characterization of new long chain acyl-CoA dehydrogenases. *Mol. Genet. Metab.* **102**, 418–429 (2011).

116. Xie, S. *et al.* Inactivation of lipid glyceryl ester metabolism in human THP1 monocytes/macrophages by activated organophosphorus insecticides: role of carboxylesterases 1 and 2. *Chem. Res. Toxicol.* **23**, 1890–1904 (2010).

117. Tars, K. *et al.* Structural basis of the suppressed catalytic activity of wild-type human glutathione transferase T1-1 compared to its W234R mutant. *J. Mol. Biol.* **355**, 96–105 (2006).

118. Shokeer, A. & Mannervik, B. Residue 234 is a master switch of the alternative-substrate activity profile of human and rodent theta class glutathione transferase T1-1. *Biochim. Biophys. Acta* **1800**, 466–473 (2010).

119. Reska, D. *et al.* Integration of solutions and services for multi-omics data analysis towards personalized medicine. *Biocybern. Biomed. Eng.* **41**, 1646–1663 (2021).
